# Environmental factors shaping bacterial, archaeal and fungal community structure in hydrothermal sediments of Guaymas Basin, Gulf of California

**DOI:** 10.1371/journal.pone.0256321

**Published:** 2021-09-08

**Authors:** Gustavo A. Ramírez, Paraskevi Mara, Taylor Sehein, Gunter Wegener, Christopher R. Chambers, Samantha B. Joye, Richard N. Peterson, Aurélie Philippe, Gaëtan Burgaud, Virginia P. Edgcomb, Andreas P. Teske

**Affiliations:** 1 Department of Marine Sciences, University of North Carolina at Chapel Hill, NC, United States of America; 2 Department of Marine Biology, Leon H. Charney School of Marine Sciences, University of Haifa, Haifa, Israel; 3 College of Veterinary Medicine, Western University of Health Sciences, Pomona, CA, United States of America; 4 Geology and Geophysics Dept., Woods Hole Oceanographic Institution, Woods Hole, MA, United States of America; 5 MARUM, Center for Marine Environmental Sciences, University Bremen, Germany; 6 Max-Planck-Institute for Marine Microbiology, Bremen, Germany; 7 Department of Marine Sciences, University of Georgia, Athens, GA, United States of America; 8 School of Coastal and Marine Systems Science, Coastal Carolina University, Conway, SC, United States of America; 9 Univ. Brest, Laboratoire Universitaire de Biodiversité et Ecologie Microbienne, Plouzané, France; University of Utah, UNITED STATES

## Abstract

The flanking regions of Guaymas Basin, a young marginal rift basin located in the Gulf of California, are covered with thick sediment layers that are hydrothermally altered due to magmatic intrusions. To explore environmental controls on microbial community structure in this complex environment, we analyzed site- and depth-related patterns of microbial community composition (bacteria, archaea, and fungi) in hydrothermally influenced sediments with different thermal conditions, geochemical regimes, and extent of microbial mats. We compared communities in hot hydrothermal sediments (75-100°C at ~40 cm depth) covered by orange-pigmented *Beggiatoaceae* mats in the Cathedral Hill area, temperate sediments (25-30°C at ~40 cm depth) covered by yellow sulfur precipitates and filamentous sulfur oxidizers at the Aceto Balsamico location, hot sediments (>115°C at ~40 cm depth) with orange-pigmented mats surrounded by yellow and white mats at the Marker 14 location, and background, non-hydrothermal sediments (3.8°C at ~45 cm depth) overlain with ambient seawater. Whereas bacterial and archaeal communities are clearly structured by site-specific *in-situ* thermal gradients and geochemical conditions, fungal communities are generally structured by sediment depth. Unexpectedly, chytrid sequence biosignatures are ubiquitous in surficial sediments whereas deeper sediments contain diverse yeasts and filamentous fungi. In correlation analyses across different sites and sediment depths, fungal phylotypes correlate to each other to a much greater degree than Bacteria and Archaea do to each other or to fungi, further substantiating that site-specific *in-situ* thermal gradients and geochemical conditions that control bacteria and archaea do not extend to fungi.

## Introduction

In the Guaymas Basin spreading center, located in the central Gulf of California, hydrothermally influenced sediments are characterized by steep thermal gradients that may reach the thermal limits of life in just a few centimeters depth [1, 2]. The extreme temperatures transform buried organic matter into complex mixtures of dissolved inorganic carbon (DIC), methane, ammonia, organic acids, short chain alkanes, and aromatic compounds that migrate to shallow sediments and sustain diverse and active microbial communities [3–6].

Among the surface expressions of hydrothermalism, microbial mats are widespread and conspicuous [7] and provide visual markers for hydrothermally active sediments poised for sampling by submersible [2]. Previous works in Guaymas Basin have explored the ecophysiology and genomics of mat-building filamentous sulfur-oxidizing *Beggiatoaceae* lineages [8–13], and investigated the effects of high temperature and hydrothermally influenced geochemistry as local ecological constraints on microbial community structure in the underlying sediments [14, 15]. These sediments harbor complex anaerobic microbial communities, including diverse methanogens [16], thermophilic or thermotolerant archaea that oxidize methane and short-chain alkanes [15, 17–21] and hydrocarbon-oxidizing, free-living or syntrophic sulfate-reducing bacteria (reviewed by [22]). In metagenomic surveys of mat-covered hydrothermal sediments, oxygen- or nitrate-reducing, chemosynthetic Gamma- or Epsilonproteobacteria occur at the sediment surface whereas the sediment column is dominated by functionally and phylogenetically diverse uncultured, anaerobic bacteria and archaea [23, 24].

**Fig 1:**
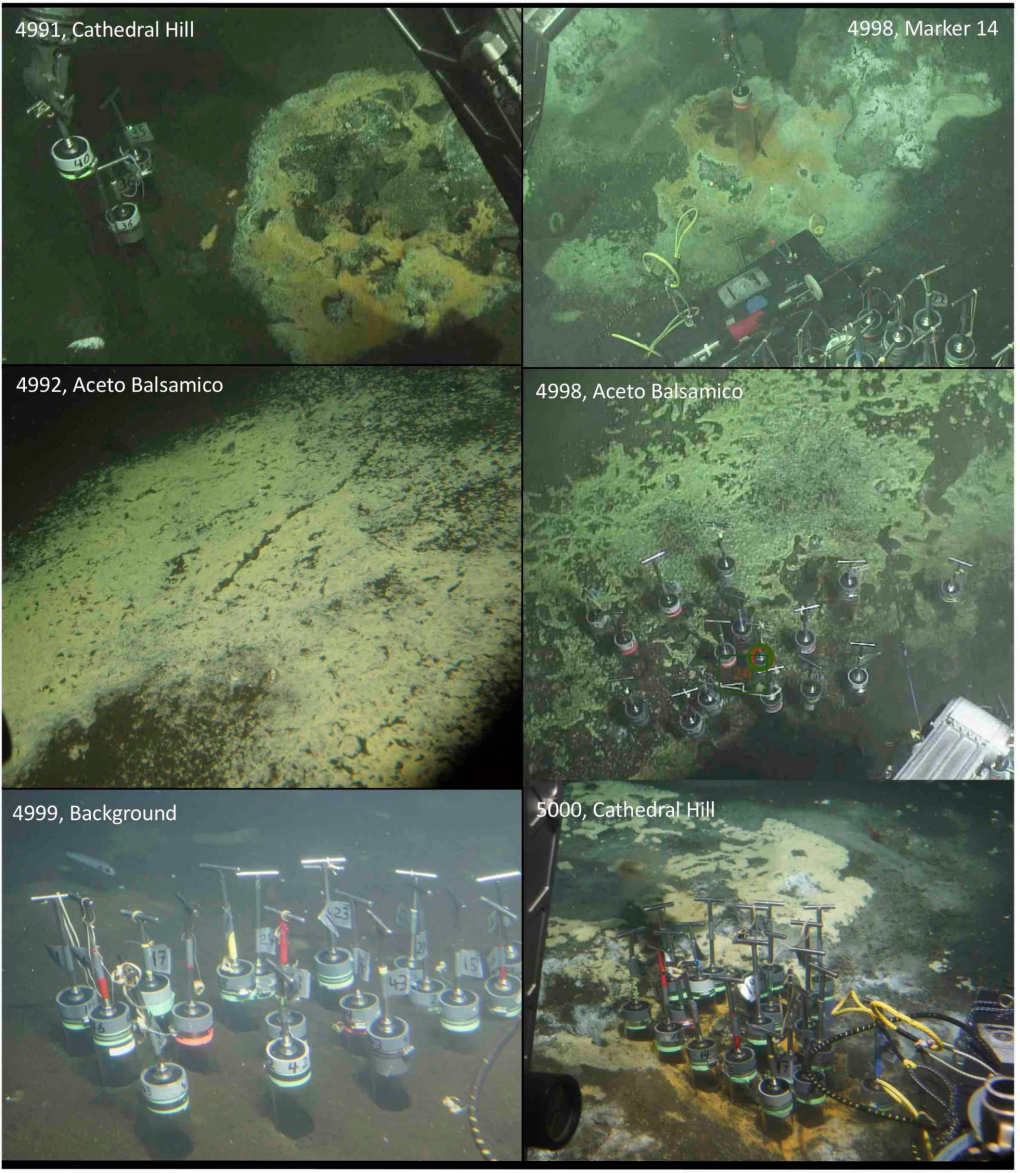
Sampling sites. In-situ photographs of sampling sites in the southern axial valley of Guaymas Basin. Courtesy of *Alvin* group, WHOI.

Despite the growing body of knowledge describing how the environment influences bacterial and archaeal community structuring in Guaymas Basin, little is known about the environmental controls on fungal diversity and distribution patterns. Fungal studies in Guaymas Basin sediments are so far limited to initial sequence-based [25, 26] and small-scale cultivation studies [27]. To explore environmental controls on fungal diversity and occurrence patterns, and fungal associations with bacteria and archaea, we analyzed site- and depth-related patterns of bacteria, archaea and fungi in hydrothermally influenced sediments of Guaymas Basin under different thermal and geochemical regimes retrieved from four different locations: i) hot hydrothermal sediments covered by orange-pigmented *Beggiatoaceae* mats in the Cathedral Hill area, ii) temperate sediments covered by yellow sulfur precipitates and filamentous sulfur oxidizers at the Aceto Balsamico location [2], iii) hot sediments with orange-pigmented mats surrounded by yellow and white mats at the Marker 14 location [14], and iv) bare background sediments with ambient seawater temperatures at a nearby control site (Fig 1, S1 Fig in S1 File). Previous investigations in Guaymas Basin have shown that sediment temperature affects *Beggiatoa* mat heterogeneity [9], limits methane and short-chain alkane oxidation and organic matter remineralization in hydrothermal sediments [14, 15] and cold seeps [28]; further, site-specific environmental histories can influence ecological structuring [29]. Overall, these studies suggest that bacterial and archaeal communities are predominantly structured by *in-situ* thermal and geochemical regimes.

To broaden our understanding of microbial processes in hydrothermally influenced sediments and to identify potential interactions between bacteria, archaea and fungi, we examined environmental controls on fungal communities in the context of parallel analyses of bacteria and archaea from the same sample set, keeping in mind that these controls are likely to change between surficial and subsurface sediments. Specifically, we investigated whether fungal communities in Guaymas Basin follow similar thermal and biogeochemical controls as bacteria and archaea, or are structured differently, perhaps stochastically or by co-occurrence with other microbiota.

## Material and methods

### Field survey and sampling

Guaymas Basin sites were visited and sampled with R/V *Atlantis*, HOV *Alvin* and AUV *Sentry* during cruise AT42-05 (Nov. 15–29, 2018). *Alvin* dives targeted previously explored sampling areas [2] that were mapped in additional detail by AUV Sentry. Photo coverage of *Alvin* dives is available at the *Alvin* frame-grabber site [http://4dgeo.whoi.edu]. The bathymetry of the hydrothermally active graben segment in southern Guaymas Basin had been mapped previously by AUV *Sentry* during dives 407–409 and 413–417 in 2016 (S1 Fig in S1 File) from a height of 65–70 meters above bottom [30]. Push cores of approx. 12” or 16” (30 to 40 cm) length were collected during *Alvin* dives 4991 to 5001, returned to the surface, and sampled in the shipboard laboratory within a few hours. Sampling site data are summarized in Table 1.

**Table 1:** *Alvin* cores. Metadata for sediment cores sampled for bacterial, archaeal and fungal community composition (B, A, F), and only fungal community composition (F). Temperatures are mid-point approximations for top, middle and bottom sediment layers in each core.

Dive & Core	Sampling area	Latitude/longitude	Depth in m	Mats	Slicing scheme in cm intervals	community composition	Approx. thermal range
4991–10	Cathedral Hill	27.0114/-111.4045	2014	Orange	0–6, 6–12, 12–18	B, A, F	16/33/45°C
4991–12							
4991–37	Cathedral Hill	27.0114/-111.4045	2014	Bare	0–10,10–20,20–30	F	5/8/10°C
4991–38							
4991–42	Cathedral Hill	27.0114/-111.4045	2014	White/orange	0–10,10–20,20–30	F	8/12/17°C
4991–43							
4992–22	Aceto Balsamico	27.0081/-111.4071	2012	Yellow	0–10, 10–20	B, A, F	6/9°C
4992–23							
4994–07	Cathedral Hill	27.0114/-111.4045	2014	Orange	0–7, 7–14, 14–21	F	8/14/18°C 3/8/14°C
4994–09					Surf., 0–7, 7–14		
4998–13	Marker 14	27.0079/-111.4072	2011	Orange	0–7, 7–14, 14–21	B, A, F	12/33/60°C
4998–18							
4998–19	Aceto Balsamico	27.0078/-111.4071	2011	Yellow	0–10,10–20,20–30	B, A, F	5/9/13°C
4998–21							
4999–17	Background	27.0069/-111.4066	2014	Bare	0–5, 5–10, 10–15	B, A, F	3/3/3°C
5000–11	Cathedral Hill	27.0122/-111.4039	2009	Orange	0–7, 7–14, 14–21	B, A, F	23-33/44-69/74-86°C
5000–20							

### Thermal profiles

Thermal profiles were measured in surficial sediments using *Alvin’s* 50 cm heat flow probe (https://ndsf.whoi.edu/alvin/using-alvin/sampling-equipment/). The 50 cm probe has thermal sensors every 10 cm, starting 5 cm under the attached plastic disk (the “puck”) that limits probe penetration and rests on the seafloor once the probe is inserted. After approx. 3 to 5 minutes, temperature readings stabilize and are recorded. The heat flow probe shorted at the beginning of *Alvin* dive 5000; instead, the thermosensor within the tip of the suction intake was inserted into the sediment at approx. 5 cm, 10 cm and 20 cm depth, and the temperature was recorded immediately. Thermal profiles adjacent to sediments used in this study are compiled in Table 2.

**Table 2:** Thermal profiles. *Alvin* temperature measurements with heatflow probe (Dives 4991, 4992, 4998, 4999) and one-point T-sensor (Dive 5000). Sediment depths for T-sensor measurements during Dive 5000 were estimated by the *Alvin* pilot.

Alvin Dive	4991			4992	4994	4998				4999	5000		
Temp. Profile				T3		T1	T2	T9	T10		T1	T2	T3
Mats	orange	none	white	yellow	orange	orange		yellow		none	orange		
Nearby cores	10 to 12	35 to 40	41 to 45	18 to 24	7, 9	11 to 18		19 to 27		14 to 24	8 to 23		
0 cm	3.8–5.0	3.4–5.4	3.3–4.4	3.0–3.5	n.d.	n.d.	n.d.	n.d.	n.d.	n.d	n.d.	n.d.	n.d.
5 cm	14.0	4.6	7.9	5.7	5	12.4	15.8	5.0	5.6	3.2	20.6	23	33
10 cm	28.9	6.7	9.3	7.3	10.4	33.3	32.8	7.3	8.0	n.d.	44	53	69
15 cm	56.4	8.0	11.6	9.2	n.d.	53.7	50.9	8.6	8.7	n.d.	n.d.	n.d.	n.d.
20 cm	48.9	9.0	14.0	11.0	n.d	74.1	69.3	10.7	11.0	n.d.	74	80	86
25 cm	55.8	9.9	17.2	12.7	26	92.2	88.2	12.5	12.4	n.d.	n.d.	n.d.	n.d.
30 cm	59.9	10.6	20.3	14.4	n.d.	110.4	107.6	14.8	14.7	n.d.	n.d.	n.d.	n.d.
35 cm	66.0	12.1	23.4	16.0	n.d.	>115	>115	17.3	17.0	n.d.	n.d.	n.d.	n.d.
40 cm	72.7	13.1	26.8	17.8	n.d.	>115	>115	19.5	19.5	n.d.	n.d.	n.d.	n.d.
45 cm	79.8	13.7	31.0	19.7	48	>115	>115	21.7	21.2	3.9	n.d.	n.d.	n.d.

### Porewater geochemistry

For porewater analysis, intact sediment cores were sampled using Rhizons (Rhizosphere Research Products, Wageningen, NL) as described previously [31]. The overlying water was removed from the cores and holes were drilled at designated sediment sampling horizons. Rhizons washed with hydrochloric acid (1M) and deionized water were injected through these holes, and vacuum was applied with syringes for approx. 30 min to collect porewater samples. The sediment interval depths are given in Table 1. For sulfide analysis, 1 ml of the collected porewater samples were fixed with 0.1 ml 0.1 M zinc acetate solution. Porewater samples were frozen at -20°C for storage and shipping.

At the Max-Planck-Institute for Marine Microbiology (Bremen, Germany), samples were homogenized and sulfide concentrations were determined photometrically using the methylene blue method in 2 ml assays [32]. For sulfate measurements, the same samples were diluted in water (1:50) and analyzed using ion chromatography (Metrohm 930 Compact IC flex with Metrosep A PCC HC/4.0 preconcentration column, and Metrosep A Supp 5 Guard/4.0 chromatography column). The porewater concentrations of ammonium, phosphate, nitrate/nitrite and silicate were determined using a continuous flow nutrient analyzer (QuAAtro39; Seal Analytical) as published previously [33]. These data are compiled in the (S1 Table in S1 File) and are also documented online [34].

As a complement to Rhizon sampling, additional geochemical analyses were performed on centrifuged porewaters and sediments from three layers per core (surface, middle, bottom) of 6–10 cm each (Table 1). Sediment cores were sampled in 3 cm intervals. Sediment samples of ca. 40 ml were centrifuged in 50 ml conical Falcon tubes under nitrogen for 5 to 10 minutes at 1000 g, resulting in the separation of 8 to 10 ml porewater from the sediment. For porewater sulfide analysis, 1 ml porewater subsamples were drawn into syringes, filtered immediately through 0.45 μm filters, and placed in Eppendorf sample vials each containing 0.1 ml of 0.1 M zinc acetate solution to preserve the sulfide as stable zinc sulfide precipitate until analyzed. Sulfide was quantified spectrophotometrically at UNC-Chapel Hill using the methylene blue method [32]. For sulfate analysis, 1 ml porewater samples were immediately acidified with 50 microliters of 1 N HCl and bubbled with N_2_ for 1 minute to remove hydrogen sulfide. After returning the samples to the home laboratory, sulfate concentrations were determined using the ion chromatograph of the UNC Environmental Program (S2 Table in S1 File). At Louisiana State University Wetland Biogeochemistry Analytical Services (WBAS), colorimetric determinations of ammonium, nitrate, soluble reactive phosphorus and total phosphorus were performed using a OI Analytical Flow Solutions IV auto analyzer, and sediment total nitrogen and carbon (weight %) were measured with a Costech 1040 CHNOS Elemental Combustion system (S3 Table in S1 File). Filtered porewater samples were analyzed for dissolved organic carbon (DOC) and total dissolved nitrogen (TDN) using a Shimadzu TOC/TN analyzer. Dissolved organic nitrogen (DON) was calculated by subtracting TDN from the sum of the inorganic nitrogen species (S3, S4 Tables in S1 File).

For methane measurements, sediment samples of 2 ml were collected from freshly recovered cores using cut-off syringes, and transferred into serum vials supplemented with 1 ml of 1M NaOH which were stoppered with thick blue butyl rubber stoppers and crimp-sealed. Methane δ^13^C isotope values from headspace gas samples were measured using a Finnigan-MAT DeltaPlus Stable Light Isotope Ratio Monitoring Mass Spectrometer in the Organic Mass Spectrometry Facility at WHOI. Values are reported in the per mil (‰) notation relative to Vienna Pee Dee Belemnite (VPDB) (S5 Table in S1 File).

### DNA extraction, amplification and sequencing

Freshly recovered sediment cores were divided into three layers (near-surface, middle, bottom) of 6 to 10 cm thickness each (Table 1) for DNA extraction and sequence-based analysis. This approach covered the available thermal range of cored sediment, while providing sufficient material for coordinated experiments with other cruise participants, and accommodating temperature fluctuations due to pulsating hydrothermal flows that impact surficial (5 to 10 cmbsf) sediments [14]. Total DNA was extracted using the FastDNA^TM^ Spin Kit for Soil (MP Biomedicals), as recommended for marine sediment [35], following the manufacturer’s protocol. Bacterial and archaeal SSU rRNA hypervariable region 4–5 (V4-V5) amplicons were generated using the 515F-Y (5′‐GTGYCAGCMGCCGCGGTAA-3’) and 926R (5′‐CCGYCAATTYMTTTRAGTTT-3’) primers and customized thermocycling parameters [36]. Fungal ITS2 region amplicons were generated using the 5.8S‐Fun (5′‐AACTTTYRRCAAYGGATCWCT‐3′) and ITS4‐Fun (5′‐AGCCTCCGCTTATTGATAT-GCTTAART‐3′) primers and customized thermocycling parameters [37]. All amplicons were generated and sequenced at Georgia Genomics and Bioinformatics Core, University of Georgia, using Illumina MiSeq PE 300 chemistry.

### Bioinformatics analyses

Bacterial, archaeal and fungal gene sequence analyses were performed using the DADA2 package [38] implemented directly in R and QIIME2, respectively. Briefly, for bacterial and archaeal 16S rRNA genes, forward and reverse reads were trimmed with the filterAndTrim() command using the following parameters: trimLeft = c(20,20), maxEE = c(2,2), rm.phix = TRUE, multithread = TRUE, minLen = 130, truncLen = c(290,200). Error assessments and independent forward and reverse read de-replication were performed. Sequencing errors were removed to better infer the composition of the samples using the dada() command and, subsequently, error-free forward and reverse reads were merged using the mergePairs() command, specifying overhand trimming and a minimum overlap of 150 base pairs. Bacterial and archaeal 16S rRNA gene Amplicon Sequence Variants (ASVs) were assigned taxonomy using the SILVA 132 database [39]. For fungal ITS rRNA and 18S rRNA gene analyses, DADA2 was implemented in QIIME2 [40]. Fungal ASVs were assigned taxonomy by BLAST against the UNITE (v.8.0 2018-11-18) classifier [41]. Taxonomy assignments and count tables were merged with metadata as S4 objects for ordination analyses (PCoA) and data visualization in R using the phyloseq [42]), vegan [43], and MASS [44] packages. Further information on methods and ASV numbers is documented in S2 Fig and S6 Table in S1 File.

### Taxonomy notes

The classification scheme as implemented in the SILVA 132 database [39] is currently changing. For example, genomics-based taxonomy revisions have raised the Epsilonproteobacteria to phylum status [Epsilonbacteraeota, [45]] and reorganized the Deltaproteobacteria into several phylum-level lineages [46]. Since the taxonomic landscape continues to evolve, we use designations that are currently widely shared, in particular phylum-level archaeal designations that have gained widespread usage during the ongoing rapid expansion of the archaeal domain [for example Bathyarchaeota, [47, 48]]. Roadmaps for achieving consistent taxonomy of the cultured and uncultured microbial world have been proposed recently [49].

### Network analyses

To visualize the relationships between archaeal, bacterial and fungal ASVs within the targeted sediment samples, network analyses were performed using Calypso [50] and MetagenoNets [51] web-tools. The 23591 ASVs were Hellinger-transformed, filtered to 84 ASVs using a minimum prevalence of 0.03 and an occurrence of 5, and subjected to network analysis for identification of co-occurring and mutually exclusive taxa using the CCREPE (Compositionality Corrected by Renormalization and Permutation) algorithm [51, 52] Taxa are represented as nodes, taxa abundance is shown as node size, and significant edges, based on Pearson’s critical r correlations, represent positive (blue) and negative (red) associations that we utilize for assisting with data interpretation and hypothesis generation. Complementary heatmaps were also processed using MetagenoNets.

## Results

### Guaymas Basin survey sites

During expedition AT42–05 to Guaymas Basin (Nov. 15–29, 2018), AUV *Sentry* re-surveyed the bathymetry of the southern Guaymas Basin trough [30] and submersible *Alvin* sampled hydrothermally active sediments. Different types of mat-covered sediments (Table 1) and thermal regimes (Table 2) were sampled at the Cathedral Hill, Aceto Balsamico, Marker 14 and Background locations.

**Fig 2:**
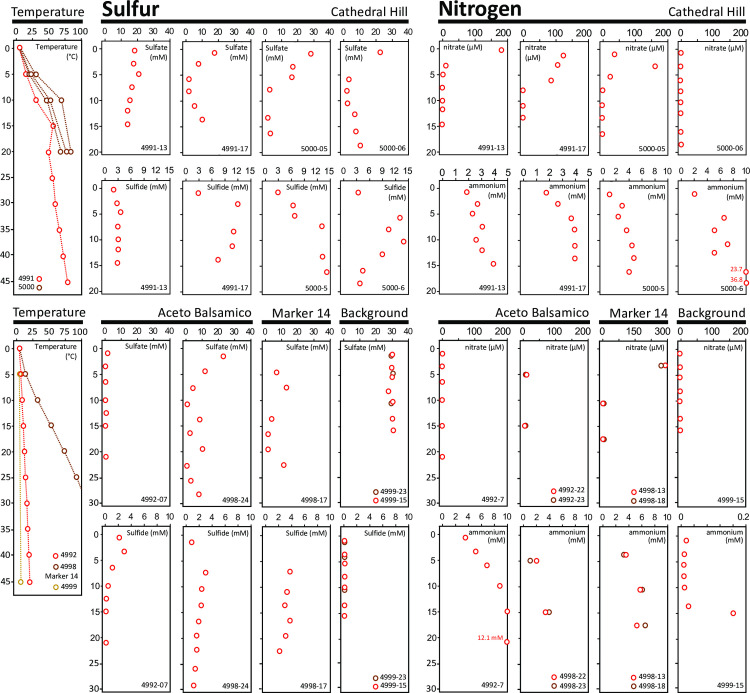
Thermal and porewater geochemical profiles of Guaymas Basin sediments. Most porewater samples were obtained by Rhizon sampling, and some ammonium and nitrate data were obtained by centrifugation (cores 4992–22,23 and 4998–13,18). Rhizon-based porewater data are tabulated in S1 Table in S1 File, and centrifugation-based data in S2 and S3 Tables in S1 File.

At Cathedral Hill, gradually sloping sediment-covered mounds with extensive hydrothermal areas and white, yellow and orange microbial mats (Fig 1) are topped with large hydrothermal edifices [2]. The Cathedral Hill area was targeted for push-core sampling of high-temperature microbial mats by submersible *Alvin*. These hydrothermal sediments show rapidly increasing temperatures with depth that reach up to 75 to 100°C within 30 to 40 cm from the sediment-water interface (Fig 2). Push cores from hydrothermal sediments with orange mats of filamentous *Beggiatoaceae* are characterized by sulfate replenishment below the sediment surface (Fig 2), most likely by seawater inmixing and active hydrothermal circulation [2, 14]. Interestingly, nitrate in concentrations of 100 to 200 μM appears in surficial sediment of several cores, a possible consequence of entraining seawater and nitrate-accumulating filamentous *Beggiatoaceae* [13]. Consistent with previous analyses of Guaymas Basin hydrothermal fluids [3] and sediments [13], the hydrothermal Cathedral Hill sediments share high ammonium concentrations in the range of 2 to at least 6 mM (Fig 2, S1 Table in S1 File). Porewater methane δ^13^C-CH_4_ values for Cathedral Hill sediments were generally in the range between -40 and -25 ‰ (VPDB) (S5 Table in S1 File), similar to previously studied methane-rich hydrothermal sediments in Guaymas Basin [14]. Core 4991–35, from cool sediment adjacent to a hydrothermal hot spot (Table 2), differs from hot Cathedral Hill cores by its relatively δ^13^C-depleted methane (-45 ‰) (S5 Table in S1 File).

The Aceto Balsamico area is located ca. 200 meters south of Cathedral Hill [2]; here, moderately warm sediments are covered with lime-yellow mats of sulfur deposits and sulfur-oxidizing bacteria (Fig 1). The thermal gradient reaches ca. 25 to 30°C at 40 cm depth (Fig 2). The name for this mat area is derived from the near-millimolar porewater acetate concentrations recorded within approximately 30 cm sediment depth, more than an order of magnitude higher than the moderate acetate concentrations (10–20 μM) observed at other mat-covered sediments of Guaymas Basin [2]. Sediments at this location are sulfate-depleted, sulfidic and rich in ammonia, best illustrated by cores from Dive 4992 (Fig 2), and the sediment-water interface shows local accumulation of elemental sulfur or polysulfides [2]. Porewater methane δ^13^C-CH_4_ values for the temperate Aceto Balsamico sediments were generally between -47 and -55 ‰ (VPDB) (S5 Table in S1 File), clearly more depleted in δ^13^C-CH_4_ than other hydrothermal sediments in Guaymas Basin [14].

Nearby, the Marker 14 area (named for the site marker placed there in 2009, and still present in 2018) contains hotter sediments where extensive yellow mats transition into localized spots of orange-colored filamentous *Beggiatoaceae* (Fig 1) that are indicative of strong hydrothermal circulation [9]. Hydrothermal sediments from this area have been studied extensively by 16S rRNA gene sequencing and metagenomic analyses to demonstrate that prokaryotic microbial community composition and function are primarily controlled by hydrothermal and geochemical gradients [14, 23]. The Marker 14 sediments were sampled in the anticipation that they provide a geochemical and microbial intermediate between classic Guaymas Basin hydrothermal sediments (orange mats, sulfate inmixing, surficial nitrate peaks, steep temperature gradients) and Aceto Balsamico sediments (yellow precipitates, moderate temperatures, no sulfate inmixing, no nitrate). High ammonium concentrations of 2–10 mM are shared by all three categories of hydrothermal sediment (Fig 2; S1, S3 Tables in S1 File). The δ^13^C-CH_4_ values of Marker 14 sediments (S5 Table in S1 File) matched those of previously studied sediment cores from this area [9, 14]. Interestingly, some thermal gradients at Marker 14 (measured during *Alvin* Dive 4998) were the steepest encountered during the entire expedition and could not be fully recorded as the thermal sensors of the heat flow probe reached their limit (>115°C) within 30 cm depth (Fig 2). As found previously in Guaymas Basin, hydrothermal activity patterns and thermal gradients are highly localized and vary over short distances [14].

In olive-brown background sediments lacking microbial mats (Fig 1), sulfate persisted at seawater concentrations, sulfide was not detectable, and nitrate remained in the range of a few micromolar to below detection. Ammonium accumulated to only moderate concentrations (160 μM) at depth. The thermal gradient was strongly attenuated (3.2°C at 5 cm depth, 3.8°C at 45 cm depth), porewater sulfide was not detectable, and porewater sulfate remained near seawater concentration throughout the core (Fig 2, S1, S3 Tables in S1 File). Strictly speaking, even a thermal gradient of 0.6°C per 40 cm or 1.5°C/m is evidence for residual hydrothermal activity that pervades the entire hydrothermal area in the southern spreading center of Guaymas Basin [2]. Therefore, these sediments are “background” only in comparison to more active sites. Interestingly, they contain residual methane with highly δ^13^C-depleted δ^13^C-CH_4_ values, approximately -65 to -70 ‰ (S5 Table in S1 File), similar to residual methane from cold “background” sediments analyzed previously [14].

Analyses of centrifuged porewater and sediment cakes illustrate the geochemical differences between hydrothermal cores and background, and with sediment depth, on a core-by-core basis (S3 Table in S1 File), and also when multiple cores from particular sampling areas—Cathedral Hill, Aceto Balsamico and Marker 14—are averaged (S4 Table in S1 File). Averaged ammonium concentrations between 2 to 6 millimolar in these hydrothermal cores contrast with 0.12 mM in background sediment (S4 Table in S1 File). Averaged DIC concentrations for cores from Cathedral Hill (62 mg/L), Aceto Balsamico (101 mg/L) and Marker 14 (72 mg/L) exceed those of background sediment (31 mg/L) and cold Cathedral Hill cores (26 mg/L) by factor two or three (S4 Table in S1 File). Porewater DOC and DON concentrations averaged separately for Cathedral Hill (94 and 58 mg/L), Aceto Balsamico (117 and 84 mg/L), and Marker 14 hydrothermal sediments (181 and 107 mg/L) are quite similar to each other (S4 Table in S1 File). In cold background sediment, DOC concentrations remain similar to hydrothermal sediments (90 mg/L), but DON concentrations are almost two orders of magnitude lower (3 mg/L). Two cold cores collected outside of a Cathedral Hill mat (4991–37 and 38) also show a wide difference between DOC (average 39 mM) and DON (average 1.2 mM) porewater concentrations (S4 Table in S1 File). For Cathedral Hill, Aceto Balsamico and Marker 14 hydrothermal sediment, DOC and DON concentrations show contrasting depth trends; DOC decreases with depth whereas DON increases (or shows no visible trend, in Cathedral Hill) (S4 Table in S1 File). In all sampling locations except the background site, TOC and TON content decrease with sediment depth (S4 Table in S1 File). These trends are consistent with geochemical observations and experimental evidence that hydrothermal activity mobilizes biomass and organic carbon from deeper sediment layers towards the sediment/water interface [5].

### Phylum- and class-level community composition

Bacterial and archaeal community composition at the Domain, Phylum and Class levels highlight differences within and between sampling sites (S3-S5 Figs in S1 File). All microbial community analyses in this study have to be qualified by the fact that they are based on sequence frequencies, which are derived from the microbial community but do not necessarily represent it in identical proportions due to potential taxonomic biases in recovery of nucleic acids and amplification of marker genes, as well as variations in gene copy numbers.

Archaeal signatures increase proportionally with increasing temperature, from approximately 10% in cold background sediment to around 20% in the temperate Aceto Balsamico sites. In the hot Marker 14 and Cathedral Hill sediments, archaeal ASVs increase with depth from 50 to 80% of recovered prokaryotic ASVs (S3 Fig in S1 File). At the phylum- and class-level composition (S4, S5 Figs in S1 File), the Gamma- and Deltaproteobacteria, Planctomycetes and Chloroflexi that predominate in the background sediment yield to predominantly Atribacteria of the JS1 lineage [53], Deltaproteobacteria, and Methanomicrobia in the Aceto Balsamico sediments, with epsilonproteobacterial Campylobacteria as a fourth major group in surficial sediments. At Marker 14 sites, the surficial sediment communities resemble the bacterially dominated Aceto Balsamico community, but the proportions of Bathyarcheota and Thermoplasmata increase downcore. The Cathedral Hill sites show a larger degree of site-specific variability, but harbor presumably thermophilic Acetothermiia [54], consistently in all samples, and show downcore increasing proportions of Thermoplasmata, Crenarchaeota and Bathyarchaeota (S4, S5 Figs in S1 File).

### Principal coordinate analyses of bacterial and archaeal communities

**Fig 3:**
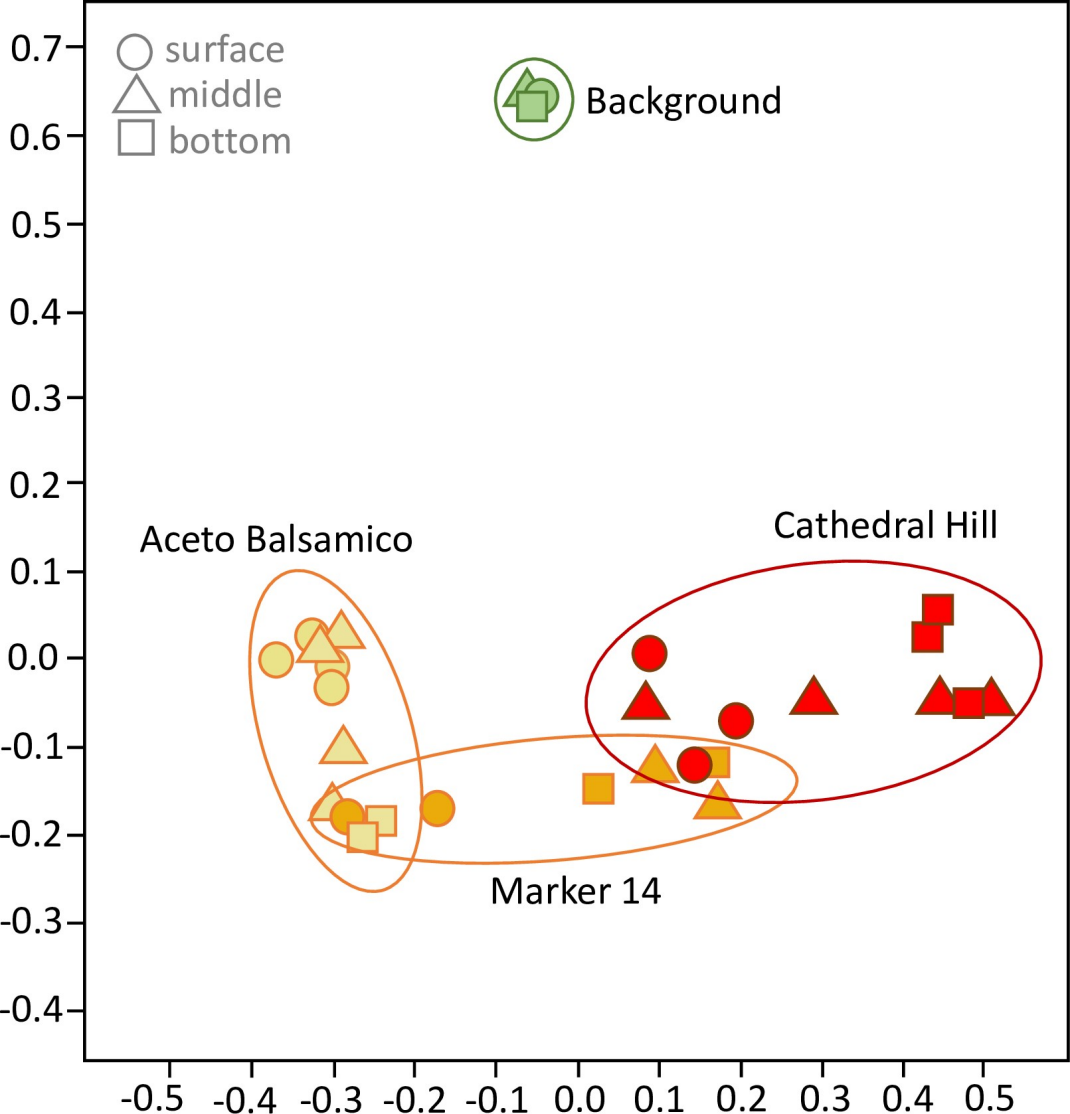
Cluster analysis of bacterial and archaeal populations. Principal Coordinate Analysis of Bacterial and Archaeal communities in Guaymas Basin sediments, color- and symbol-coded by site (Cathedral Hill, Aceto Balsamico, Marker 14, and Background) and by core position (surface, middle, and bottom sediment). The horizontal and vertical axis account for 23.4% and 17% of the dataset variance, respectively. A fully annotated version with individual sample labels is available as S6 Fig in S1 File.

Phylogenetic analyses indicate site-specific differences in bacterial and archaeal community composition. To test this possibility more rigorously, Principal Coordinate Analysis was performed on the complete bacterial and archaeal sequence dataset, and indeed this analysis separated the bacterial and archaeal populations according to sample collection area (Fig 3). The tightly clustered Background samples are separated from all other sites. The Aceto Balsamico samples are separated from Cathedral Hill samples, and the Marker 14 samples are connecting these two hydrothermal sample sets. Specifically, Marker 14 shallow (0–10 cm) and deep (10–20 cm) samples are associated with deep (10–20 and 20–30 cm) Aceto Balsamico and shallow (0–10 cm) Cathedral Hill samples, respectively (S6 Fig in S1 File). When the background samples are omitted from the analysis, the Aceto Balsamico and Cathedral Hill samples remain separated, with Marker 14 samples intermediate between them (S7 Fig in S1 File). Notably, site-specific clustering is also observed when the ordination analysis is performed with only Archaea or only Bacteria (S8 Fig in S1 File), indicating that bacterial and archaeal communities follow similar structuring patterns independently. Phylogenetic analyses and balloon plots of methane-cycling archaea (S9 and S10 Figs in S1 File) and sulfate-reducing bacteria (S11 and S12 Figs in S1 File) demonstrate site-specific occurrence patterns also at the level of genus- or family-level lineages (S13 Fig in S1 File, and S1 Text in S1 File).

### ASV frequency patterns

**Fig 4:**
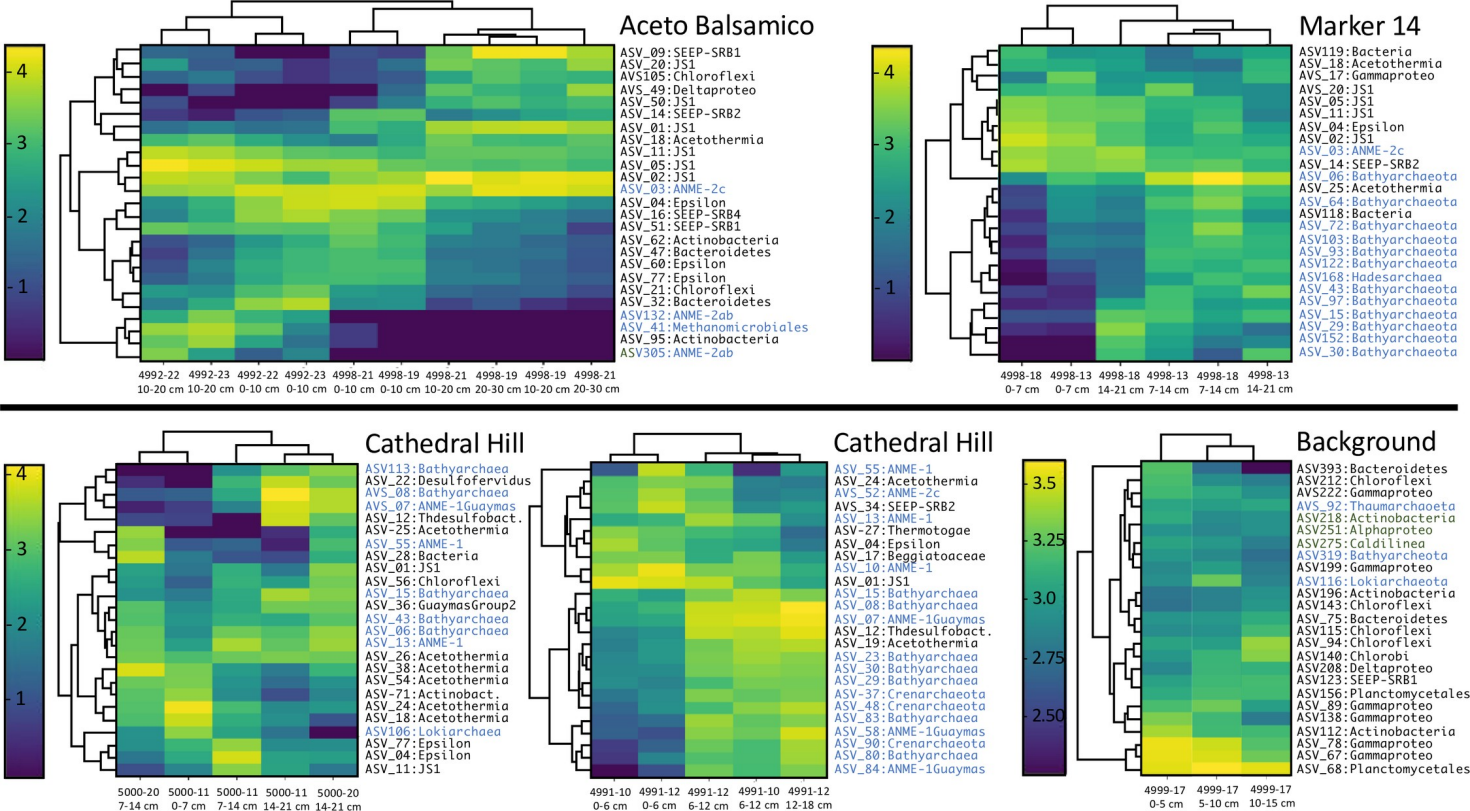
Bacterial and archaeal ASV heatmap in Guaymas Basin sediment samples. Scale bars showing log-scale ASV frequencies extend from less frequent ASVs in dark blue to frequent ASVs in lime green. Frequency scales are adjusted to each sampling location. Branching patterns on the left of each heatmap show groupings of ASVs that occur with similar frequency across the sample set; branching patterns on top of each heatmap group sediment samples by shared ASV frequency patterns.

Adding taxon specificity to phylum- and class-level community patterns, heatmaps of ASV occurrences show site- and depth-specific distribution patterns of the most frequently detected bacterial and archaeal ASVs (Fig 4). In the cold background sediment, the 25 most frequently occurring ASVs are primarily assigned to Gamma- and Deltaproteobacteria, Planctomycetes and Chloroflexi; archaeal ASVs are limited to three representatives of the Thaumarchaeota, Bathyarchaeota and Lokiarchaeota (Fig 4). In the temperate Aceto Balsamico cores, epsilonproteobacterial ASVs appear in the surface sediment and Atribacteria (JS1) ASVs occur throughout all samples. Different deltaproteobacterial ASVs show distinct depth preferences: ASV16 for the surface sediment and ASV09, 14 and 49 for deeper sediments depths (Fig 4). Three of the four archaeal ASVs (ANME-2ab and Methanomicrobiales) appear preferentially in surface layers, and one ASV (ANME-2c) in deeper samples. Similar to Aceto Balsamico, the surface layers at Marker 14 harbor mostly atribacterial ASVs and representatives of the Gamma-, Delta- and Epsilonproteobacteria, but archaeal ASVs (mostly Bathyarchaeota) appear prominently below the surface sediment and distinguish the ASV patterns of the deeper, warmer sediments (Fig 4). The consistent depth patterns shown by bacterial and archaeal ASVs in the Aceto Balsamico and Marker 14 sites differ from the core-to-core variability observed in the hot Cathedral Hill sites. Yet, several bathyarchaeotal and ANME-1 ASVs (in cores from *Alvin* dive 5000) and bathyarchaeotal, ANME-1 and Crenarchaeotal ASVs (in cores from *Alvin* dive 4991) show a preference for deeper and warmer sediment layers in Cathedral Hill samples (Fig 4).

### Fungal populations

**Fig 5:**
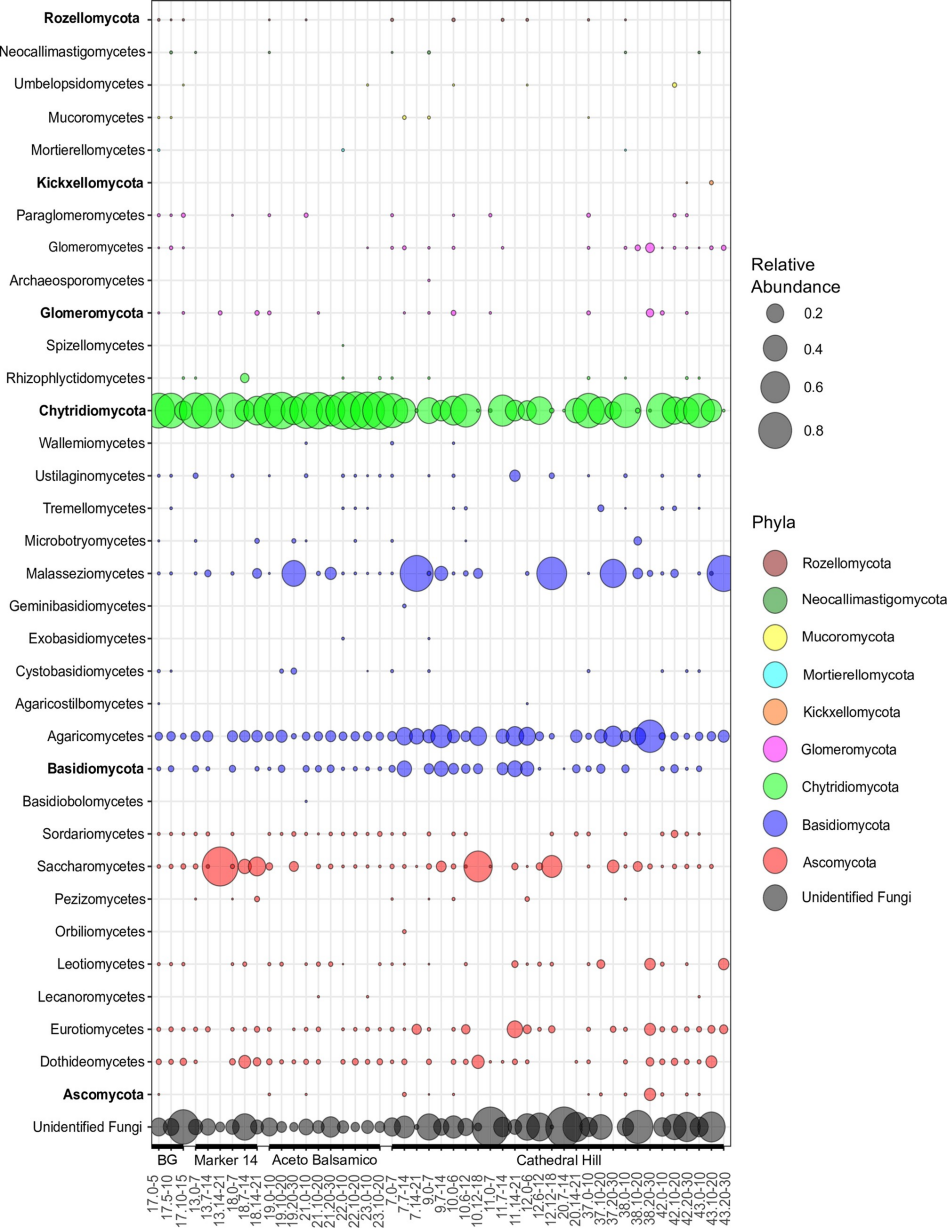
Fungal occurrence patterns in Guaymas sediments. Bubbles are color coded by phylum. Fungal sequences were assigned to class level when possible. Relative phylum abundance is shown in bold. BG stands for background.

A total of 2,653 fungal intergenic spacer sequences with a mean length of ~400bp were recovered from the Guaymas sediment libraries, and the resulting fungal ASVs affiliated with *Ascomycota* (phylum), *Basidiomycota* (phylum), *Glomeromycota* (phylum), *Chytridiomycota* (phylum), *Kickxellomycota*, (subphylum of Zoopagomycota), *Mortierellomycota* (subphylum of Mucoromycota), *Mucoromycota* (phylum), *Neocallimastigomycota* (phylum), *Rozellomycota* (subphylum of Ophistosporidia, previously named Cryptomycota) and unidentified fungi (Fig 5), based on recently established phylogeny and taxonomy [55]. In fungal ASV frequencies across the sample set, particular taxa are not linked with specific sample areas: *Chytridiomycota* and *Agaricomycetes* were widely distributed across the sample set, whereas *Malasseziomycetes* and *Saccharomycetes* showed relative abundance peaks in individual samples but not linked to a particular sampling area (Fig 5). Approximately one third of fungal iTags from Guaymas sediments remained unclassified, and one quarter of all fungal ASVs belong to “unidentified” fungi (26%). Unclassified fungal taxa are frequently recovered in different mycobiomes including sediments [56]. These “unidentified” fungal populations are particularly conspicuous in Cathedral Hill samples, where they show locally increased abundance (Fig 5).

Unexpectedly, zoosporic fungi of the *Chytridiomycota* predominate in Guaymas Basin sediments and account for 54% of all fungal ASVs in these sediment samples. Their abundance in Guaymas Basin contrasts with their frequent absence from previously analyzed deep-sea sediments [57, 58], hydrothermal deep-sea sediments [59] and deep bedrock ground water [60]. Only a few studies have detected this basal fungal lineage in deep-sea hydrothermal vents [61], deep-sea sediments [62], and submarine canyons [63], albeit at low proportions compared to other fungal phyla (from 0.8 to 4% of the total reads). As another basal fungal lineage, members of the *Rozellidea* subphylum have been detected in methane cold seeps in relatively high proportion with approximately one third of the total OTUs generated using cloning and sequencing [64]. Here, out of 302 *Chytridiomycota* ASVs recovered from these Guaymas sediments, order-level identifications were limited to 9 ASVs affiliated with the *Rhizophydiales*, and single ASVs assigned to the sister orders *Spizellomycetales* and *Rhizophlyctidales*, whereas the remaining chytridiomycotal ASVs remained taxonomically unresolved.

The *Ascomycota* account for 7% of all fungal ASVs, and fall predominantly into the *Saccharomycetes*, *Dothideomycetes* and *Eurotiomycetes*, which account for 64, 15, and 11.5% of all Ascomycota sequences in the Guaymas samples, respectively. The *Basidiomycota* account for 11% of all fungal ASVs, predominantly *Agaricomycetes* and *Malasseziomycetes* (49 and 30% of *Basidiomycota*, respectively). In some deeper samples, the *Agaricomycetes*, *Malasseziomycetes*, *Saccharomycetes* or fungi of unknown affiliation take the place of the otherwise omnipresent chytrids (Fig 5). Such a pattern contrasts with the frequent dominance of *Ascomycota* and *Basidiomycota* in the marine environment [65, 66], and shows that hydrothermal sediments of the Guaymas Basin represent an untapped reservoir of fungal diversity.

### Environmental parameters shaping the fungal community composition

**Fig 6:**
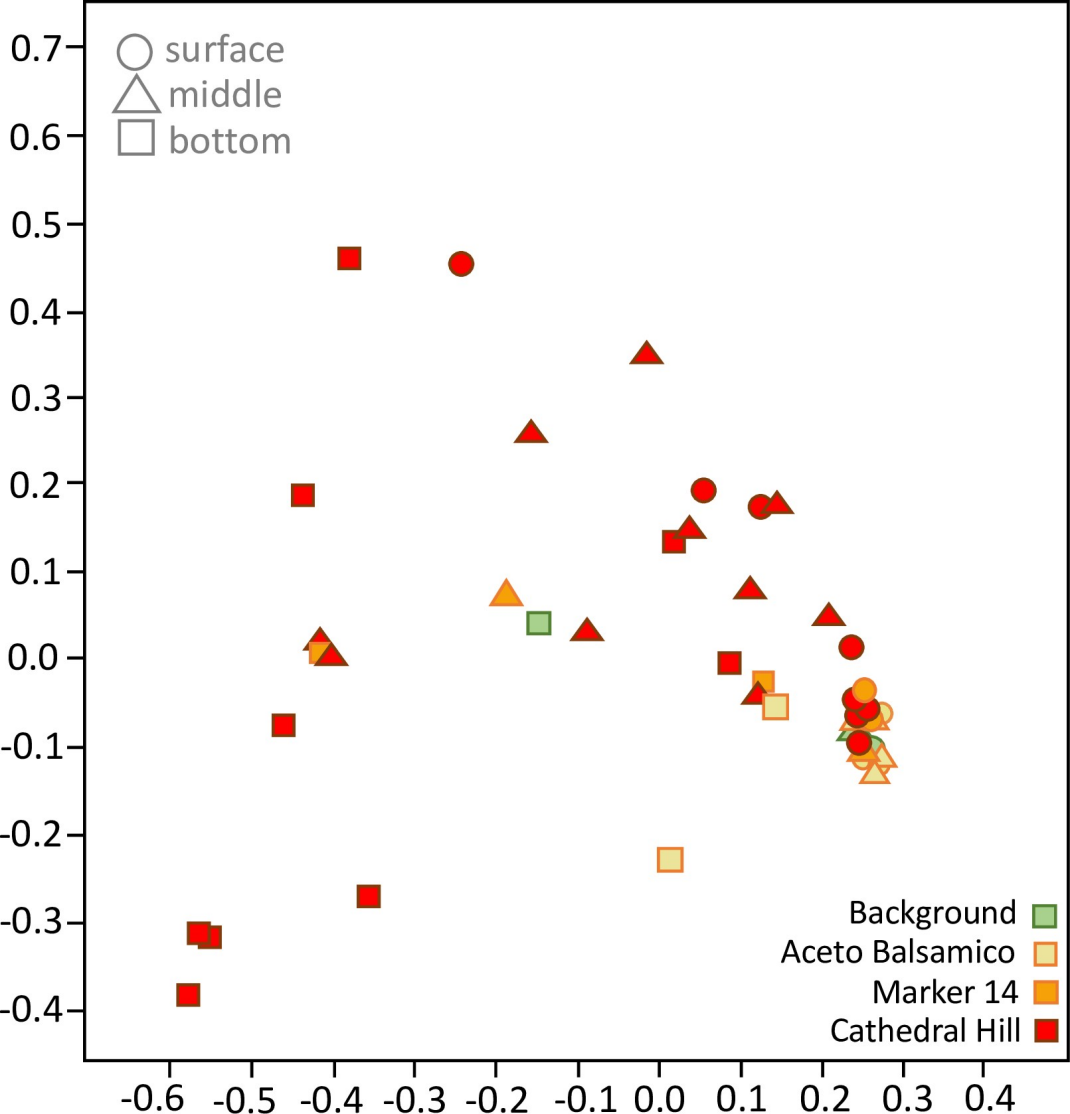
Cluster analysis of fungal populations. PCoA analysis of fungal communities based on fungal iTag ASVs in Guaymas Basin samples, color- and symbol-coded by sampling area (Cathedral Hill, Aceto Balsamico, Marker 14, and Background) and by depth (surface, middle, and bottom sediment). The horizontal and vertical axis account for 28.1% and 10.4% of the dataset variance, respectively. A fully annotated version of this figure with individual sample labels is available as S14 Fig in S1 File.

The phylogenetic bubble plot profile (Fig 5) indicates that the fungi do not show taxon-specific preferences for any of the hydrothermal sampling areas or our control site, in stark contrast to the pattern observed for bacteria and archaea (S10 and S12 Figs in S1 File). To examine this observation more rigorously, PCoA analysis was performed on the complete fungal dataset of intergenic spacer sequences. This analysis confirmed the lack of clustering by sampling site (Fig 6, S14 Fig in S1 File), but revealed a contrasting pattern of tightly clustered surficial samples from all sampling sites (background, Aceto Balsamico, Marker 14 and Cathedral Hill) with positive axis 1 values (Fig 6). Negative axis 1 values and the full range of axis 2 contained a broad spread of deeper (and a few shallow) sediment layers from hot hydrothermal Cathedral Hill sites where fungal communities appear to be distinct. This pattern indicates that the fungal communities of hotter hydrothermal subsurface sediments are different from each other and from those of the remaining samples, while the surficial fungal communities of different sampling sites, with potentially a single outlier, are generally similar to each other (Fig 6). Given the dynamic nature of hydrothermal sediments, the changing temperatures and chemical compositions of hydrothermal fluids, and their ephemeral flow paths, it is not surprising that at the very active and dynamic Cathedral Hill site, fungal communities exhibit the highest observed degree of variation (Fig 6).

Complementary analyses of the fungal α-diversity depending on several environmental parameters (site, temperature, sediment depth, type of mats) show that while Shannon indices did not reveal any significant differences, measurements of richness and evenness identified significant differences for temperature and sediment depth (S15 Fig in S1 File). Higher richness and lower evenness values were obtained for shallow sediment samples and thus lower temperatures, clearly indicating that a combination of higher fungal diversity and uneven proportions of different fungal taxa characterizes shallow sediment samples.

### Co-occurrence network analysis of microbial interactions

**Fig 7:**
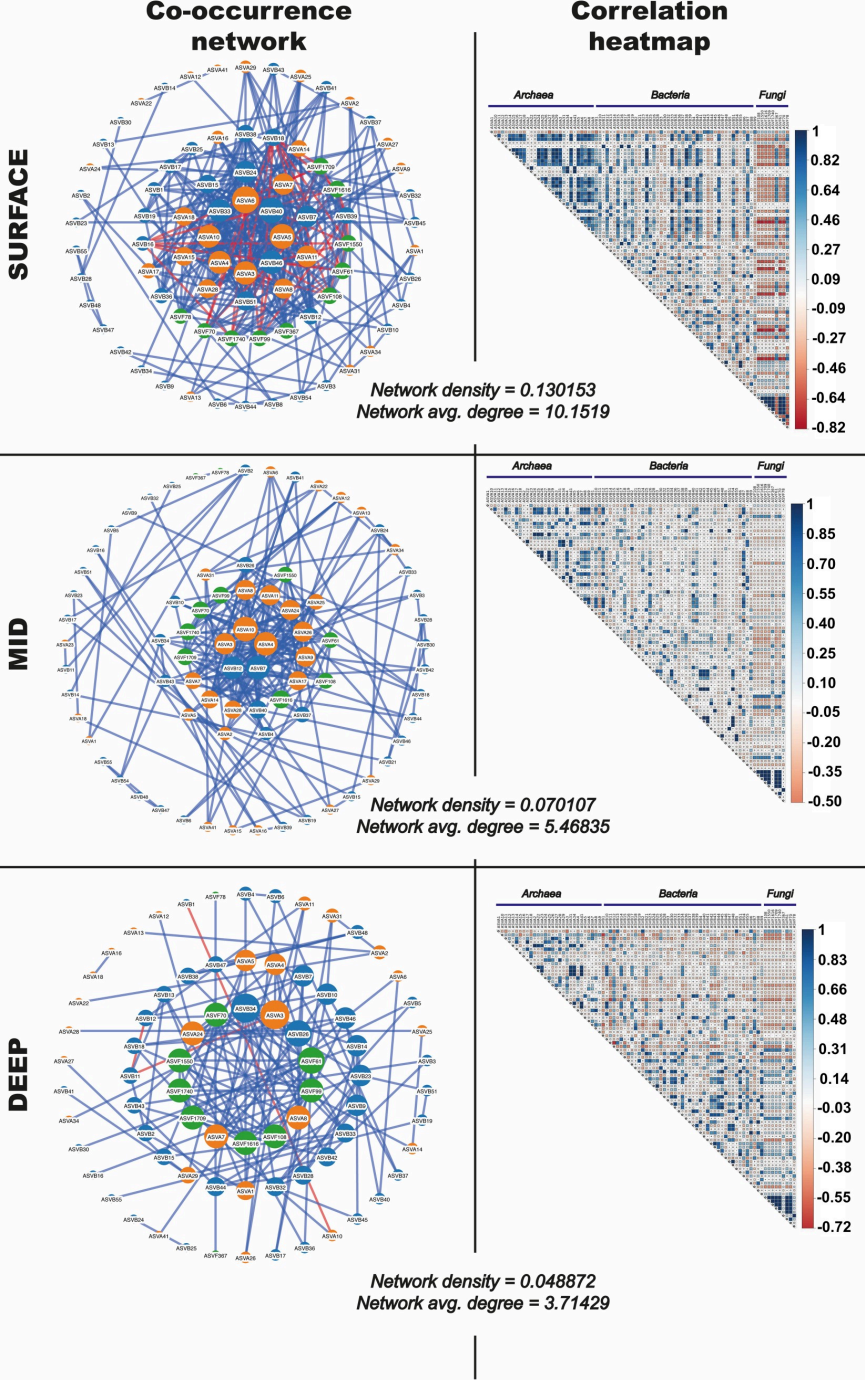
Interdomain network correlations in the Guaymas hydrothermal sediments depending on sampling depth. Nodes represent microbial taxa (orange for archaeal ASVs, blue for bacterial ASVs and green for fungal ASVs) and lines connect taxa whose abundances were significantly correlated. Nodes are sized depending on degree of interconnectedness. Blue lines indicate positive correlations and red lines indicate negative correlations.

**Fig 8:**
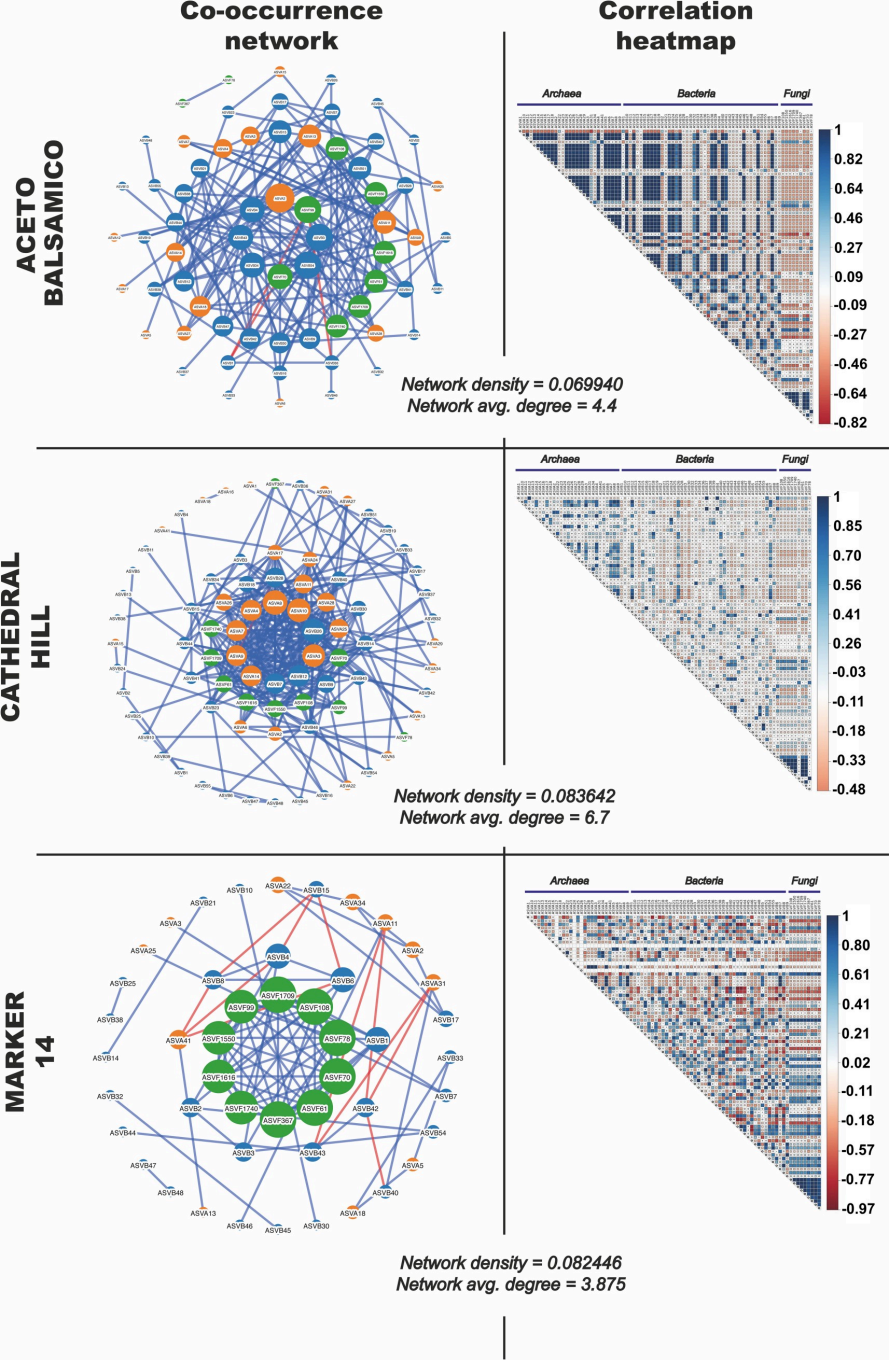
Interdomain network correlations in the Guaymas hydrothermal sediments depending on sampling site. Nodes represent microbial taxa (orange for archaeal ASVs, blue for bacterial ASVs and green for fungal ASVs) and lines connect taxa whose abundances were significantly correlated. Nodes are sized depending on degree of interconnectedness. Blue lines indicate positive correlations and red lines indicate negative correlations.

In Guaymas Basin, varying environmental and geochemical conditions generate an inherently complex hydrothermal sediment microbiome. To identify microbial interactions within and among the archaeal, bacterial and fungal communities in this habitat, the occurrence and abundance profiles of 100 dominant ASVs from these groups were mined for positive and negative ASV-specific co-occurrence interactions (visualized as networks) and correlations (visualized as heatmaps) depending on sample depth (Fig 7) and by sampling sites (Fig 8). With increasing depth, microbial interactions are attenuated, as shown by decreasing network density and average degree values that decrease from 0.130 to 0.049, and from 10.15 to 3.71, respectively. In surface samples, archaeal and bacterial ASVs correlated positively within and between domains, but neither correlated with fungal ASVs (Fig 7). Examination of several network metrics revealed no significant differences for node degree, betweenness and coreness, while eccentricity shows higher values for surface samples compared to intermediate and deep samples (S16 Fig in S1 File). As higher eccentricity assumes higher node proximity, surface ASVs appear more correlated with each other, strongly suggesting more complex interactions between surface ASVs compared to deeper ones.

Different hydrothermal sites strongly re-structure microbial interactions, as revealed by obvious differences in terms of correlations, either positive or negative (Fig 8). While network density and average degree values appear similar between sites, Aceto Balsamico produced a higher number of significant correlations. Specifically, more numerous positive correlations were found between different archaeal ASVs, between archaeal and bacteria ASVs, and within fungal ASVs, but negative correlations dominated between fungal ASVs and bacterial or archaeal ASVs (Fig 8). In the Cathedral Hill hydrothermal samples, the positive correlations between archaeal ASVs and between fungal ASVs persisted, but within a general pattern of increasingly patchy correlations relative to the Aceto Balsamico site (Fig 8). At site Marker 14, a complex patchwork of positive and negative interactions between bacterial and archaeal ASVs erased any domain-based pattern, except for fungal ASVs that remained correlated to each other. Analysis of background samples did not provide any significant correlations. The lower temperatures at Aceto Balsamico, compared to higher temperature ranges at Cathedral Hill and Marker 14, may have favored higher ASV correlations at Aceto Balsamico, and obscured them at the hotter sites. In terms of network metrics (S16 Fig in S1 File), node degree and betweenness did not show site-specific differences, but, generally, higher coreness values were observed for Cathedral Hill and higher eccentricity values were noted for Cathedral Hill and Aceto Balsamico.

## Discussion

The bacterial and archaeal communities of Guaymas Basin sediments are highly structured according to site-specific geochemical and thermal conditions, as shown by site-specific PCoA clustering (Fig 3), site-specific ASV distribution patterns (Fig 4), downcore decreasing alpha diversity (S2 Fig in S1 File), downcore increasing archaeal contribution (S4 Fig in S1 File), and site-specific phylum- and class-level changes in microbial community composition (S4, S5 Figs in S1 File). These trends are broadly consistent with observations that phylogenetic profiles and population proportions for bacteria and archaea in Guaymas Basin sediments change along hydrothermal gradients [14, 67], and they complement previous observations of divergent hydrothermal and seep communities in Guaymas Basin and the nearby Sonora Margin [28].

Fungal communities reflect different environmental controls. The dominance of chytrid phylotypes throughout surficial sediment samples (Fig 5) is best explained as a consequence of sedimentary input from the highly productive overlying water column. Chytrids include known phytoplankton parasites, especially of diatoms, chrysophytes and dinoflagellates, and follow the abundance of their hosts [68–71]. Seasonal chytrid blooms in coastal sea ecosystems corresponded to diatom blooms through multi-year time series analyses [72]. Chytrids are known to occur in diverse marine habitats [73–76] where their production of motile zoospores promotes colonization of particulate organic matter such as chitin-rich substrates [77]. In coastal sediments chytrid occurrence was positively correlated with the presence of dissolved silicate, an essential nutrient for a diatom-dominated phytoplankton community [78].

Via their parasitic and saprophytic activities, chytrids impact pools and sinking fluxes of dissolved organic carbon and overall nutrient dynamics in freshwaters, termed the “mycoloop” [79]. We propose the working hypothesis that the widespread chytrid sequences in Guaymas Basin sediments are derived primarily from sedimentation of the spring diatom bloom in this highly productive basin, with terrigenous input as a potential secondary source [80, 81]. After their arrival on the seafloor, chytrids can sustain themselves by degrading abundant phytoplankton biomass [17], and refractory detritus such as chitin [82]. In previous cases where chytrids were found at deep-sea hydrothermal sites, interactions with endemic fauna were suggested [61, 83], but their marked presence in every surficial sediment sample including the background sediment (Fig 5) indicates that their occurrence in Guaymas Basin is not linked to hydrothermal conditions or hydrothermal fauna. By contrast, *Saccharomycetes* and *Malassseziomycetes* enrichment below 10 or 20 cm depth (Fig 5) is likely favored by hydrothermal conditions selecting these potentially more thermotolerant taxa over the otherwise omnipresent chytrids.

In this interpretation, fungal populations in Guaymas Basin surficial sediments arise from regional sedimentation that ubiquitously imparts a shared chytrid overprint independent of local hydrothermal conditions. Subsequently, hydrothermal temperatures and steep chemical gradients emerge as environmental selection factors that become more important downcore, driving changes in fungal populations in deeper sediment samples. Because our DNA-based approach detects both viable cells and non-viable extracellular DNA [84], this inference requires further inquiry using RNA- and culture-based investigations. The taxonomic similarity of surface samples and divergence of deeper samples, respectively, is substantiated by fungal ordination analysis, where surficial and mid-core samples are tightly clustered, whereas most deeper sediment samples show a widely scattered pattern (Fig 5). In contrast to bacteria and archaea, the fungal sequences do not cluster by sampling area (S14 Fig in S1 File). Diversity indices of the fungal populations reflect the impact of temperature and sediment depth, independent of sampling area but linked to sedimentation; chytrid-dominated populations in cooler, surficial sediments contrast with other fungal populations in deeper, hotter sediments (S15 Fig in S1 File).

Microbial co-occurrence networks and associated metrics that characterized the hydrothermal sediments of Guaymas Basin suggest stronger co-occurrences within domains, and weaker inter-domain (archaea-bacteria, archaea-fungi and bacteria-fungi) associations. In other words, representatives of a microbial domain form limited interactions with ASVs from other domains, but form a cohort with numerous other ASVs within the same domain, i.e. fungal ASVs were correlated only with other fungal ASVs. These interactions could suggest intra-domain cooperative metabolisms, and potentially inter-domain competition for resources. However, this would require further investigation, considering that in environmentally heterogeneous ecosystems the dispersal and co-occurrence patterns of microbial communities can be jointly driven by deterministic factors, such as specific environmental controls, as well as by stochastic, ecologically neutral processes [85–87].

The absence of correlations between fungal and bacterial/archeal ASVs implies that the fungal community in surficial sediments in Guaymas Basin might not control the structure of functional microbial networks and interactomes, in contrast to what has been described for other habitats [88–90] where co-occurrences between bacteria and fungi resulted from both synergism and antagonism [91], with fungi serving as keystone species that stabilize network properties in complex ecosystems [89, 90]. However, stochastic ‘mixing’ can mask causal relationships between bacteria and fungi within networks [91]. We hypothesize that the absence of bacterial-fungal co-occurrences in Guaymas Basin sediments may result from such stochastic ‘mixing’ in this dynamic ecosystem where hydrothermal fluids passing through the thick organic-rich sediments have variable residence times and follow ephemeral pathways [92–94].

While both positive and negative interactions were observed between bacterial and archaeal ASVs close to the sediment surface, these interactions lessened with depth (Fig 7). In contrast, co-occurrences between the most abundant fungal ASVs continued at depth. Fungal co-occurrences exist in complex ecosystems and fungal-fungal interactions are thought to be triggered by largely unknown chemoattractive mechanisms, or by formation of hyphal and mycelial networks [95]. Hyphal and mycelial networks have been described to have ecological “memory” that influences niche partitioning and fungal growth towards new resources [96] while also shaping the plastic behavior that allows fungi to cope with ephemeral resources and competition [97]. Although speculative for Guaymas sediments, the existence of chemoattractive mechanisms and mycelial networks could explain the observed co-occurrences between fungal ASVs at deeper sediment depths.

The absence of inter-domain network interactions involving fungi, and reduced prokaryotic inter-domain network complexity at depth may be linked to harsher environmental conditions downcore that increasingly interfere with microbial interactions. For example, decreasing cell densities downcore due to increasing temperatures may increase the distances between potentially interactive cells and limit the magnitude of interactions. Overall, cross-domain microbial interactions and associations appear to be overwritten by environmental selection factors at Guaymas Basin, whereas other marine [98] or terrestrial [99] habitats show significant cross-domain associations.

Potential selection factors that overwrite microbial interactions and associations in Guaymas Basin include hydrothermal carbon and energy sources. Methane is a dominant carbon species in Guaymas Basin surficial sediments [14] and its availability influences microbial community composition. Stable carbon-isotopic signatures of methane carbon in Cathedral Hill and Marker 14 samples show the isotopic imprint of methane oxidation, as δ^13^C-CH_4_ values shift from the hydrothermal baseline of the southern Guaymas Basin vent field (near -42 to 43‰, [14]) towards heavier (less negative) values (S5 Table in S1 File). Consistent with this isotopic evidence for microbial methane oxidation, hydrothermal sediments in the Cathedral Hill and Marker 14 area have high proportions of ANME archaea, especially ANME-1 and ANME-1Guaymas lineages (S10, S13 Figs in S1 File). Interestingly, the light δ^13^C-CH_4_ values (near -50 ‰) in the Aceto Balsamico sediments (S5 Table in S1 File) suggest that a contribution of microbial methane in these temperate sites mutes the isotopic consequences of anaerobic methane oxidation. Microbial methane in Guaymas Basin and the nearby Sonora Margin has δ^13^C-CH_4_ values of –70 to –80 ‰ [14, 100], and in this regard resembles the δ^13^C-CH_4_ values of trace methane in the background sediment (S5 Table in S1 File). In addition to methane, the availability of hydrothermally produced hydrocarbons influences the microbial community. Several sulfate-reducing lineages within the Deltaproteobacteria (i.e. *Desulfatiglans* lineage, *Desulfobacteraceae* and *Syntrophaceae*) are well-represented, particularly in Marker 14 and Acetobalsamico (S11 and S12 Figs in S1 File), and these lineages specialize in direct or syntrophic oxidation of aromatic and aliphatic hydrocarbons (reviewed in [22]).

The impact of specific carbon substrates on the fungal community is unresolved. Potential carbon sources for fungi in marine sediments that are accessed via exoenzyme secretion include refractory polysaccharides [101] and refractory carbohydrates (e.g., beta-glucans) [102]. Fungi take part in degradation of hydrocarbons in soils and sediments, including aromatic compounds [103–105]. Physiologically versatile fungi persist in the deep subsurface biosphere, and have been cultivated from coal-bearing deep subsurface sediments at 2 km depth [106, 107]. Over 100 fungal genera are known to play important roles in biodegradation [108]. The site-specific hydrothermal fungal communities (with numerous uncharacterized members) might access available substrates in Guaymas Basin, but intergenic spacer sequences do not specifically identify fungi known to utilize hydrocarbons as a primary carbon source. Yet, novel fungi might tap into the rich reservoir of complex petroleum and light-hydrocarbon compounds produced by the thermal alteration of organic matter [109, 110]. This possibility requires future investigation using fungal enrichment cultures and isolates from Guaymas Basin grown on selected combinations of hydrocarbon substrates to elucidate the metabolic potential of taxonomically unresolved fungal populations.

This study directs our attention to zoosporic fungi as a major component of this fungal dark matter, *i*.*e*. the uncultured and poorly know taxa affiliated to basal fungal lineages such as Rozellidea and Chytridiomycota [55, 111]. In the marine environment, some zoosporic fungi (*Chytridiomycota*, *Blastocladiomycota*) have been described by microscopy and DNA sequencing, but they are infrequently reported, and successful culturing efforts are restricted to a few marine isolates [112]. Sequencing surveys of zoosporic fungi in aquatic habitats have uncovered unexplored biodiversity, and have placed *Chytridiomycota* into “dark matter fungi” [111]. Chytrid orders detected in Guaymas Basin–*Rhizophydiales*, *Rhizophlyctidales* and *Spizellomycetales*–are linked to inputs from particle flux from the upper water column, and sedimentary particles delivered via tectonic and current-driven sediment flows, and riverine inputs. The *Rhizophydiales* are primary organic decomposers and have been reported in marine sediments (Kongsfjorden, Svalbard) of the High Arctic [113], and in ice cores and seafloor sediment samples from the Alaskan coastal Arctic [69]. Novel chytrids of the order *Rhizophydiales* are described as parasites of dinoflagellates and diatoms [68, 70]. The *Rhizophlyctidales* include mainly soil-inhabiting, cellulose-degrading chytrids, isolated from terrestrial ecosystems [114]. The *Spizellomycetales* contain saprophytic chytrids that sometimes grow inside pollen grains buffered against environmental stresses [115]. Abundant *Rhizophlyctidales* and *Spizellomycetales* 18S rRNA gene sequences from high-altitude soils group with environmental sequences from diverse locations, including deep anoxic marine sediments of Cariaco Basin and high-altitude soils [116, 117]. OTUs belonging to *Spizellomycetales* were identified from deep-sea sediments in the Magellan seamounts [118], although members of this group are thought to be mainly of terrestrial origin. Their survival and/or activity in the deep sea remain open questions.

In addition to the chytrids, other zoosporic fungi are present in Guaymas Basin. The *Neocallimastigomycota*, an early-diverging lineage of zoosporic anaerobic fungi that lack mitochondria and contain hydrogenosomes, are represented by eight ASVs; they are primarily associated with the guts of herbivorous mammals and contain an expanded repertoire of cellulolytic enzymes [55, 119, 120]. The *Neocallimastigomycota* Guaymas ASVs affiliate loosely with *Piromyces* sp. and uncultured *Neocallimastigales*, with the caveat that BLAST alignments show a low percentage identity (~80%) and coverage (~45%). Different *Piromyces* species are able to generate hydrogen, acetate and formate as end‐products of a prokaryotic‐type mixed‐acid fermentation in the rumen of herbivores, e.g. goats [121]. Cultivated *Neocallimastigomycota* isolates so far require growth temperatures of 39°C [121, 122]. The surficial Guaymas sediments that harbor *Neocallimastigomycota* ASVs had a measured temperature range between 3–15°C, however, fermentation of complex carbohydrates to short-chain fatty acids and hydrogen are plausible processes under the biogeochemical regime of Guaymas sediments, and temperatures are known to fluctuate in hydrothermal sediments. Interestingly, fungal hydrogenosome-based metabolism producing H_2_ may feed methanogens and other hydrogen-consuming anaerobic archaea in deep subsurface habitats [123, 124], and thus fungi might participate indirectly in methane and hydrogen cycling.

The shift from chytrids towards other fungal communities below the surficial sediment layers could in part be linked to elevated temperatures that range between 30°-50°C in some of the deeper sediments examined here. Both Ascomycota and Basidiomycota include thermotolerant taxa (e.g. *Saccharomycetes* and *Malassseziomycetes*) that are able to grow between 30°C to 45°C [125]. Most of the currently described *Malassezia* yeasts are characterized by their ability to grow at elevated temperatures, such as 45°C [125]. Culture-based and metabarcoding analyses of different substrate types at shallow hydrothermal vents [126], deep-sea hydrothermal sediments [59] and deep-sea hydrothermal vents [83, 127] also reveal *Agaricomycetes*, *Malasseziomycetes*, and *Saccharomycetes* as dominant taxa within fungal communities in those hydrothermal vent ecosystems. Future laboratory studies can reveal the capabilities of diverse chytrids and other zoosporic fungi to grow on diverse carbon sources that are available in Guaymas Basin sediments. These include marine phytoplankton biomass or terrestrial organic matter, as well as diverse hydrocarbons. High-temperature cultivations are likely to yield thermotolerant or even thermophilic fungi among the *Ascomycota* and *Basidiomycota*. The shift towards elevated temperatures deeper in these cores may coincide with changing modes of fungal nutrition and changing substrate preferences, from hydrolysis and fermentation of plankton-derived polymers towards hydrocarbon utilization.

## Conclusions

**Fig 9:**
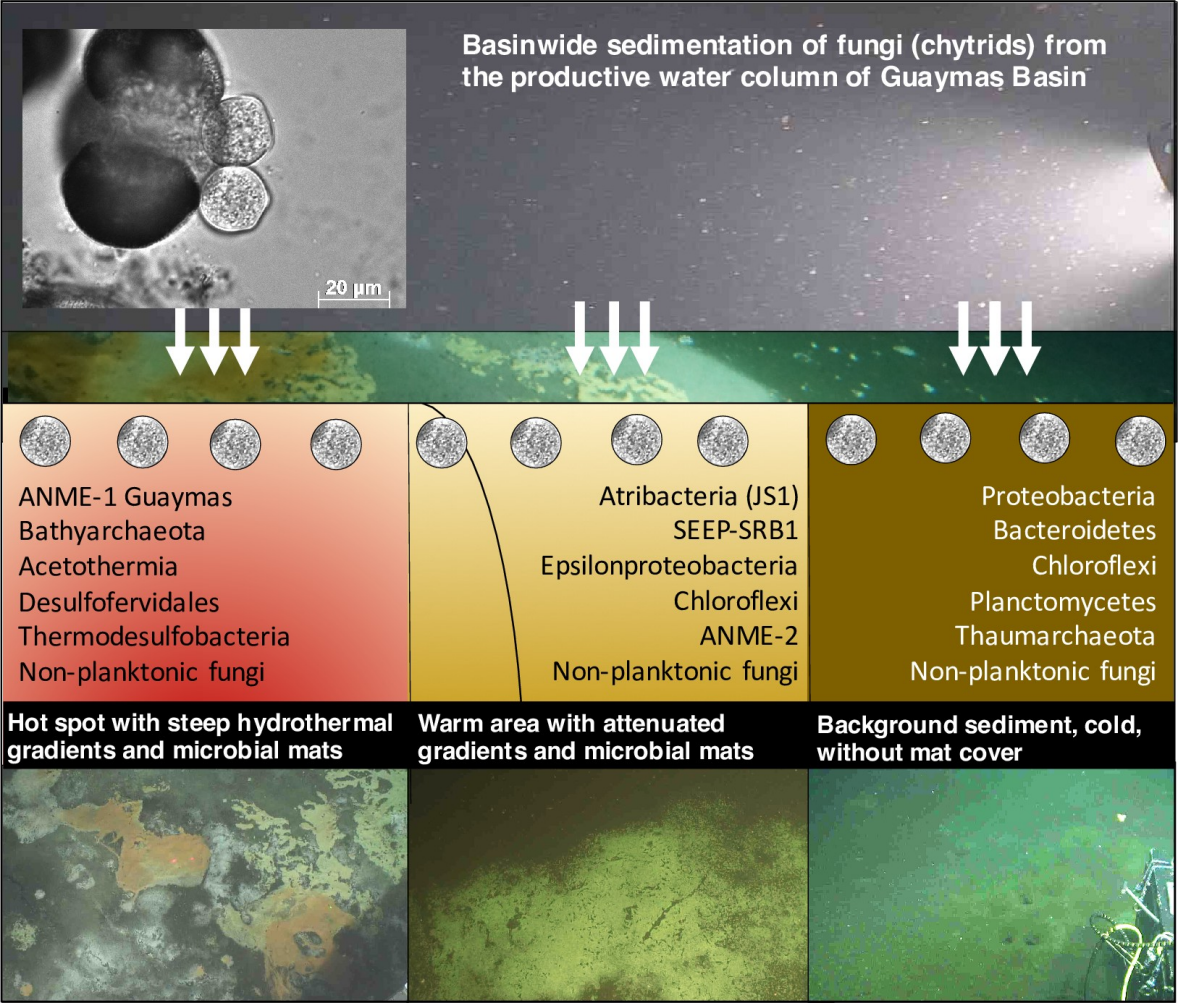
Conceptual summary. Localized hydrothermal gradients and characteristic microbial populations in Guaymas Basin sediments are superimposed on location-independent biogenic sedimentation from the productive water column, the likely source of ubiquitous chytrid biosignatures in surficial sediments. The insert image at the top left shows chytrids isolated from the estuarine water column of Salt Pond, Falmouth, MA growing on a pollen grain (image courtesy of Edgcomb lab).

The establishment and structuring of cohabitating prokaryotic and fungal communities in Guaymas Basin surficial sediments respond to fundamentally different environmental cues. For bacteria and archaea, varying thermal and geochemical states dictate site-specific community composition with depth-dependent decreases in community richness, and increases in the relative abundance of archaea. Concentrations of methane and sulfate, temperature, and the presence of aromatic and aliphatic hydrocarbons impact sedimentary microbial communities. In contrast, fungal communities in surficial sediments from different sites are mutually similar, implying a “regional blanketing” of fungi dominated by chytrids (Fig 9). While chytrids persist at all sediment depths sampled at the relatively cool background and temperate sites, the steep thermal gradients at Cathedral Hill result in downcore relative enrichment of both unidentified and known thermotolerant fungal lineages over chytrids.

Several avenues are opening for future fungal research in Guaymas Basin. To determine the likely sources of chytrids and other zoosporic fungi, targeted cultivations should be combined with tests of their ability to grow on marine phytoplankton biomass or terrestrial organic matter. High-temperature cultivations are likely to yield thermotolerant or even thermophilic fungi among the *Ascomycetes* and *Basidiomycetes*, but potentially in other lineages as well. Elevated temperatures may also drive changes in modes of nutrition and substrate spectra, from hydrolysis and fermentation of plankton-derived polymers towards hydrocarbon utilization. Importantly, this study highlights different ecological responses of the prokaryotic and fungal sedimentary community fractions. Understanding how each community responds to site-specific environmental challenges and opportunities will shed light on the nature, extent, and impact of microbial carbon cycling in the diverse hydrothermal environments of Guaymas Basin.

## Supporting information

S1 File (PDF)
