# Supplementary material for: Environmental factors shaping bacterial, archaeal and fungal community structure in hydrothermal sediments of Guaymas Basin, Gulf of California
Source: PLoS One. 2021 Sep 8;16(9):e0256321. doi: 10.1371/journal.pone.0256321 (PMC8425543; doi:10.1371/journal.pone.0256321)
Supplement: S1 File — (PDF) [file pone.0256321.s001.pdf]

Supplementary Materials

Environmental factors shaping bacterial, archaeal and fungal community structure in hydrothermal sediments of Guaymas Basin, Gulf of California

Gustavo A. Ramírez, Paraskevi Mara, Taylor Sehein, Gunter Wegener,
Christopher R. Chambers, Samantha B. Joye, Richard Peterson,
Aurélie Philippe, Gaëtan Burgaud, Virginia P. Edgcomb, Andreas P. Teske

Supplementary Methods

Phylogenetic inferences. Phylogenetic distance trees for representative Methanomicrobia and Deltaproteobacteria ASV sequences were inferred with the program package PAUP4.0 (Swofford 2000), using Minimum Evolution as the optimality criterion, and checking branching patterns with 1000 neighbor-joining bootstrap replicates. The ASV sequences that appear in the phylogenies are included in the supplementary material for easy retrieval (S7 Table).

Bioinformatics analyses. To visualize the relative abundances of various fungal groups across samples in a bubble plot, non-normalized sequence read counts were clustered by class when the taxonomic annotation specified and by phyla when higher classification was not available. Principal Coordinate Analysis (PCoA) of Bray-Curtis distances implemented in R (version 3.6.1), using the vegan (Oksanen et al. 2019) and phyloseq (McMurdie and Holmes 2013) packages, was used to visualize fungal community clustering as a function of collection site and sediment depth.

Supplementary Results

Community Richness. Generally, microbial community richness, measured as observed Amplicon sequence variants (ASVs), is higher in background sediment (by factor 1.5 to 2) than in hydrothermal sediment (Figure S2). The highest richness estimates at any sampled site were reported in the background 0-5 cm depth interval (2662 observed ASVs). Observed ASVs for the different background sediment depth intervals did not differ significantly (t-test, alpha = 0.05, used for all significance tests). At hydrothermal sites, richness generally follows a decreasing trend downcore. In Aceto Balsamico sediment, an average of 1495, 1,093, and 989 ASVs were observed for 0-10, 10-20, and 20-30 cm depth, respectively (Figure S2, Table S5). In Cathedral Hill communities, the observed ASVs average decreased from 1,756 in the surface segment to 716 downcore (Figure S2, Table S5). The Marker 14 community richness also generally decreases with depth. Despite the clear trends of decreasing richness with sediment depth at hydrothermal sites, perhaps due to limited statistical power from the low number of observations ($n < 5$), the means across depth intervals per site are not significantly (t-test, alpha = 0.05) different.

Methanomicrobia and Deltaproteobacteria. The observed occurrence patterns of bacterial and archaeal ASVs prompted a closer phylogenetic analysis of ASVs from microbial lineages where cultured isolates, enrichments and ecophysiological information are available. We selected the deltaproteobacterial sulfate reducers and the Methanomicrobia, due to their relevance for hydrocarbon and methane cycling in Guaymas Basin sediments, and their frequent occurrence among ASV sequences. The sulfate-reducing Desulfofervidales are also included, since full genome analyses tend to affiliate this deeply-branching 16S rRNA lineage (McKay et al. 2016) with the Deltaproteobacteria (Waite et al. 2020).

Within the Methanomicrobia, ASVs are affiliated with methane-oxidizing archaea of the ANME-1 and ANME-2 lineages, and the cultured family *Methanomicrobiaceae*; often they are closely related to, or identical to phylotypes that were previously recovered from Guaymas Basin (S9 Fig). ANME-1 archaea are represented by ASVs 10, 13, 55, 135, 174, 198, 223, and 237. The ANME-1Guaymas lineage, a sister lineage of the globally distributed ANME-1 archaea, is preferentially found in hydrothermal sediments (Biddle et al. 2012) and is represented by several ASVs (Nos. 7, 58, 84, 126, 155, 239). The ANME-2c lineage within the Methanosarcinales is represented by ASVs 3, 52, 124, 183, 243, 256 and 300, ANME-2ab is represented by ASVs 132 and 305, and ANME-2d by ASVs 170 and 186 (S9 Fig).

The changing frequencies of the methanomicrobial ASVs across the sample set are visualized in a balloon plot (S10 Fig). ANME-1 phylotypes are found in surface and subsurface Cathedral Hill sediments (ASVs 10, 13, 55) and surficial Marker 14 samples (ASVs 13, 135), consistent with broad thermal adaptability among Guaymas ANME-1 populations (Holler et al. 2011). Members of the distinctly branching ANME-1Guaymas lineage are found preferentially in Cathedral Hill subsurface sediments, consistent with its preferential detection in hydrothermally-active subsurface sediments underneath *Beggiatoaceae* mats (Biddle et al. 2012, McKay et al. 2016). Members of the ANME-2 lineages appear to avoid high temperatures (McKay et al. 2016), as shown by their ASV occurrence pattern. ANME-2ab is found only in some temperate Aceto Balsamico sediments (ASV 132, 305). ANME-2c, including the highly abundant ASV 3, constitutes the dominant methanomicrobial lineage in temperate Aceto Balsamico and surficial Marker 14 samples. Other ASVs of this lineage are also found in Cathedral Hill (ASVs 52, 243). Archaea of the ANME-2d lineage, represented by the ASVs 170 and 186, appear in traces

throughout all samples. In the background sediments, signatures of Methanomicrobia are conspicuously absent or only detected at extremely low abundances (S10 Fig).

Within the Deltaproteobacteria, ASVs are affiliated with lineages of cultured sulfate-reducing bacteria and with uncultured family-level lineages (S11 Fig). The *Desulfobacteraceae*, a physiologically diverse family of sulfate reducers that completely oxidize low-molecular-weight substrates such as acetate, LMW-organic acids, alkanes and aromatics (Küver 2014; Teske 2019), are represented by ASVs 9, 42, 51, 108, 123, 127, 131, 149, 154, 189, 215, 216, 228 and 259 (S11 Fig). Of these, the ASVs 9, 51 154 and 189 are members of the SEEP-SRB1 lineage, sulfate-reducing syntrophs of methane-oxidizing archaea at cool and temperate conditions (Knittel et al. 2003, Schreiber et al. 2010). The family-level *Desulfatiglans* lineage, harboring ASVs 125, 162 and 301, consists of isolates that oxidize substituted mono- and polycyclic aromatics (summarized in Teske et al. 2019), whereas genome analyses suggest an additional role in reductive dehalogenation in marine subsurface sediments (Jochum et al. 2018). The Desulfuromonadales, represented by ASVs 59 and 163, encompass sulfur-reducing, iron-reducing, and fermentative bacteria that commonly thrive on acetate and other LMW organic acids (Küver 2005). The Syntrophobacterales, represented by ASV 281, include sulfate-reducing (sometimes thermophilic) but also fermentative bacteria that grow in syntrophic association with H_2/formate-utilizing bacterial partners (Küver 2014). Uncultured lineages within the incompletely oxidizing family *Desulfobulbaceae*, the seep-associated lineages SEEP-SRB3 and SEEP-SRB4, are represented by ASVs 39, and 16 and 76, respectively. The mesophilic, sulfate-reducing, potentially alkane-oxidizing SEEP-SRB2 lineage (Krukenberg et al. 2018) is represented by ASVs 14, 34, 69, and 254. A lineage of deep subsurface clones, annotated as members of the family *Desulfoarculaceae* (Davidova et al. 2016) but only distantly related to these cultures (Teske et al. 2019), is represented by ASVs 219 and 251. So far, only two lineages, the *Thermodesulfobacteraceae* and the *Desulfofervidales*, are unequivocally linked to sulfate reduction at high temperatures. The *Thermodesulfobacteraceae*, represented by ASVs 12 and 217, have temperature optima of 65-75°C and can grow lithoautotrophically with CO_2/H_2 or heterotrophically with LMW organic acids (Jeanthon et al. 2002). The Desulfofervidales, represented by ASVs 22, 46, 147, 172, 289, 293 and 302 (S11 Fig), have a growth optimum of 50-60°C and form syntrophic consortia with methane- and short-chain alkane-oxidizing thermophilic archaea (Laso-Perez et al. 2016, Hahn et al. 2020), but they can also thrive as free-living hydrogenotrophs (Krukenberg et al. 2016).

The balloon plot of changing ASV frequencies across the sample set (S12 Fig) shows a pattern of widespread occurrence for the members of cultured sulfate-reducing families (*Desulfosarcinaceae*, *Desulfobacteraceae*, *Syntrophaceae*, *Desulfatiglans* lineage) that co-exist with a pattern of conspicuous site-specific occurrence peaks for some groups, for example SEEP-SRB1 in Aceto Balsamico, SEEP-SRB2 in Marker 14, and SEEP-SRB4 in surficial samples of Aceto Balsamico. The thermophilic *Thermodesulfobacteraceae* and *Desulfofervidales* occur sporadically in Cathedral Hill samples, and surprisingly also in some deeper Aceto Balsamico samples (S12 Fig). The background sediment sharply contrasts with all other sites by its specific ASV signature, consisting of the taxonomically unassigned ASV 208, ASV 123 within the *Desulfobacteraceae*, and ASVs 219 and 251, the subsurface lineage that is distantly related to the *Desulfoarculaceae* (S12 Fig).

Bar plots of *Desulfobacteraceae* and *Methanomicrobiales* show their site-specific compositional changes (S13 Fig). In Aceto Balsamico sediments, the SEEP-SRB1 group dominates the Desulfobacteraceae, whereas the relative proportion of SEEP-SRB1 is reduced in favor of phylotypes affiliated with cultured genera (aromatics-oxidizing *Desulfobacula spp.*, hydrogenotrophic *Desulfoconvexum spp*, and acetate-oxidizing *Desulfobacterium spp.*) in the Marker 14 samples, and nutritionally versatile *Desulfonema* spp. in the Cathedral Hill samples (S13 Fig). The Methanomicrobiales and Methanosarcinales that dominate in Aceto Balsamico are supplemented and outcompeted by ANME-1 in Marker 14 and Cathedral Hill samples, respectively (Figure S8), consistent with trends in the ASV balloon plots.

Supplementary references

Biddle JF, Cardman Z, Mendlovitz H, Albert DB, Lloyd KG, Boetius A, Teske A. Anaerobic oxidation of methane at different temperature regimes in Guaymas Basin hydrothermal sediments. ISME J. 2012; 6, 1018-1031.

Davidova IA, Wawrik B, Callaghan AV, Duncan K, Marks CR, Suflita JM. *Dethiosulfatarculus sandiegensis* gen. nov., sp. nov., isolated from a methanogenic paraffin-degrading enrichment culture and emended description of the family *Desulfarculaceae*. Int J Syst Evol Microbiol. 2016; 66: 1242-1248.

Hahn C, Laso-Pérez R, Volcano F, Vaziourakis KM, Stokke R, Steen IH, Teske A, Boetius A, Liebeke M, Amann R, Knittel K, Wegener G. 2020. "*Candidatus* Ethanoperedens", a thermophilic genus of Archaea mediating the anaerobic oxidation of ethane. mBio 2020; 11: e00600-20, doi:10.1128/mBio.00600-20

Holler T, Widdel F, Knittel K, Amann R, Kellermann MY, Hinrichs KU, Teske A, Boetius A, Wegener G. Thermophilic anaerobic oxidation of methane by marine microbial consortia. ISME J. 2011; 5: 1946-1956.

Jeanthon C, L'Haridon S, Cueff V, Banta A, Reysenbach AL, Prieur D. *Thermodesulfobacterium hydrogenophilum* sp. nov., a thermophilic, chemolithoautotrophic, sulfate-reducing bacterium isolated from a deep-sea hydrothermal vent at Guaymas Basin, and emendation of the genus *Thermodesulfobacterium*. Int J Syst Evol Microbiol. 2002; 52: 765-772.

Jochum LM, Schreiber L, Marshall IPG, Jørgensen BB, Schramm A, Kjeldsen KU. Single-cell genomics reveals a diverse metabolic potential of uncultivated *Desulfatiglans*-related Deltaproteobacteria widely distributed in marine sediment. Front Microbiol. 2018; 9: 2038.

Knittel K, Boetius A, Lemke A, Eilers H, Lochte K, Pfannkuche O, Linke P, Amann R. Activity, distribution, and diversity of sulfate reducers and other bacteria in sediments above gas hydrate (Cascadia Margin, OR). Geomicrobiol J. 2003; 20: 269–294

Krukenberg V, Harding K, Richter M, Glöckner FO, Gruber-Vodicka HR, Adam B, Berg J, Knittel K, Tegetmeyer HE, Boetius A, Wegener G. *Candidatus* Desulfofervidus auxilii, a hydrogenotrophic sulfate-reducing bacterium involved in the thermophilic anaerobic oxidation of methane. Environ Microbiol. 2016; 18: 3073–3091.

Krukenberg, V, Riedel D, Gruber-Vodicka HR, Buttigieg PL, Tegetmeyer HE, Boetius A, Wegener G. Gene expression and ultrastructure of meso- and thermophilic methanotrophic consortia. Environ. Microbiol. 2018; 20, 1651-1666.

Küver J. The family *Desulfobacteraceae*. Pp 45-73. In: Rosenberg E, DeLong EF, Lory S, Stackebrandt E, Thompson F (eds) The Prokaryotes – Deltaproteobacteria and Epsilonproteobacteria. Springer, Berlin/Heidelberg, 2014; doi:10.1007/978-3-642-39044-9_266

Küver J, Rainey FA, Widdel F. Order V. *Desulfuromonales* ord. nov. *In:* Brenner DJ, Krieg NR, Staley JT, Garrity GM (eds), Bergey's Manual of Systematic Bacteriology, 2nd edn, Vol. 2 (The Proteobacteria), Part C (The Alpha-, Beta-, Delta and Epsilonproteobacteria), Springer, New York. 2005; 1005-1006.

Laso-Pérez R, Wegener G, Knittel K, Widdel F, Harding KJ, Krukenberg V, Meier DV, Richter M, Tegetmeyer HE, Riedel D, Richnow HH, Adrian L, Reemtsma T, Lechtenfeld O, Musat F. Thermophilic archaea activate butane via alkyl-coenzyme M formation. Nature 2016; 539: 396–401.

McKay L, Klokman V, Mendlovitz H, LaRowe D, Zabel M, Hoer D, Albert D, de Beer D, Amend J, Teske A. Thermal and geochemical influences on microbial biogeography in the hydrothermal sediments of Guaymas Basin. Environ Microbiol Rep. 2016; 8:150-161.

McMurdie PJ, Holmes S. Phyloseq: an R package for reproducible interactive analysis and graphics of microbiome census data. PLoS One 2013; 8: e61217.

Oksanen J, Blanchet FG, Friendly M, Kindt R, Legendre P, McGlinn D, Minchin PR, O'Hara RB, Simpson GL, Solymos P, Stevens MHH, Szoecs E, Wagner H. Vegan: Community Ecology Package. R package version 2.5-6. 2019; https://CRAN.R-project.org/package=vegan

Schreiber L, Holler T, Knittel K, Meyerdierks A, Amann R. Identification of the dominant sulfate-reducing bacterial partner of anaerobic methanotrophs of the ANME-2 clade. Environ Microbiol. 2010; 12: 2327–2340.

Swofford DL. PAUP*. Phylogenetic Analysis Using Parsimony (and Other Methods), 4th ed.

[CD-ROM]; Sinauer Associates: Sunderland, MA, USA. 2000.

Teske A. Hydrocarbon-degrading anaerobic microbial communities in natural oil seeps**.** In: McGenity TJ (ed), Microbial Communities Utilizing Hydrocarbons and Lipids: Members, Metagenomics and Ecophysiology, Handbook of Hydrocarbon and Lipid Microbiology. Springer. 2019, doi: 10.1007/978-3-319-60063-5_3-2

Waite DW, Chuvochina M, Pelikan C, Parks DH, Yilmaz P, Wagner M, Loy A, Naganuma T, Nakai R, Whitman WB, Hahn MW, Kuever J, Hugenholtz P. Proposal to reclassify the proteobacterial classes *Deltaproteobacteria* and *Oligoflexia*, and the phylum *Thermodesulfobacteria* into four phyla reflecting major functional capabilities. Int J Syst Evol Microbiol. 2020; 70: 5922-6016

Supplementary Figures

Supplementary Figure S1. Bathymetric map of sampling locations in the southern axial valley of Guaymas Basin, recorded by *AUV* Sentry.

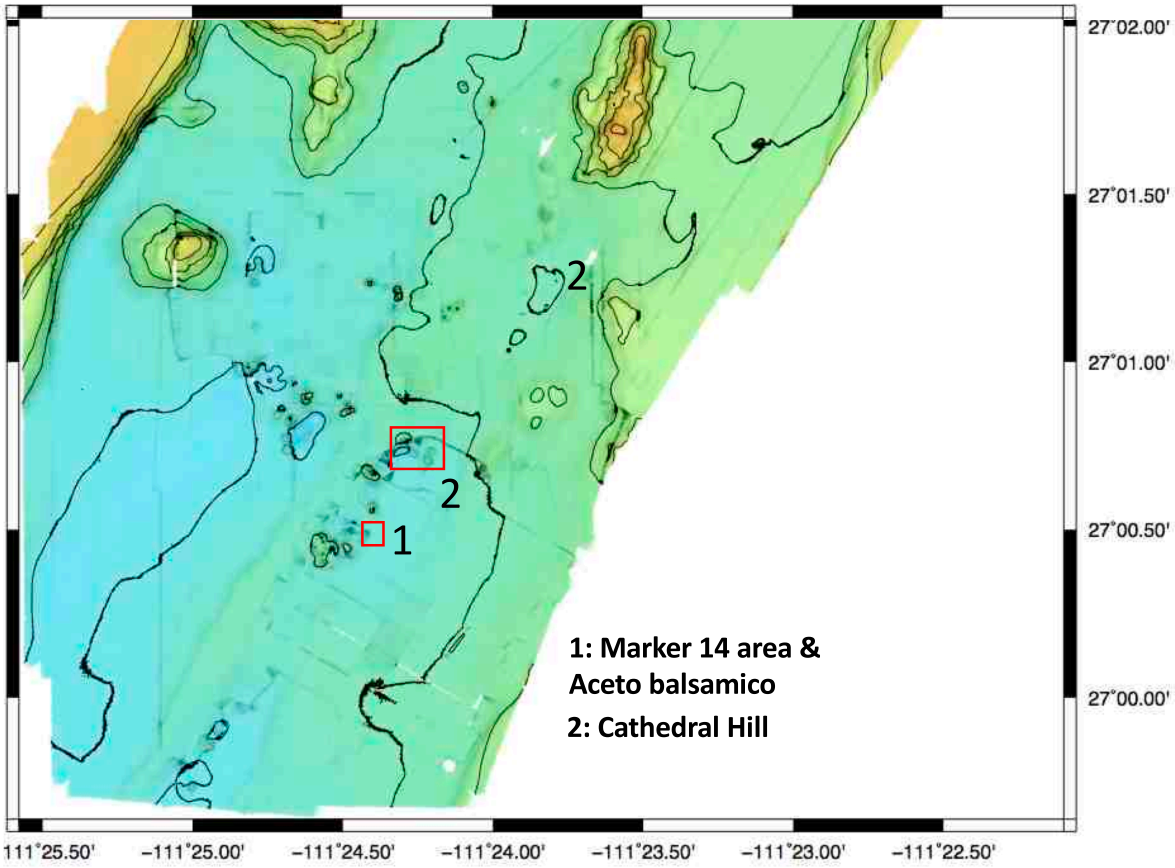

Supplementary Figure S2. Observed ASVs for Bacterial and Archaeal communities in Aceto Balsamico, Background, Cathedral Hill and Marker 14 sediments, color-coded by sample depth

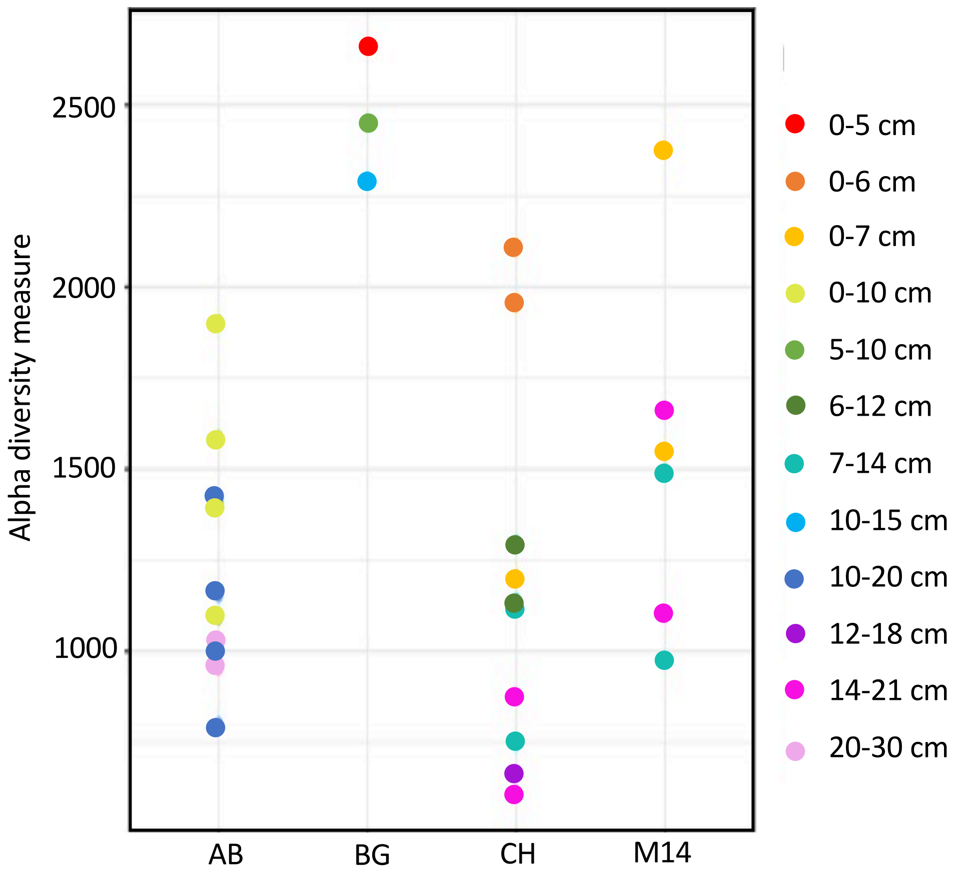

Supplementary Figure S3. Relative proportions of bacterial and archaeal 16S rRNA gene sequence amplicons obtained from Guaymas Basin sediment samples (sum = 100%)

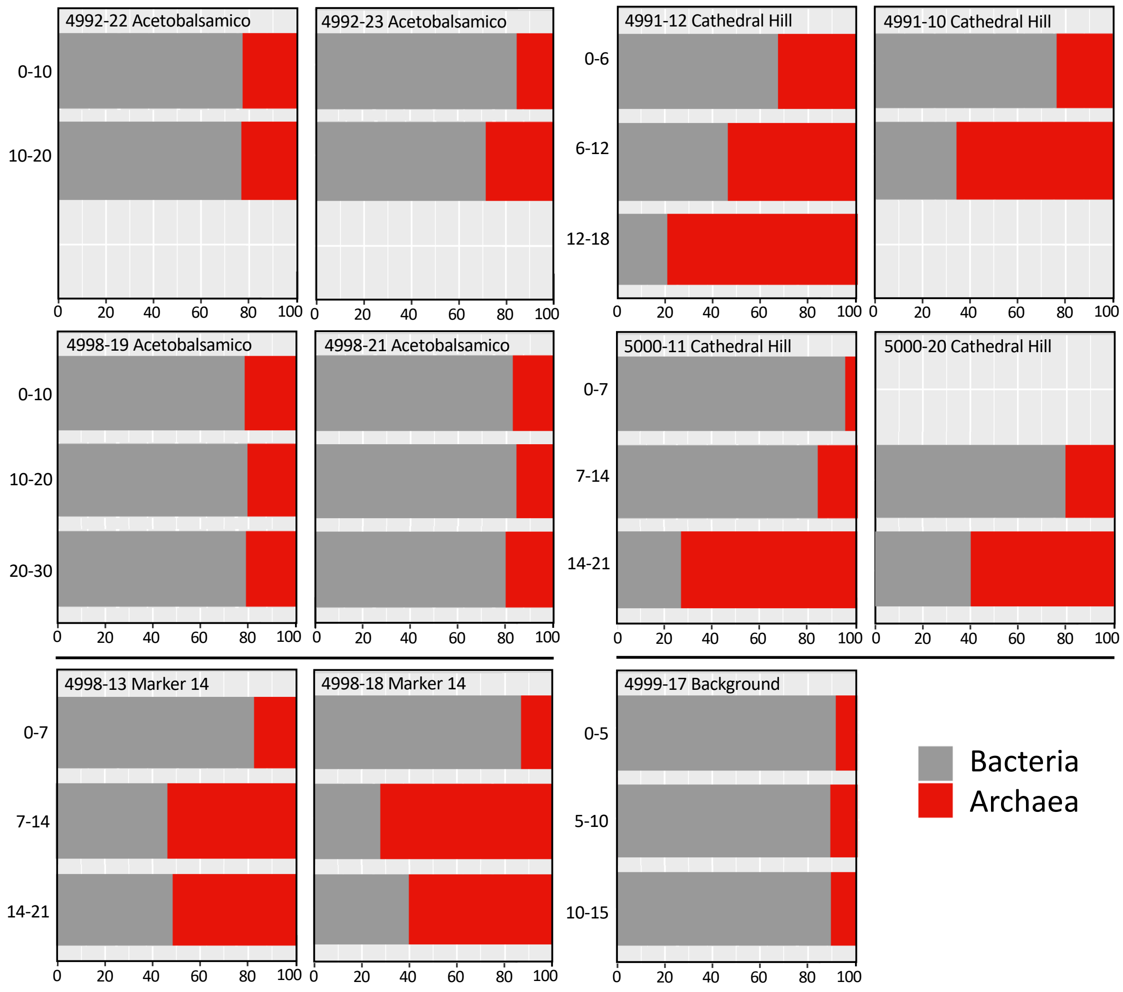

Supplementary Figure S4. Phylum-level composition of bacterial and archaeal communities in Guaymas Basin sediments, according to SILVA 132 taxonomy annotations.

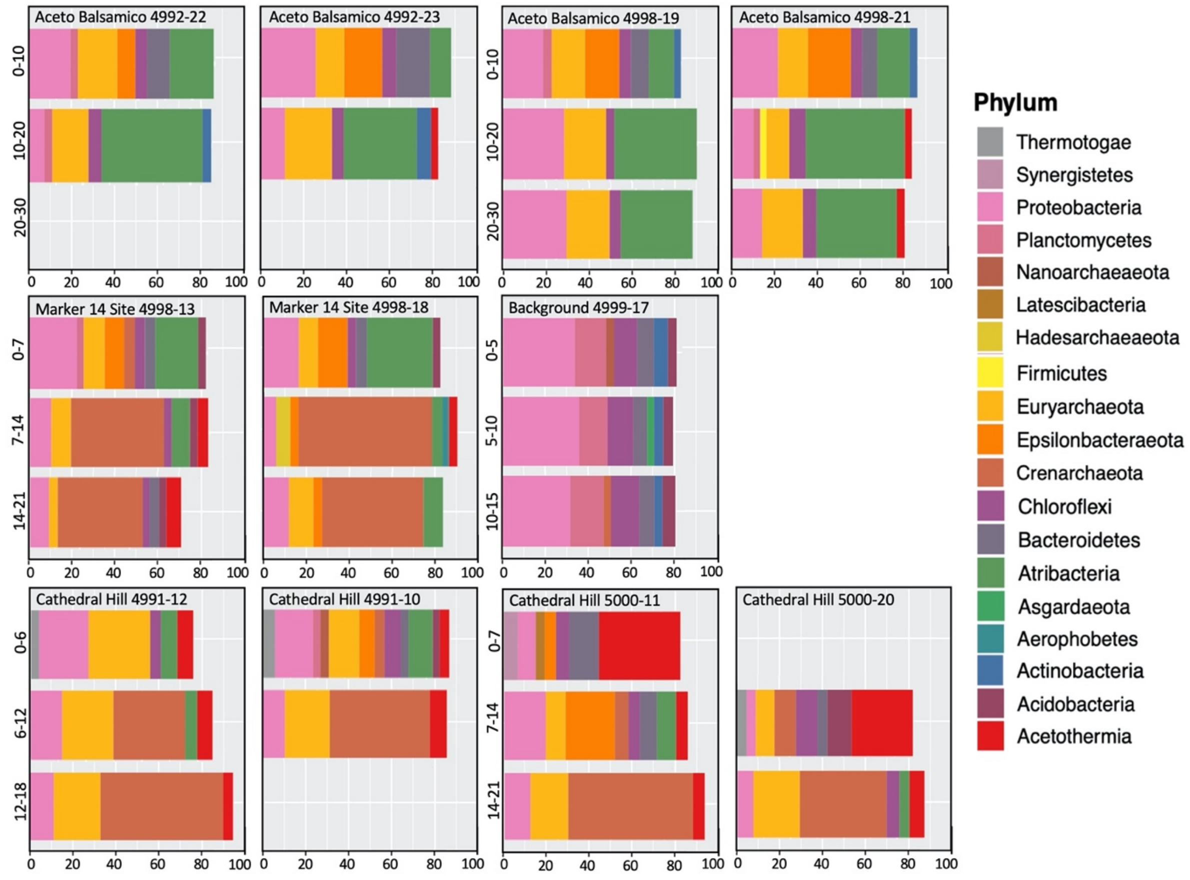

Supplementary Figure S5. Class-level composition of bacterial and archaeal communities in Guaymas Basin sediments, according to SILVA 132 taxonomy annotations.

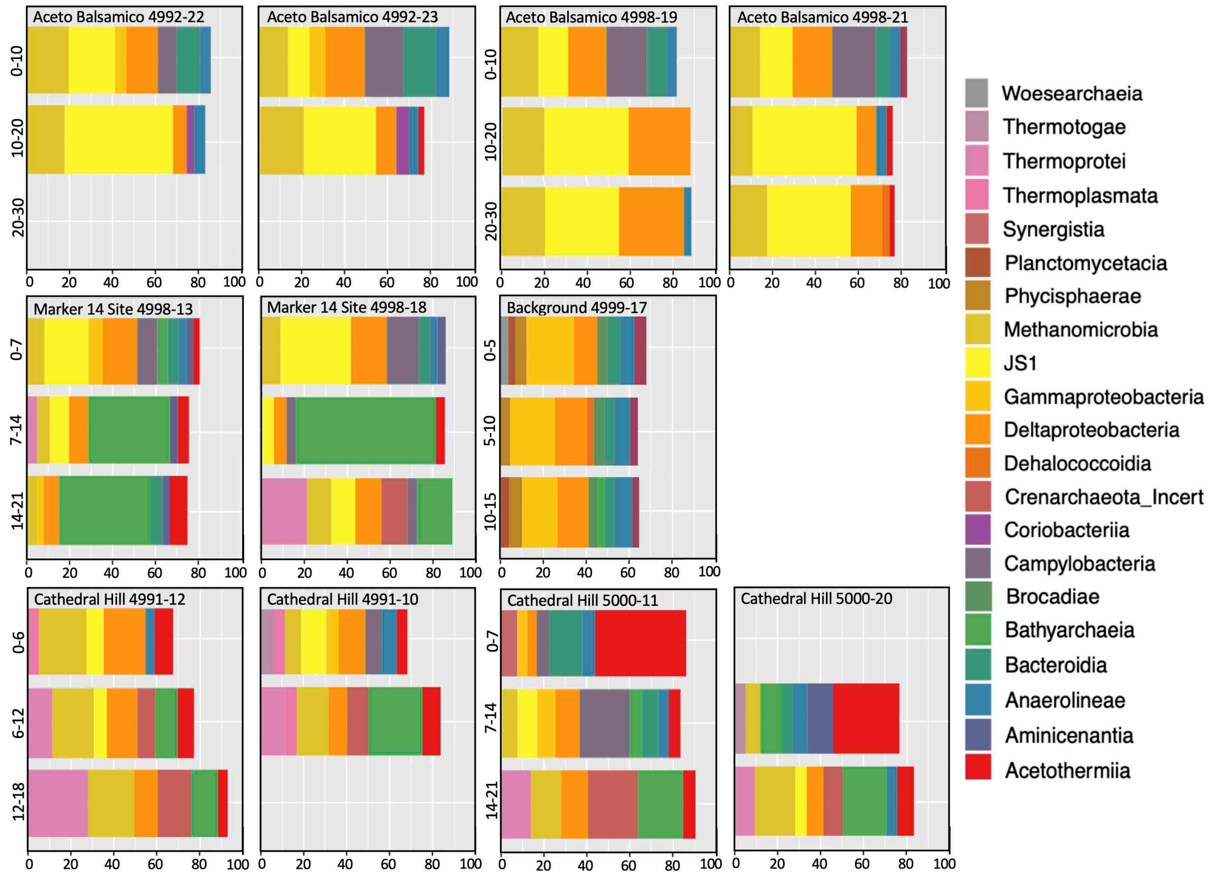

Supplementary Figure S6. PCoA plot of Bacterial and Archaeal sequences in Guaymas Basin sediments as shown in Figure 3, further annotated by sediment core numbers and depth intervals in centimeters.

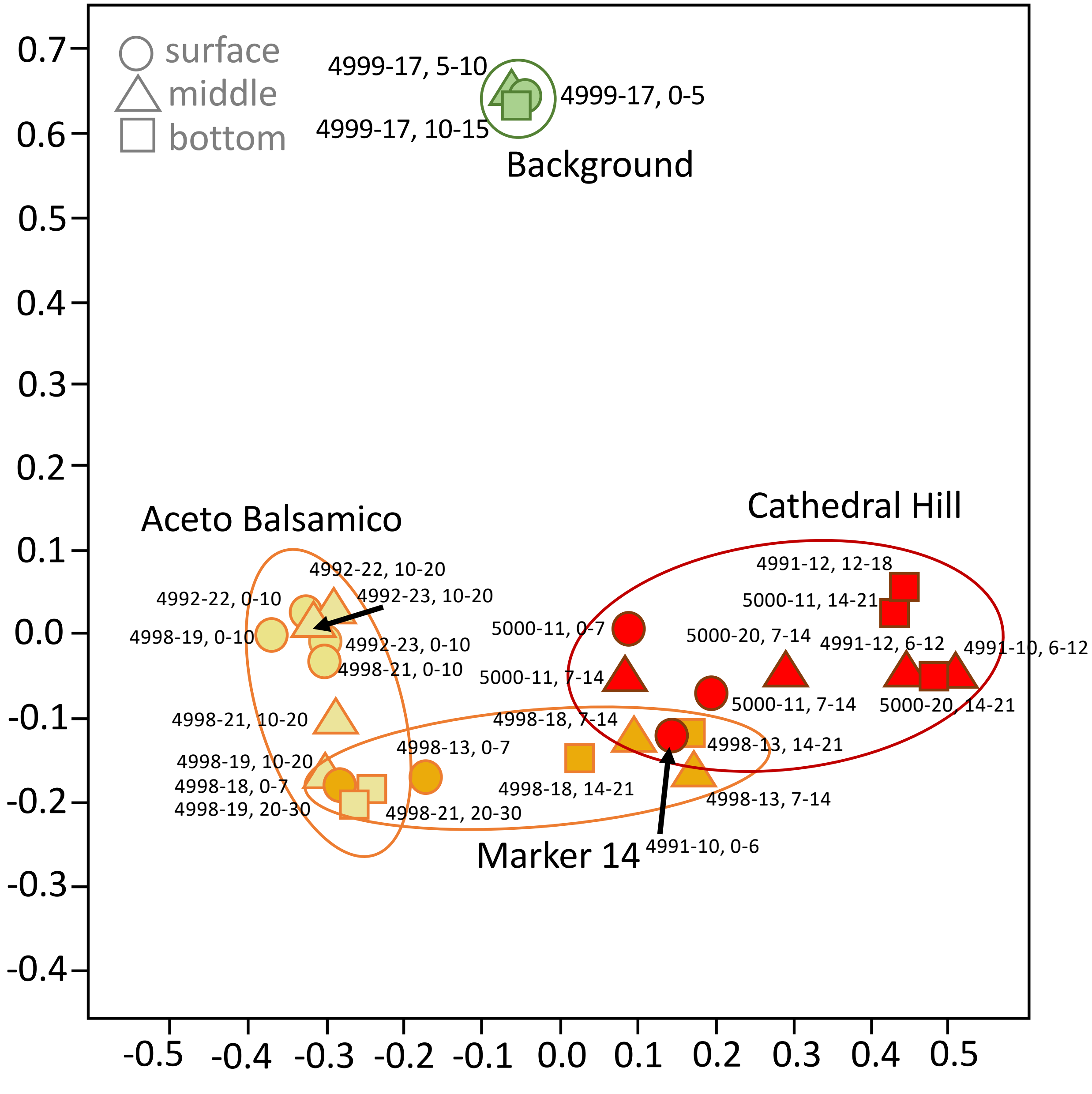

Supplementary Figure S7. PCoA plot of Bacterial and Archaeal sequences in Guaymas Basin sediments with background sediment excluded, and annotated by sediment core and depth interval in centimeters. The horizontal and vertical axis account for 27.8% and 12.7% of the dataset variance, respectively.

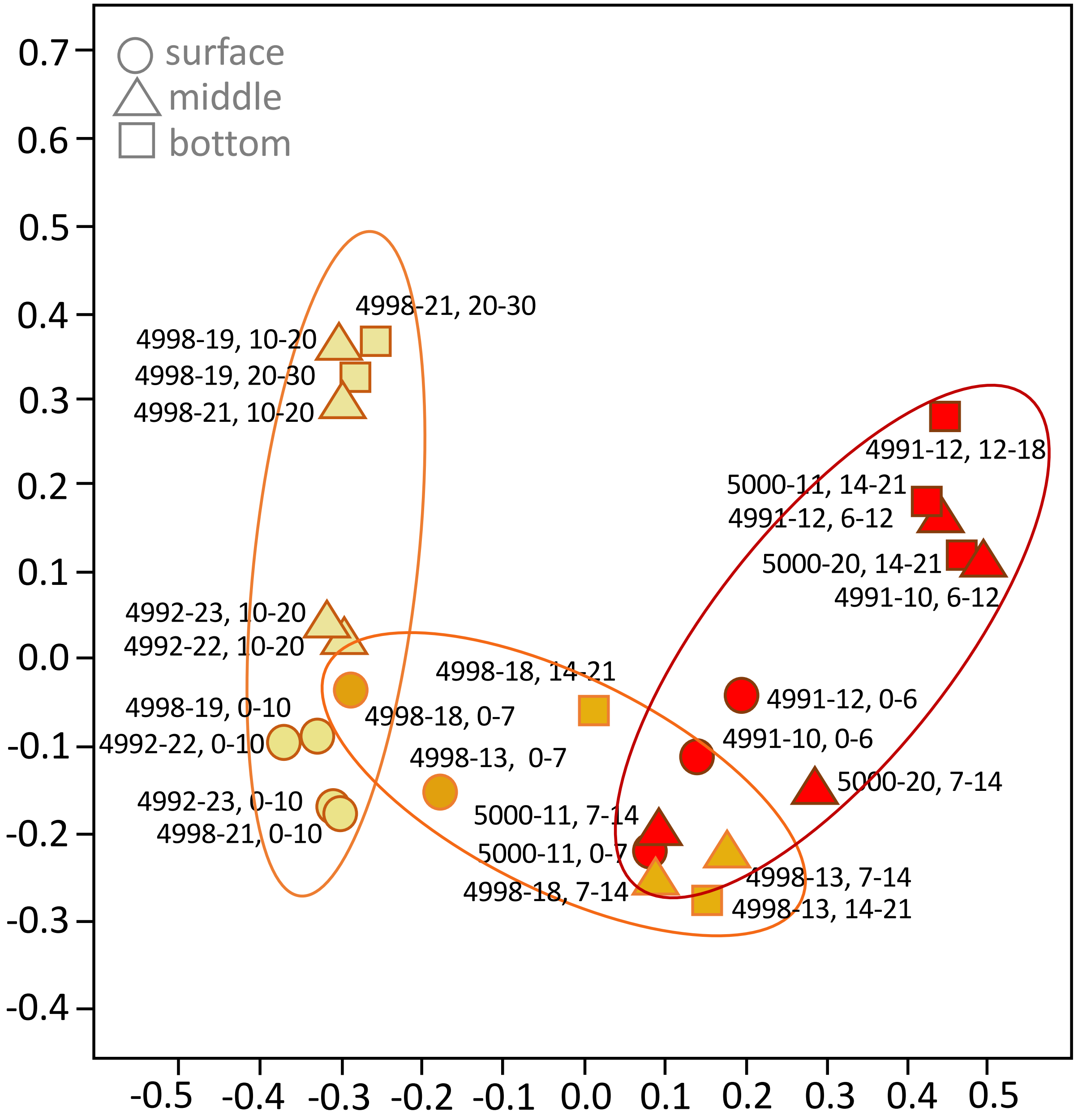

Supplementary Figure S8. PCoA plots for Bacterial and Archaeal sequences analyzed separately, and annotated by sampling site and depth intervals. The horizontal and vertical axis account for 30.6% and 14.3% of the bacterial dataset variance, and for 21.2% and 16.4% of the archaeal dataset variance, respectively.

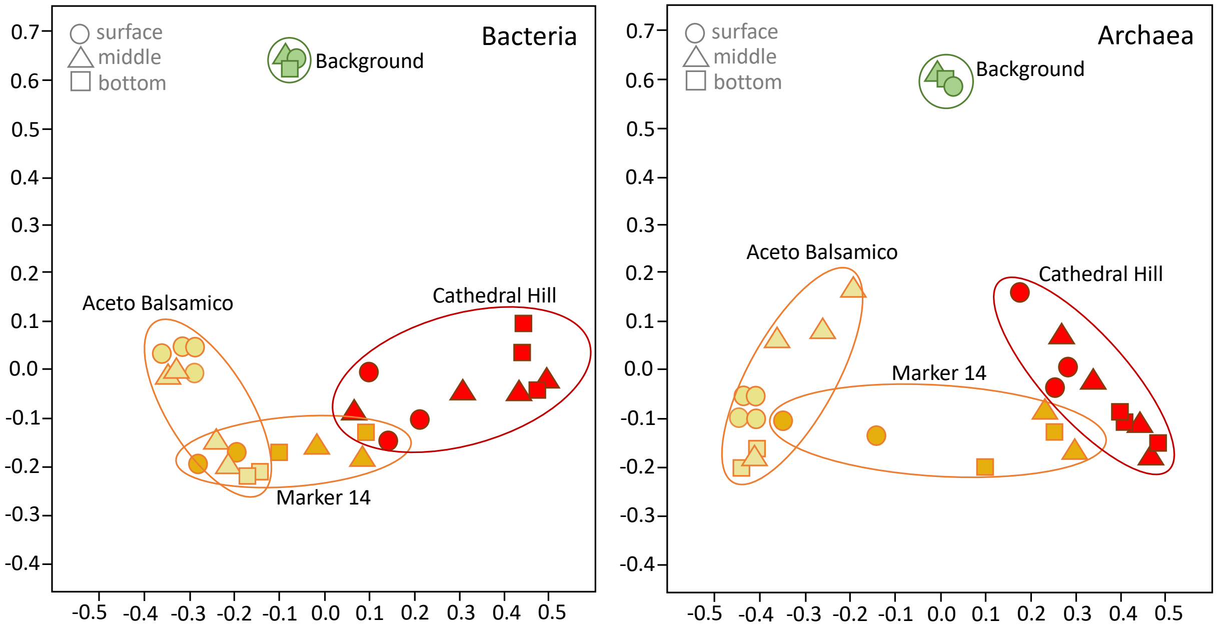

.

Supplementary Figure S9. Distance phylogeny of frequently occurring ASV sequences within the *Methanomicrobia*, until ASV 305. The branching pattern was checked by 1000 NJ Bootstrap iterations. The tree was rooted with ANME-1 as outgroup.

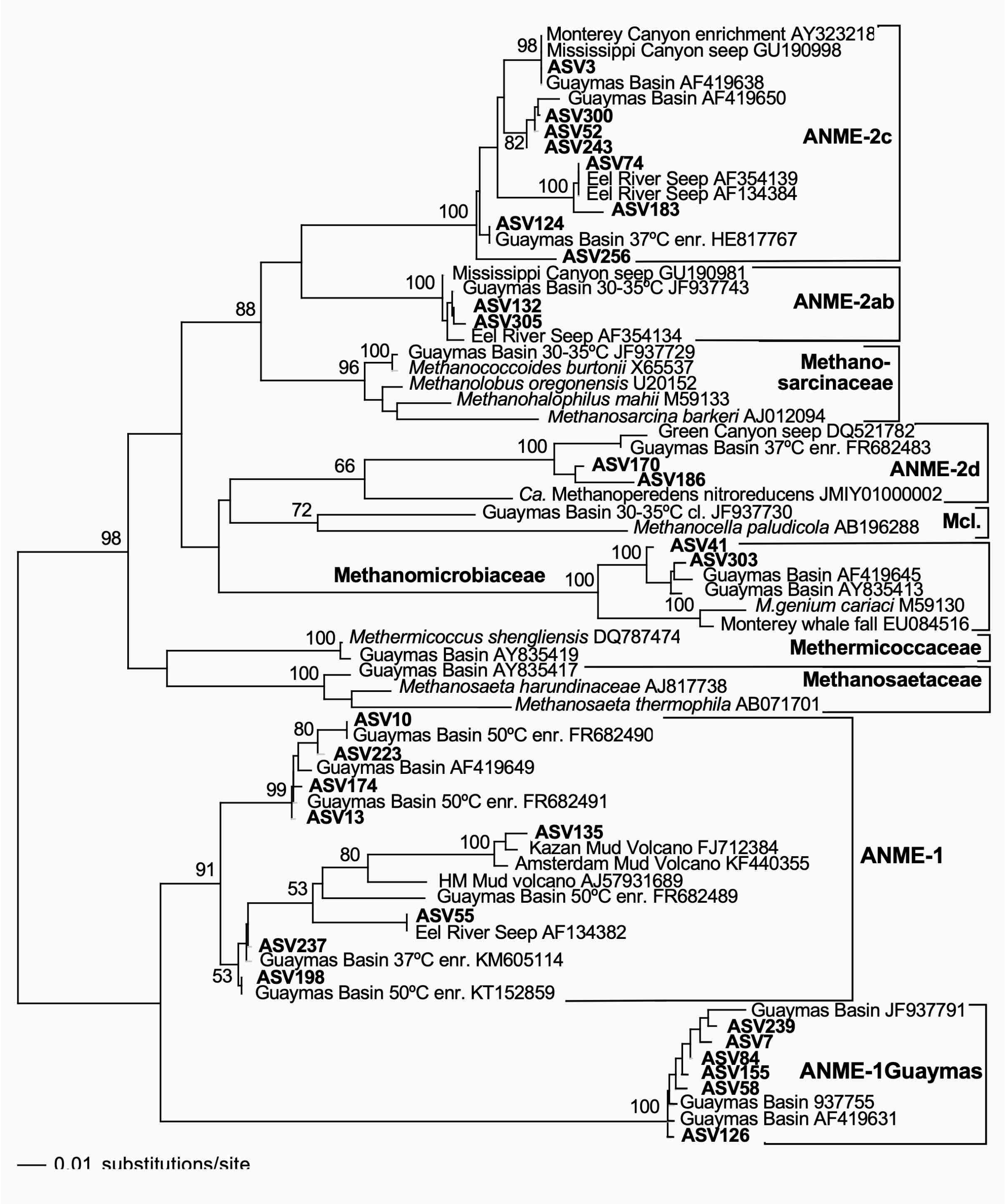

Supplementary Figure S10. Methane-cycling archaea occurrence**.** *Methanomicrobia* occurrence patterns are visualized as bubble plot of ASV frequencies in Background, Marker 14, Aceto Balsamico and Cathedral Hill sediment samples.

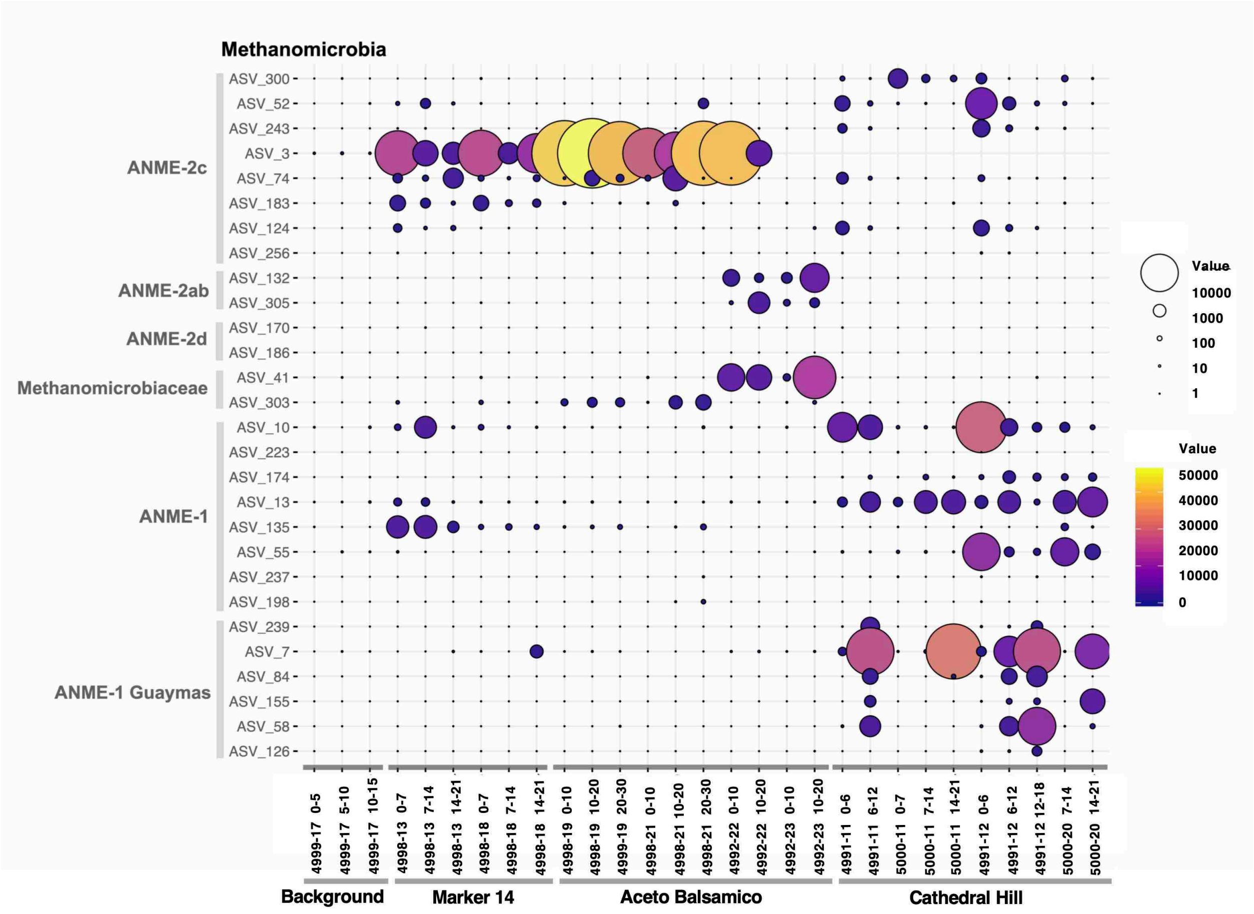

Supplementary Figure S11. Distance phylogeny of frequently occurring ASV sequences within the Deltaproteobacteria and *Desulfofervidales*, until ASV 301. The branching pattern was checked by 1000 NJ Bootstrap iterations. The tree was rooted with the *Desulfofervidales* as outgroup.

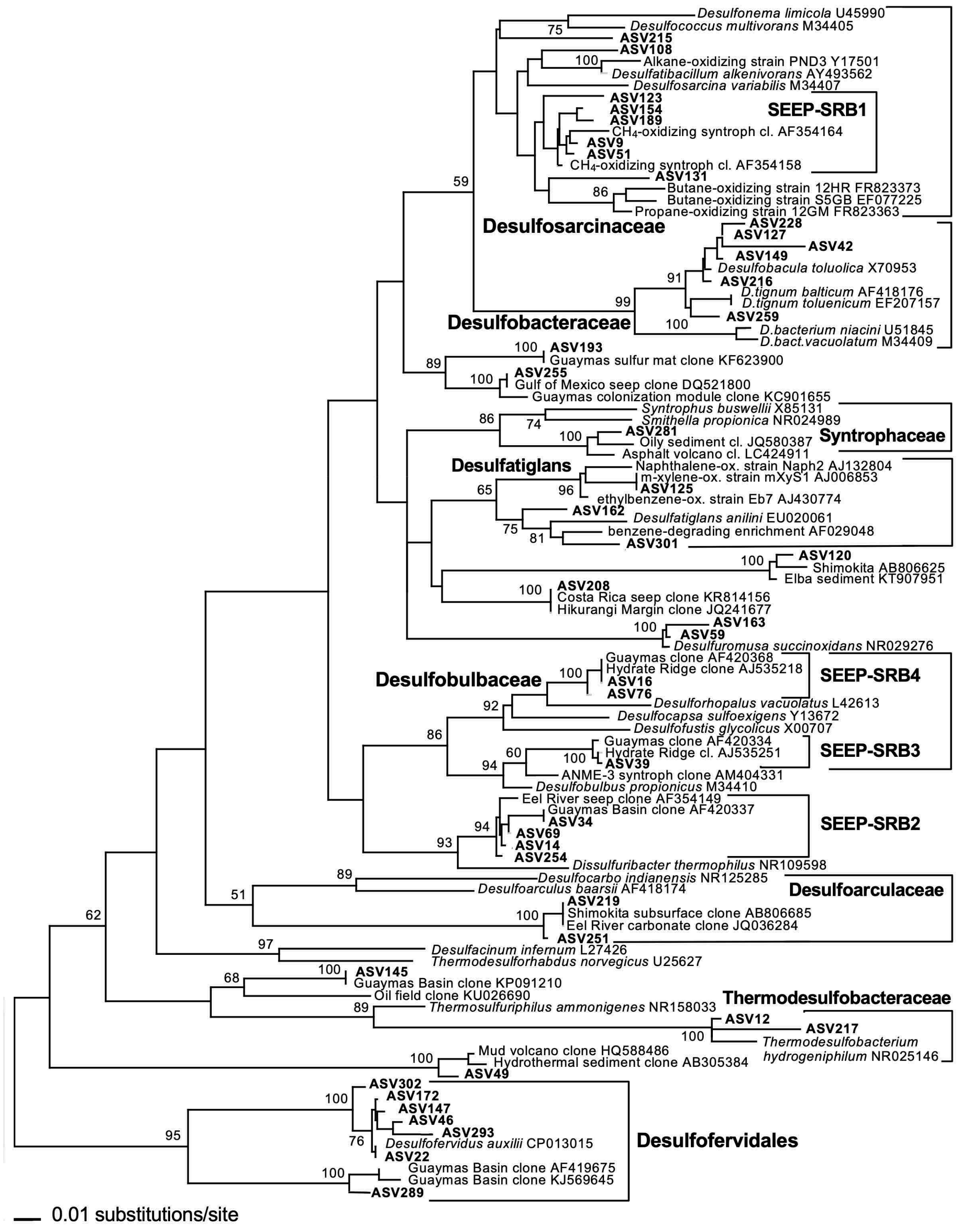

Supplementary Figure S12. Occurrence patterns of sulfate-reducing Deltaproteobacteria, *Thermodesulfobacteriaceae* and *Desulfofervidales* are visualized as bubble plot of ASV frequencies in Background, Marker 14, Aceto Balsamico and Cathedral Hill sediment samples.

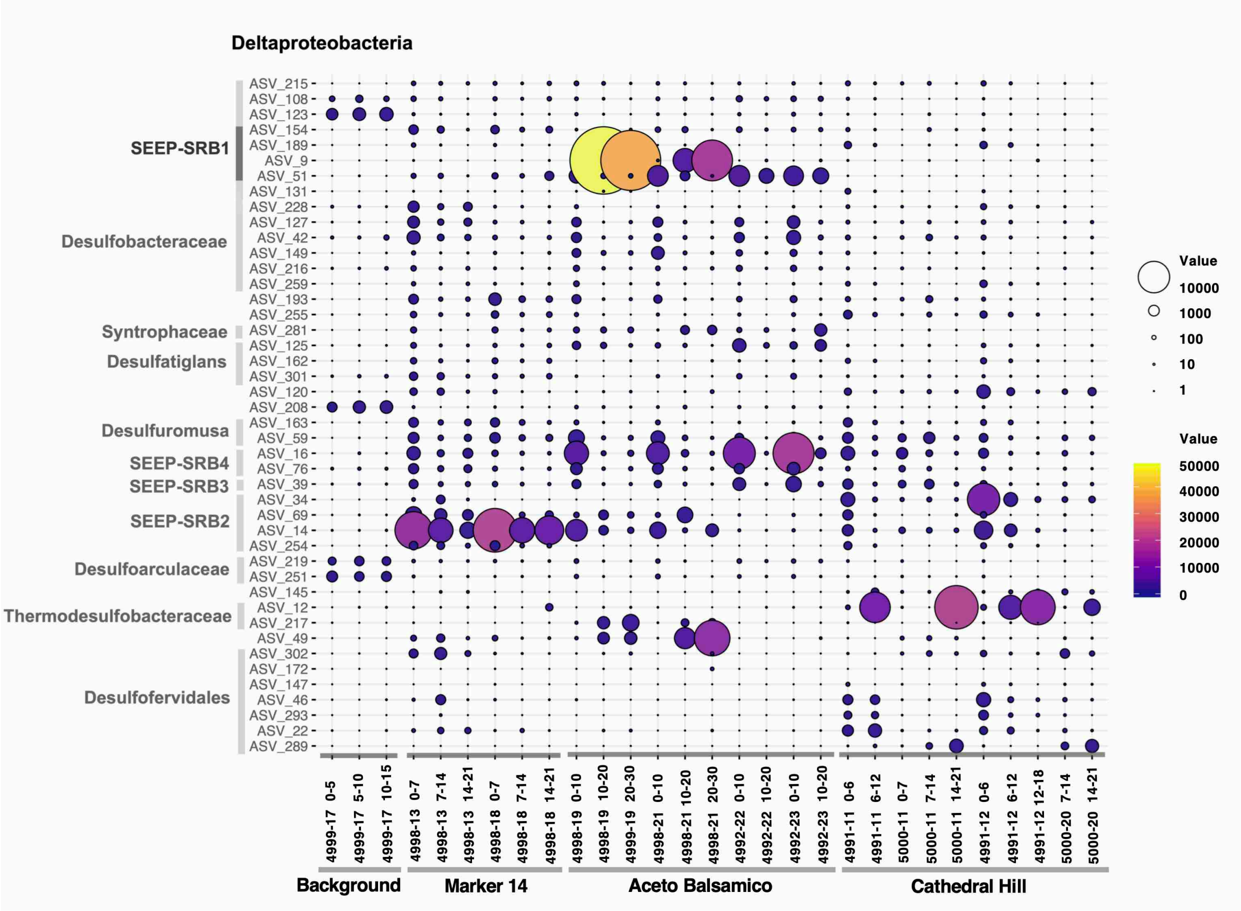

Supplementary Figure S13. Relative proportions of genus-level groups within the *Desulfobacteraceae*, and of methanogenic and methane-oxidizing orders within the *Methanomicrobia*, based on 16S rRNA gene sequence amplicons obtained from Guaymas Basin sediment samples (sum = 100%). The methane-cycling archaea were analyzed at the order level, since the SILVA 132 pipeline identified the ANME-1 archaea only at this level.

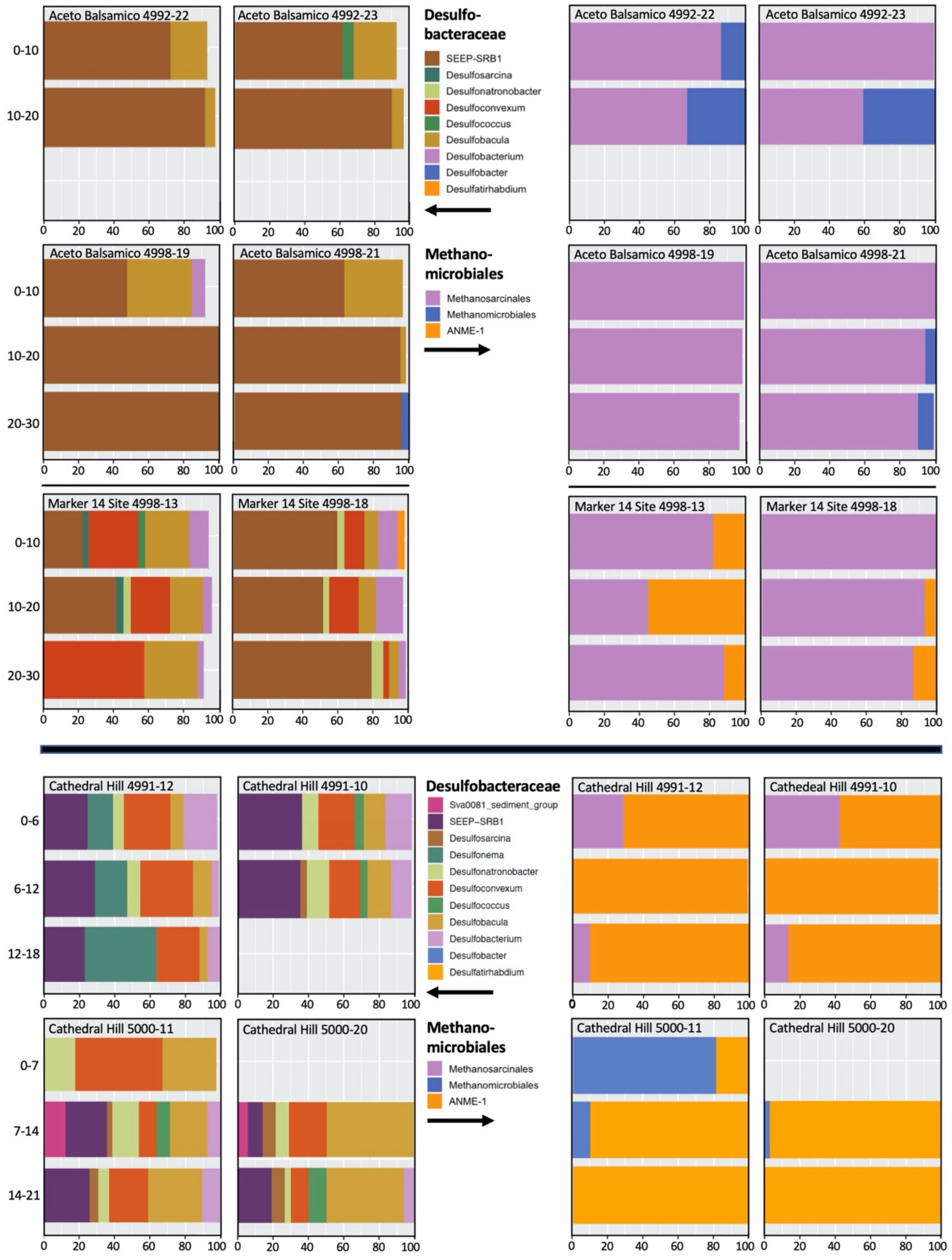

Supplementary Figure S14. PCoA analysis of fungal communities based on fungal iTag ASVs in Guaymas Basin samples as shown in Figure 10, and annotated by sediment core and depth interval in centimeters.

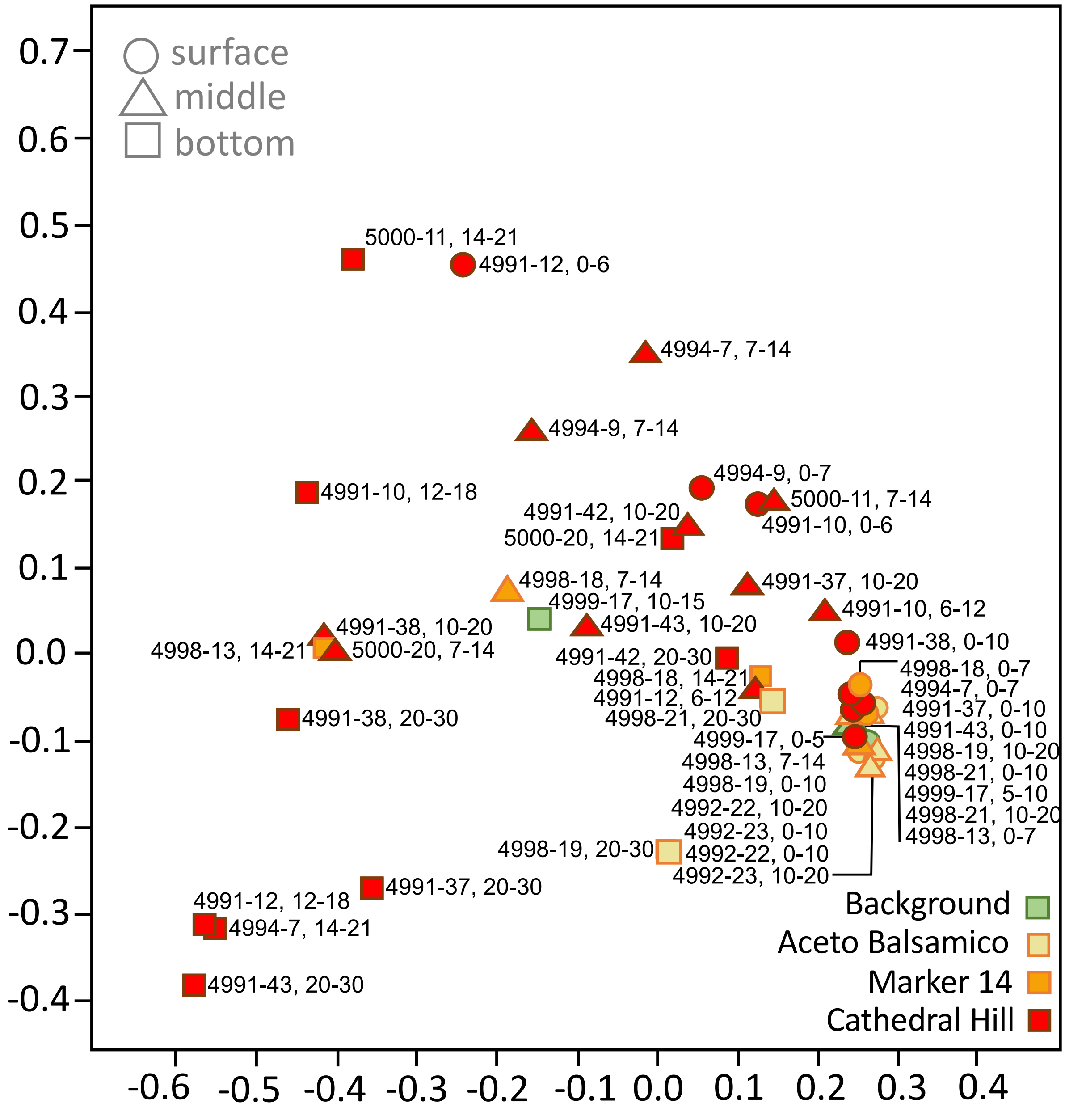

Supplementary Figure S15. Fungal α-diversity parameters (Shannon index, richness, evenness) correlations to the environmental parameters of sampling areas, in-situ temperature, sediment depth, and type of microbial mats.

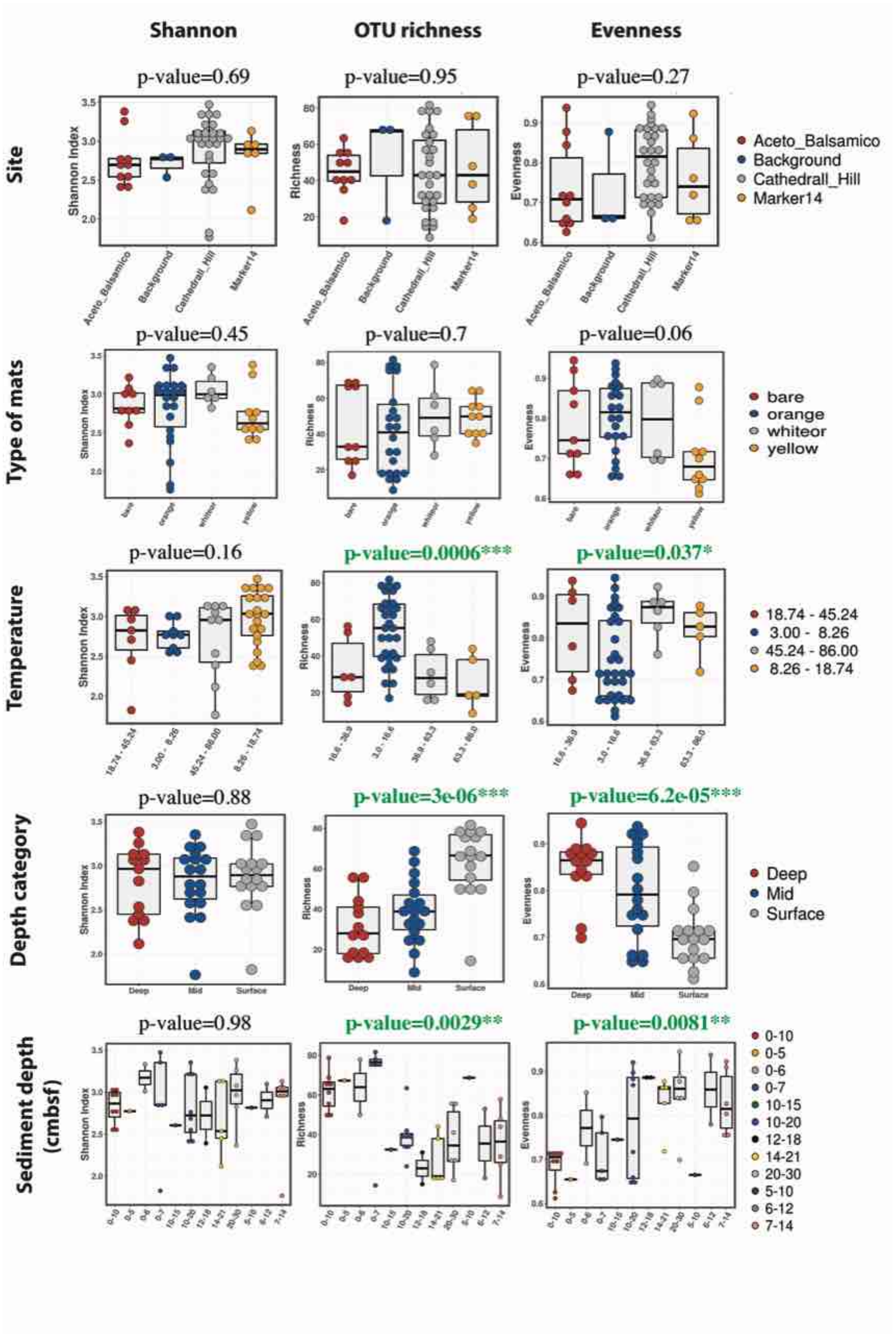

Supplementary Figure S16. Set of topological metrics considered relevant in the networking analysis of Guaymas hydrothermal sediment samples (see Figures 7 and 8).

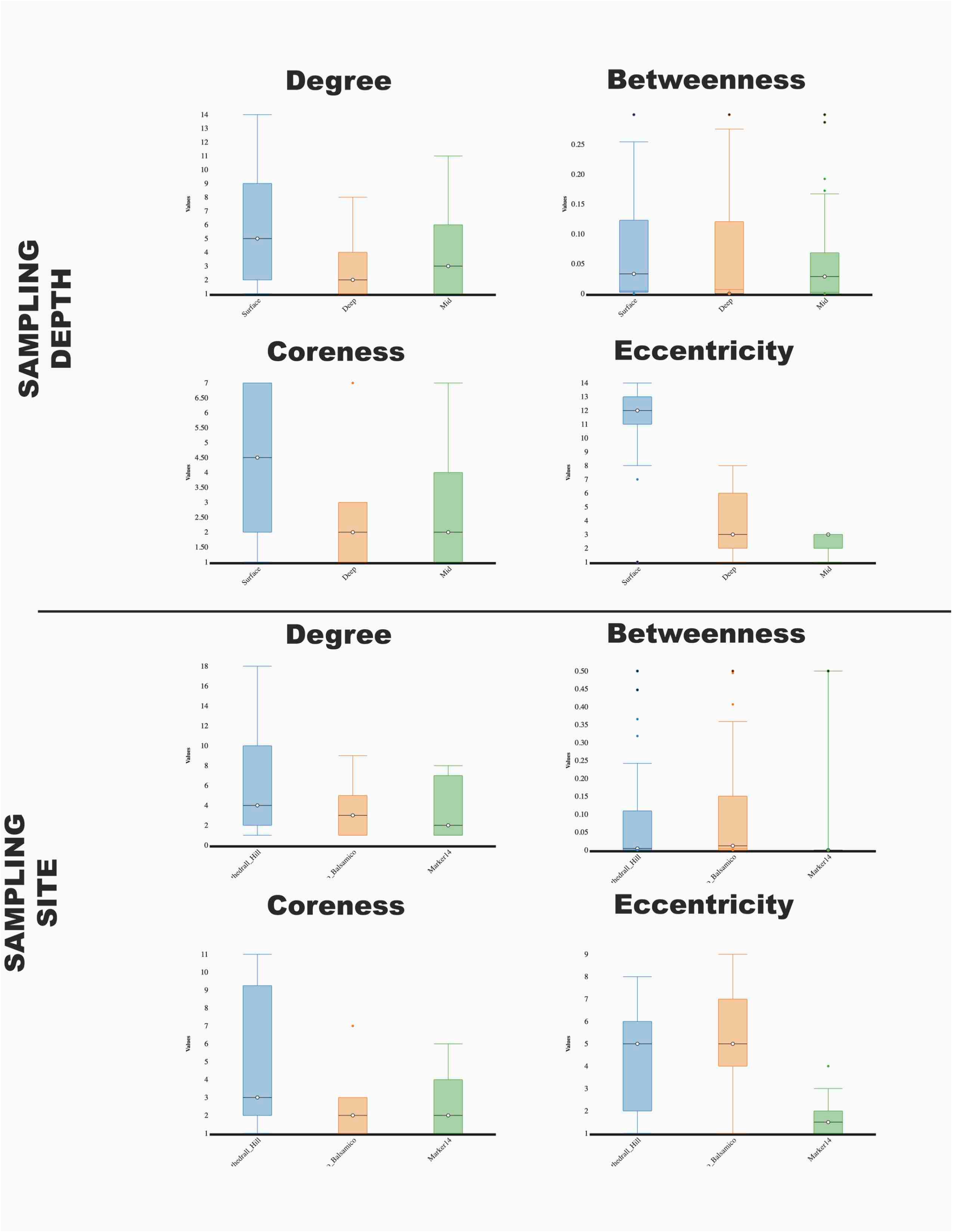
SAMPLING DEPTH
Degree
Betweenness
Coreness
Eccentricity
Values
Surface
Deep
Mid
SAMPLING SITE
Degree
Betweenness
Coreness
Eccentricity
Values
Marker14

Supplementary Table S1. Nutrient concentrations for rhizon porewater samples from Guaymas Basin sediment cores collected during expedition AT42-05 (MPI Bremen).

Core	Depth bsf [cm]	Ammonium [µM]	Nitrate [µM]	Nitrite [µM]	Phosphate [µM]	Silicate [µM]	Sulfate [mM]	Sulfide [mM]
4991-13	0.5	1879.6	179.2	13.7	16.4	702.2	18.27	1.81
	3	2454.1	8.0	0.2	4.0	292.7	17.65	2.74
	5	2144.5	0.0	0.2	3.0	175.9	20.88	3.80
	7.5	3045.9	0.0	0.1	3.4	781.5	16.45	3.06
	10	2582.1	0.0	0.1	2.3	487.4	15.29	3.06
	12	3084.8	0.0	0.0	1.6	1229.3	13.78	2.96
	14.5	3770.0	0.0	0.1	0.8	475.7	13.89	2.85
4991-17	1	1686.0	120.3	3.0	15.5	875.6	16.87	2.67
	3	2518.2	105.2	0.3	2.6	516.9	6.37	11.67
	6	3480.6	80.2	0.4	1.5	412.8	2.01	No data
	8	3891.3	0.0	0.2	0.9	557.3	1.80	10.86
	11	3745.7	0.0	0.1	0.5	677.0	5.21	10.52
	13.5	3736.6	0.0	0.1	0.6	837.7	10.22	7.49
4992-7	1	3461.6	0.0	0.2	5.2	405.7	1.83	2.17
	3.5	5594.3	0.0	0.4	5.0	316.6	0.42	2.87
	6.5	7113.0	0.0	0.3	5.5	303.4	0.55	1.22
	10	9008.5	0.0	0.1	1.9	283.0	0.42	0.39
	12.5	No data	0.0	No data	No data	No data	0.82	0.06
	15	10014.2	0.0	0.6	10.8	443.6	0.51	0.01
	17.5	No data	0.0	No data	No data	No data	No data	No data
	21	12143.3	0.0	No data	9.5	614.1	0.61	0.00
4999-15	1	17.6	0.0	0.2	4.7	131.8	29.37	0.00
	3.5	12.4	0.0	0.0	6.1	146.2	29.00	0.00
	5.5	11.8	0.0	0.0	1.4	136.5	29.66	0.00
	8	10.9	0.0	0.0	5.7	77.6	27.27	0.00
	10	10.5	0.0	0.0	5.2	100.9	30.26	0.00
	13	21.3	0.0	0.0	3.6	155.5	30.09	0.00
	15.5	159.5	0.0	0.0	20.1	111.7	30.82	0.00
5000-5	1	1005.2	32.3	0.8	67.4	114.5	28.48	2.09
	3.5	2817.2	160.6	No data	13.0	353.0	17.07	6.73
	5.5	2568.5	24.0	0.1	16.6	179.4	16.68	7.04
	8	3481.5	0.0	0.2	1.9	144.4	2.89	13.33
	11	4546.6	0.1	No data	0.9	124.1	No data	No data
	13.5	4619.7	0.0	0.5	1.9	869.8	1.73	13.37
	16.5	4010.8	0.0	0.5	2.9	127.7	2.94	14.06
5000-6	1	1932.6	0.0	2.0	12.1	524.8	22.53	3.23
	3.5	No data	0.0	No data	No data	No data	No data	No data
	6	6522.9	0.0	0.1	0.9	353.2	3.13	12.56
	8	4852.8	0.0	No data	0.5	423.7	1.93	10.21
	10.5	6826.2	0.0	No data	1.2	219.3	2.45	13.64
	12.5	5064.4	0.0	No data	1.5	558.5	6.94	8.54
	16	23677.9	0.0	No data	1.8	644.8	7.54	4.17
	18.5	36802.8	0.0	No data	0.8	904.5	10.15	3.78
5000-27	1	217.0	0.9	1.0	33.2	176.4	28.40	1.14
	2	427.1	0.4	0.9	23.8	124.4	25.14	2.01
	4.5	1607.0	0.0	0.2	8.2	285.4	17.33	4.92
	6.5	2127.0	0.0	0.1	2.2	223.0	12.31	9.09
	8.5	2470.9	0.3	No data	1.6	258.8	11.10	8.61
	10.5	4581.6	0.0	0.1	1.2	164.1	8.91	11.42

Supplementary Table S2. Sulfide and sulfate porewater data from sediment cores sliced in 3 cm intervals and centrifuged (UNC Chapel Hill).

Core & sampling area	Sediment depth	Sulfate [mM]	Sulfide [mM]
4991-40, Cathedral Hill	0-3 cm	No data	0.0
	3-6 cm	No data	0.0
	6-9 cm	No data	0.0
	9-12 cm	No data	0.0
	12-15 cm	No data	0.0
	15-18 cm	No data	No data
	18-21 cm	No data	0.034
	21-24 cm	No data	0.0
	24-27 cm	No data	0.0
4992-21, Aceto Balsamico	0-3 cm	No data	0.87
	3-6 cm	No data	1.45
	6-9 cm	No data	1.74
	9-12 cm	No data	0.36
	12-15 cm	No data	0.04
	15-18 cm	No data	0.0
4994-8, Cathedral Hill	0-3 cm	No data	0.99
	3-6 cm	No data	3.31
	6-9 cm	No data	1.97
	9-12 cm	No data	3.76
	12-15 cm	No data	2.59
	15-18 cm	No data	2.06
4998-17, Aceto Balsamico	3-6 cm	6.34	No data
	6-9 cm	12.32	3.63
	9-12 cm	No data	3.37
	12-15 cm	3.26	2.93
	15-18 cm	1.21	3.66
	18-21 cm	1.04	3.13
	21-24 cm	11.63	2.27
4998-24, Aceto Balsamico	0-3 cm	23.2	0.7
	3-6 cm	11.97	No data
	6-9 cm	3.98	2.96
	9-12 cm	0.54	2.37
	12-15 cm	8.49	2.35
	15-18 cm	2.46	1.85
	18-21 cm	10.54	1.54
	21-24 cm	0.46	1.58
	24-27 cm	2.80	1.26
	27-30 cm	7.99	1.13
4999-23, Background	0-3 cm	29.00	0.0
	3-6 cm	30.44	0.0
	9-12 cm	28.24	0.0
5000-13, Cathedral Hill	0-3 cm	25.99	2.89
	3-6 cm	15.96	3.72
	6-9 cm	9.55	2.58
	9-12 cm	5.13	2.46
	12-15 cm	5.53	1.10
	15-18 cm	8.81	0.48
	18-21 cm	11.64	2.92
5001-9, Cathedral Hill	0-3 cm	No data	1.77
	3-6 cm	No data	3.01
	6-9 cm	No data	3.27
	9-12 cm	No data	3.34
	12-15 cm	No data	2.05

Supplementary Table S3. Geochemical parameters of centrifuged porewater and matching sediment samples (WHOI).

Site	*Alvin* dive and core	Sample layer (cm)	NO_2+NO_3 (µM)	NH_4 (µM)	PO_4 (µM)	Dissolved Organic Carbon (mg/L)	Dissolved Inorganic Carbon (mg/L)	Dissolved Organic Nitrogen (mg/L)	% Total N	% Total C
Cathedral Hill	4991-10	0-6	2.41	2835	4.84	170	48.3	54.4	0.469	4.65
		6-12	1.08	2657	6.55	36.3	25.1	67.1	0.361	4.07
		12-18	0.356	2739	6.93	26.3	18.7	53.4	0.230	3.91
	4991-12	0-6	211	1865	12.7	32.5	49.5	54.1	0.278	2.99
		6-12	0.832	2301	4.06	107	29.7	29.8	0.141	2.32
		12-18	18.4	1892	7.43	85.4	21.4	65.4	0.135	1.93
	4991-37	0-10	2.08	18.2	5.72	44.9	29.1	0.960	0.329	3.58
		10-20	7.48	6.26	5.46	63.8	27.8	0.960	0.123	1.95
		20-30	2.37	4.93	5.28	76.0	26.9	0.960	0.078	1.44
	4991-38	0-10	2.19	163	5.90	12.2	27.4	1.68	0.315	3.58
		10-20	1.41	28.7	6.89	10.1	16.7	0.960	0.118	1.98
		20-30	0.970	27.1	3.90	21.0	30.2	1.55	0.091	1.82
	4991-42	0-10	1.01	1617	8.42	78.7	52.3	31.4	0.490	4.76
		10-20	1.28	1800	4.72	24.5	41.0	40.9	0.235	2.82
		20-30	1.63	1136	7.02	66.4	46.9	4.23	0.214	2.65
	4991-43	0-10	5.85	765	17.9	178	72.6	15.9	0.497	4.78
		10-20	0.000	770	10.4	25.0	63.2	17.6	0.290	3.23
		20-30	1.72	319	6.30	72.2	43.7	24.6	0.198	2.43
Aceto Balsamico	4992-22	0-10	5.00	2057	11.1	164	46.2	38.3	0.451	3.87
		10-20	2.40	3546	10.8	143	31.3	125	0.245	2.47
	4992-23	0-10	3.97	1200	13.0	160	59.1	43.3	0.473	3.87
		10-20	4.78	4100	12.1	94.4	26.9	111.4	0.253	2.47
Cathedral Hill	4994-07	0-7	125	696	11.3	177	47.2	19.9	0.582	5.07
		7-14	4.08	972	8.01	110	48.3	18.0	0.303	3.52
		14-21	5.84	707	6.86	1330	36.9	14.4	0.128	1.89
	4994-09	0-7	482	420	9.34	46.3	49.7	31.4	0.271	2.80
		7-14	127	645	7.26	14.7	50.2	7.50	0.159	2.26
Marker 14	4998-13	0-7	287	3596	32.7	222	69.2	71.9	0.449	3.64
		7-14	0.176	5720	10.8	148	39.0	106	0.254	2.29
		14-21	0.000	4728	8.63	68.2	26.6	120	0.184	1.89
	4998-18	0-7	260	3126	29.1	296	171	92.5	0.371	2.99
		7-14	0.628	6203	12.6	218	54.7	129	0.237	2.06
		14-21	0.222	6607	7.37	137	76.0	125	0.192	1.87
Aceto Balsamico	4998-19	0-10	0.494	2076	19.4	157	164	41.5	0.442	3.71
		10-20	0.906	4165	9.10	49.7	142	93.6	0.290	2.75
		20-30	0.000	2599	8.24	49.6	81.6	123	0.152	1.82
	4998-21	0-10	4.22	1284	19.0	190	162	39.0	0.483	4.11
		10-20	4.32	3251	9.27	48.9	192	97.2	0.257	2.54
		20-30	1.78	5040	7.21	110	109	124	0.167	1.78
Back-ground	4999-15	0-5	2.94	122	12.3	125	29.7	2.56	0.525	4.99
		5-10	2.59	67.8	17.6	78.0	31.6	2.75	0.503	4.83
		10-15	2.61	176	16.7	67.5	33.7	3.42	0.498	4.82
Cathedral Hill	5000-11	0-7	276	2225	17.9	62.4	100	56.5	0.488	4.10
		7-14	2.23	4523	4.39	83.7	110	73.5	0.232	2.40
		14-21	6.19	4841	7.29	96.6	61.3	72.2	0.135	1.58
	5000-20	0-7	3.87	4620	7.52	215	139	67.9	0.410	3.78
		7-14	25.5	2438	56.0	279	157	50.9	0.624	5.16
		14-21	1.98	3807	4.14	82.4	126	71.6	0.208	2.29

Supplementary Table S4. Geochemical trends for sampling areas and sediment depth, reflected in averages and standard deviations of DOC, DON, TOC, TON, ammonia and DIC contents for multiple cores grouped by sampling area, based on data in Table S3.

Samples and layers	DOC mg/L Av±stdv	DON mg/L Av±stdv	TOC w% Av±stdv	TON w% Av±stdv	NH4 µM Av±stdv	DIC mg/L Av±stdv
Cathedral Hill hydrothermal cores 4991-10,12, 42,43; 4994-07,09; 5000-11, 20						
top	120±73	42.8±18.4	4.12±0.86	0.44±0.11	1880±1383	69.8±33.4
Middle	85±87	34.3±26.5	3.23±1.01	0.29±0.15	2013±1283	65.6±45.4
bottom	71.5±24.5	47.3±24.9	2.38±0.76	0.18±0.04	2202±1676	50.7±36.3
all	94.1±65.5	58.3±23	3.28±1.11	0.31±0.15	2024.5±1384	62.5±37.9
Aceto Balsamico hydrothermal cores 4992-22,23; 4998-19,21						
top	168±15.1	40.5±2.3	3.90±0.16	0.46±0.02	1654±477	107.8±63.9
middle	84±44.7	106.8±14.4	2.56±0.13	0.26±0.02	3766±441	98±82.2
bottom	79.8±42.7	123.5±0.7	1.8±0.02	0.16±0.01	3820±1726	95.3±19.4
all	116.7±53.7	83.6±38.6	2.93±0.88	0.32±0.13	2932±1297	101.4±60.7
Marker 14 hydrothermal cores 4998-13,18						
top	259±52.3	82.2±14.6	3.31±0.46	0.41±0.05	3361±332	120.1±72
Middle	183±49.5	117.5±16.3	2.17±0.16	0.25±0.01	5962±341	46.8±11.1
bottom	102.6±48.6	122.5±3.5	1.88±0.01	0.19±0.01	5668±1329	51.3±34.9
all	181.5±80	107.4±22	2.46±0.71	0.28±0.11	4997±1422	72.7±51.5
Cold Background core 4999-15						
top	125	2.55	4.99	0.52	122	29.7
middle	78	2.75	4.83	0.50	68	31.6
bottom	67.5	3.4	4.82	0.50	176	33.7
all	90.2±30.6	2.9±0.45	4.88±0.09	0.50±0.01	122±54	31.7±2
Cool, bare sediment Cathedral Hill cores 4991-37,38						
top	28.6±23.1	1.32±0.5	3.58±0	0.32±0.01	91±101	28.2±1.2
middle	36.9±38	0.96±0.0	1.96±0.02	0.12±0.003	17.5±15.9	22.2±7.8
bottom	48.5±38.9	1.25±0.42	1.63±0.27	0.08±0.01	16.0±15.7	28.5±2.3
all	39±27.9	1.18±0.34	2.39±0.94	0.18±0.11	41.4±60.4	26.3±4.9

Supplementary Table S5. Porewater methane $\delta^{13}C$ CH_4 values for Guaymas Basin sediment cores collected during expedition AT42-05.

Site	*Alvin* dive and core	Sample layer (cm)	$\delta^{13}C$ CH_4 (‰)	1σ (‰)
Cathedral Hill	4991-7	0-6	-34.64	0.19
		6-12	-34.61	0.39
		12-18	-38.13	0.45
	4991-35	0-10	-46.30	0.02
		10-20	-45.45	0.23
	4991-41	0-10	-37.23	0.13
		10-20	-37.17	0.39
		20-30	-29.02	0.04
	4994-5	0-7	-30.64	0.08
		7-14	-26.04	0.08
		14-21	-24.54	0.17
	5000-8	0-7	-31.85	0.07
		7-14	-32.77	0.08
		14-21	-33.53	0.08
Aceto Balsamico	4992-24	0-10	-47.09	0.03
		10-20	-52.30	0.70
	4998-20	0-10	-53.88	0.17
		10-20	-53.52	0.27
		20-30	-52.40	0.12
Marker 14	4998-15	0-7	-37.13	0.05
		7-14	-38.95	0.20
		14-21	-39.91	0.03
Background	4999-8	0-7	-70.15	1.41
		7-14	-63.88	3.88
		14-21	-64.39	1.24

Supplementary Table S6. ASV numbers and averages for Guaymas Basin sediment samples.

Sampling site & core	Sediment layer	ASV count	ASV diversity (av.) by layer	ASV diversity (av.) by site
**Aceto Balsamico**				
4992-22	0-10 cm	1583	1495	1233
4992-23	0-10 cm	1401		
4998-19	0-10 cm	1901		
4998-21	0-10 cm	1096		
4992-22	10-20 cm	798	1,093	
4992-23	10-20 cm	1156		
4998-19	10-20 cm	1001		
4998-21	10-20 cm	1418		
4998-19	20-30cm	958	989	
4998-21	20-30 cm	1019		
**Background**				
4999-17	0-5 cm	2662	2662	2469
4999-17	5-10 cm	2450	2450	
4999-17	10-15 cm	2294	2294	
**Cathedral Hill**				
4991-10	0-6 cm	1956	1756	1183
4991-12	0-6 cm	2110		
5000-11	0-7 cm	1201		
5000-20	0-7 cm	excluded		
4991-10	6-12 cm	1296	1078	
4991-12	6-12 cm	1138		
5000-11	7-14 cm	750		
5000-20	7-14 cm	1128		
4991-10	12-18 cm	No data	716	
4991-12	12-18 cm	664		
5000-11	14-21 cm	609		
5000-20	14-21 cm	876		
**Marker 14**				
4998-13	0-7 cm	2376	1962	1526
4998-18	0-7 cm	1548		
4998-13	7-14 cm	1491	1234	
4998-18	7-14 cm	977		
4998-13	14-21 cm	1662	1382	
4998-18	14-21 cm	1102		

Supplementary Table S7. ASV sequences used for Methanomicrobial and Deltaproteobacterial phylogenies (Figures S9 and S11).

Methanomicrobia

ASV_3
ACCGGCGGCCCGAGTGGTAGCCGCTATTATTGGGTCTAAAGGGTCCGTAGCCGGTTC
GATAAGTCCTCTGGGAAATCTGGTTGCTTAACAATCAGACTGCCAAGGGATACTGTC
GAACTTGAGACCGGGAGAGGTAAGAGGTACTTCAGGGGTAGGAGTGAAATCTTGTA
ATCCCTGGGGGACCATCTGTGGCGAAGGCGTCTTACCAGAACGGGTCTGACGGTGA
GGGACGAAAGCTGGGGGCGCAAACCGG

ASV_7
ACCGGCAGCCCGAGTGGTGGCCGCGTTTATTGGGCCTAAAGGGTCCGTAGCCGGTC
GGGTAAGTTCCTCGGGAAACCTCGCCGCTCAACGGTGAGGCTTCCGGGGAATACTG
CCCGACTTGGGACCGGGATGGGCCGGAGGTACTCCCGGGGTAGGGGTGAAATCCGT
TGATCCCGGGAGGACCACCCGTAGCGAAGGCGTCCGGCTGGAACGGGTCCGACGGT
GAGGGACGAAGGCCAGGGGCGCGAACCGG

ASV_10
ACCGGCAGCCCAAGTGGTAGCCACTTTTATTGGGTCTAAAGCGTCCGTAGCCGGTTG
AGTGAGTTCCTTGGGAAATTTGGCTGCTTAACAGTCAAACTTCCAGGGAATACTGCT
CGACTTGGGACTGGGAGAGGTCAGAGGTACTCCAGGGGTAGGGGTGAAATCCATTA
ATCCTTGGGGGACCACCGGTAGCGAAGGCGTCTGACCAGACCAGGTCCGACGGTGA
GGGACGAAGGCTAGGGTCGCGAACCGG

ASV_13
ACCGGCAGCCCAAGTGGTAGCCACTTTTATTGGGTCTAAAGCGTCCGTAGCCGGTTG
AGTAAGTTCCTTGGGAAATTTGGCTGCTTAACAGTCAAGCTTCCAGGGAATACTGCT
CGACTTGGGACTGGGAGAGGTCAGAGGTACTCCAGGGGTAGGGGTGAAATCCGTTA
ATCCCTGGGGGACCACCGGTAGCGAAGGCGTCTGACCAGACCAGGTCCGACGGTGA
GGGACGAAGGCTAGGGTCGCGAACCGG

ASV_41
ACCGGCGGCTCGAGTGGTGGCCACTTTTATTGGGCTTAAAGCGTCCGTAGCTGGATT
GATAAGTCTCCTGGGAAATCCGCCGGCTTAACCGGTGGGCGTCCGGGAGATACTGTC
AATCTAGGGACCGGGAGAGGTGAGAGGTACTCCGGGGGTAGGAGTGAAATCCTGTA
ATCCCCGGGGGACCACCTGTGGCGAAGGCGTCTCACCAGAACGGCTCCGACAGTGA
GGGACGAAAGCTGGGGGAGCGAACCGG

ASV_52
ACCGGCGGCCCGAGTGGTAGCCACTATTATTGGGTCTAAAGGGTCCGTAGCCGGTTT
GGTAAGTCCTCTGGAAAATCTGGTTGCTCAACAATCAGACTGCCAAGGGATACTGTC
GAACTTGAGACCGGGAGAGGTAAGAGGTACTTCAGGGGTAGGAGTGAAATCTTGTA
ATCCCTGGGGGACCATCTGTGGCGAAGGCGTCTTACCAGAACGGGTCTGACGGTGA

GGGACGAAAGCTGGGGGCGCGAACCGG

ASV_55
ACCGGCAGCTCAAGTGGTAGCCATGCTTATTGGGTCTAAAGCGTCCGTAGCCGGCTG
AGTAAGTTCCTTGGGAAATTTGACCGCTTAACGGTTAAGCTATCAGGGAATACTGCT
TGGCTTGGGACCGGGAGAGGTCAGAGGTACTCCAGGGGTAGGGGTGAAATCTATTA
ATCCTTGGGGGACCACCGGTAGCGAAGGCGTCTGACCAGACCGGGTCCGACGGTGA
GGGACGAAGGCTGGGGTCGCGAACCGG

ASV_58
ACCGGCAGCCCGAGTGGTGGCCGCGTTTATTGGGCCTAAAGGGTCCGTAGCCGGTC
GGGTAAGTTCCTCGGGAAACCTCGCCGCTCAACGGTGAGGCTTCCGGGGAATACTG
CCCGACTTGGGACCGGGAGGGGCCGGAGGTACTCCCGGGGTAGGGGTGAAATCCGT
TGATCCCGGGAGGACCACCCGTAGCGAAGGCGTCCGGCTGGAACGGGTCCGACGGT
GAGGGACGAAGGCCAGGGGCGCGAACCGG

ASV_74
ACCGGCGGCCCGAGTGGTAGCCGCTATTATTGGGTCTAAAGGGTCCGTAGCCGGTTT
GATAAGTCCTCTGGGAAATCTGATTGCTCAACAATCAGGCTGCCAAGGGATACTGTC
AAACTTGAGACCGGGAGAGGTAAGAGGTACTTCAAGGGTAGGAGTGAAATCTTATA
ATCCTTGGGGGACCGTCTGTGGCGAAGGCGTCTTACCAGAACGGGTCTGACGGTGA
GGGACGAAAGCTGGGGGCGCGAACCGG

ASV_84
ACCGGCAGCCCGAGTGGTGGCCGCGTTTATTGGGCCTAAAGGGTCCGTAGCCGGTC
GGGTAAGTTCCTCGGGAAACCTCGCCGCTCAACGGTGAGGCTTCCGGGGAATACTG
CCTGACTTGGGACCGGGATGGGCCGGAGGTACTCCCGGGGTAGGGGTGAAATCCGT
TGATCCCGGGAGGACCACCCGTAGCGAAGGCGTCCGGCTGGAACGGGTCCGACGGT
GAGGGACGAAGGCCAGGGGCGCGAACCGG

ASV_124
ACCGGCGGCCCGAGTGGTAGCCGCTATTATTGGGTCTAAAGGGTCCGTAGCCGGTTC
GGTAAGTCCTCTGGGAAATCTGGTTGCTTAACAATCAGGCTGCCAAGGGATACTGCC
GAACTTGAGACCGGGAGAGGTAAGAGGTACTTCAGGGGTAGGAGTGAAATCTTGTA
ATCCCTGGGGGACCGTCTGTGGCGAAGGCGTCTTACCAGAACGGGTCTGACGGTGA
GGGACGAAAGCTGGGGGCGCGAACCGG

ASV_126
ACCGGCAGCCCGAGTGGTGGCCGCGTTTATTGGGCCTAAAGGGTCCGTAGCCGGTC
GGGTAAGTTCCTCGGGAAACCTCGCCGCTCAACGGTGAGGCTTCCGGGGAATACTG
CCTGACTTGGGACCGGGAGGGGCCGGAGGTACTCCCGGGGTAGGGGTGAAATCCGT
TGATCCCGGGAGGACCACCCGTAGCGAAGGCGTCCGGCTGGAACGGGTCCGACGGT
GAGGGACGAAGGCCAGGGGCGCGAACCGG

ASV_132
ACCGGCGGCCCGAGTGGTAGCCGTTTTTATTGGGTCTAAAGGGTCCGTAGCCGGCCT

GATAAGTCCTTTGGGAAATCTGGCAGCTTAACTGTCAGGCTTCTAAGGGATACTGTC
AGGCTTGGGACCGGAAGAGGTAAGGGGTACTCCAGGGGTAGGAGTGAAATCTTGTA
ATCCCTGGGGGACCATCTGTGGCGAAGGCGCCTTACCAGAACGGGTCCGACGGTGA
GGGACGAAAGCTAGGGGAGCAAACCGG

ASV_135
ACCGGCAGCCCAAGTGGTGGCCACTTTTACTGGGTCTAAAGCGTCCGTAGCCGGCTA
GGTAAGTTCCTTGGGAAATTTGATCGCTTAACGGTTAAGCTTTCAGGGAATACTGCT
TGGCTTGGGACCGGGAGAGGTCAGAGGTACCTCAAGGGTAGGGGTGAAATCCGTTG
ATCCTTGGGGGACCGCCAGTAGCGAAGGCGTCTGACCAGACCGGATCCGACGGTGA
GGGACGAAGGCTAGGGTAGCGAACCGG

ASV_155
ACCGGCAGCCCGAGTGGTGGCCGCGTTTATTGGGCCTAAAGGGTCCGTAGCCGGTC
GGGTAAGTTCCTCGGGAAACCTCGCTGCTCAACGGTGAGGCTTCCGGGGAATACTGC
CCGACTTGGGACCGGGATGGGCCGGAGGTACTCCCGGGGTAGGGGTGAAATCCGTT
GATCCCGGGAGGACCACCCGTAGCGAAGGCGTCCGGCTGGAACGGGTCCGACGGTG
AGGGACGAAGGCCAGGGGCGCGAACCGG

ASV_170
ACCGGCGGCCCGAGTGGTAACCGATTTTATTGGGCTTAAAGCGTTCGTAGCCGGTTT
GGTAAGTCTCTTAGGAAATCTGGCAGCTTAACTGCTAGGCGTCTAAGAGATACTGCC
AAACTTGGGACCGGGAGAGGTAGGAGGTACTCCATGGGTAGGGGTGAAATCTTGTA
ATCCTTGGAGGACCATCGATGGCGAAGGCATCCTACCAGAACGGGTCCGACGGTGA
GGGACGAAAGCTGGGGGCACAAACCGG

ASV_174
ACCGGCAGCCCAAGTGGTAGCCACTTTTATTGGGTCTAAAGCGTCCGTAGCCGGTCG
AGTAAGTTCCTTGGGAAATTTGGCTGCTTAACAGTCAAGCTTCCAGGGAATACTGCT
CGACTTGGGACTGGGAGAGGTCAGAGGTACTCCAGGGGTAGGGGTGAAATCCGTTA
ATCCCTGGGGGACCACCGGTAGCGAAGGCGTCTGACCAGACCAGGTCCGACGGTGA
GGGACGAAGGCTAGGGTCGCGAACCGG

ASV_183
ACCGGCGGCCCGAGTGGTAGTCGCTATTATTGGGTCTAAAGGGTCCGTAGCCGGTTT
GATAAGTCTTCTGGGAAATCTGATTGCTCAACAATCAGGCTGCCAAGGGATACTGTC
AAACTTGAGACCGGGAGAGGTAAGAGGTACTTCAGGGGTAGGAGTGAAATCTTATA
ATCCTTGGGGGACCGTCTGTGGCGAAGGCGTCTTACCAGAACGGGTCTGACGGTGA
GGGACGAAAGCTGGGGGCGCGAACCGG

ASV_186
ACCGGCGGCCCGAGTGGTAACCGACTTTATTGGGCTTAAAGCGTTCGTAGCCGGTTT
GGTAAGTCTCTTAGGAAATCTGGCAGCTTAACTGTTAGGCGTCTAAGAGATACTGCT
AAACTTGGGATCGGGAGAGGTAGGAGGTACTCCAGGGGTAGGGGTGAAATCTTGTA
ATCCTTGGAGGACCATCGATGGCGAAGGCATCCTACCAGAACGAGTCCGACGGTGA
GGGACGAAAGCTGGGGGCACAAACCGG

ASV_198
ACCGGCAGCCCAAGTGGTAGCCACGTTTATTGGGTCTAAAGCGTCCGTAGCCGGCTG
GGTAAGTTCCTTGGGAAATTTGGCTGCTTAACAGTCAAGCTTTCAGGGAATACTGCC
CGGCTTGGGACCGGGAGAGGTCAGAGGTACTCCAGGGGTAGGGGTGAAATCCTTTA
ATCCTTGGGGGACCACCGGTAGCGAAGGCGTCTGACCAGACCGGGTCCGACGGTGA
GGGACGAAGGCTAGGGGCGCGAACCGG

ASV_223
ACCGGCAGCCCAAGTGGTAGCCACTTTTATTGGGTCTAAAGCGTCCGTAGCCGGTTG
AGTAAGTTCCTTGGGAAATTTGGCTGCTTAACAGTCAAACTTCCAGGGAATACTGCT
CGACTTGGGACTGGGAGAGGTCAGAGGTACTCCAGGGGTAGGGGTGAAATCCATTA
ATCCCTGGGGGACCACCGGTAGCGAAGGCGTCTGACCAGACCAGGTCCGACGGTGA
GGGACGAAGGCTAGGGTCGCGAACCGG

ASV_237
ACCGGCAGCCCAAGTGGTAGCCACGTTTATTGGGTCTAAAGCGTCCGTAGCCGGCTG
GGTAAGTTCCTTGGGAAATTTGGCTGCTTAACAGTCAAGCTTTCAGGGAATACTGCC
TGGCTTGGGACCGGGAGAGGTCAGAGGTACTCCAGGGGTAGGGGTGAAATCCTTTA
ATCCTTGGGGGACCACCGGTAGCGAAGGCGTCTGACCAGACCGGGTCCGACGGTGA
GGGACGAAGGCTAGGGGCGCGAACCGG

ASV_239
ACCGGCAGCCCGAGTGGTGGCCGCGTTTATTGGGCCTAAAGGGTCCGTAGCCGGTC
GGGTAAGTTCCTCGGGAAACCTCGCCGCTCAACGGTGAGGCTTCTGGGGAATACTGC
CCGACTTGGGACCGGGATGGGCCGGAGGTACTCCCGGGGTAGGGGTGAAATCCGTT
GATCCCGGGAGGACCACCCGTAGCGAAGGCGTCCGGCTGGAACGGGTCCGACGGTG
AGGGACGAAGGCCAGGGGCGCGAACCGG

ASV_243
ACCGGCGGCCCGAGTGGTAGCCACTATTATTGGGTCTAAAGGGTCCGTAGCCGGTTT
GGTAAGTCCTCTGGAAAATCTGGTTGCTCAACAATCAGACTGCCAAGGGATACTGTC
AAACTTGAGACCGGGAGAGGTAAGAGGTACTTCAGGGGTAGGAGTGAAATCTTGTA
ATCCCTGGGGGACCATCTGTGGCGAAGGCGTCTTACCAGAACGGGTCTGACGGTGA
GGGACGAAAGCTGGGGGCGCGAACCGG

ASV_256
ACCGGCGGCCCGAGTGGTAACCGCTATTATTGGGTCTAAAGGGTCCGTAGCCGGTTC
GGTAAGTCCTTTGGGAAATCTGGTTGCTTAACAATTAGGCTGCTAAGGGATACTGTC
GAACTTGAGACCGGGAGAGGTAAGAGGTACTTCAGGGGTAGGAGTGAAATCTTGTA
ATCCTTGGGGGACCATCTGTGGCGAAGGCGTCTTACTAGAACGGGTCTGACGGTGA
GGGGCGAAAGCTGGGGGCGCGAACCGG

ASV_300
ACCGGCGGCCCGAGTGGTAGCCACTATTATTGGGTCTAAAGGGTCCGTAGCCGGTTC
GGTAAGTCCTCTGGAAAATCTGGTTGCTCAACAATCAGACTGCCAAGGGATACTGTC

GAACTTGAGACCGGGAGAGGTAAGAGGTACTTCAGGGGTAGGAGTGAAATCTTGTA
ATCCCTGGGGGACCATCTGTGGCGAAGGCGTCTTACCAGAACGGGTCTGACGGTGA
GGGACGAAAGCTGGGGGCGCGAACCGG

ASV_303
ACCGGCGGCTCGAGTGGTGGCCACTTTTATTGGGCTTAAAGCGTCCGTAGCTGGATT
TATAAGTCTCCTGGGAAATCCGCCGGCTTAACCGGCGGGCGTCCGGGAGATACTGTT
AATCTAGGGACCGGGAGAGGTGAGAGGTACTCCGGGGGTAGGAGTGAAATCCTGTA
ATCCCCGGGGGACCACCTGTGGCGAAGGCGTCTCACCAGAACGGCTCCGACAGTGA
GGGACGAAAGCTGGGGGAGCGAACCGG

ASV_305
ACCGGCGGCCCGAGTGGTAGCCGTTTTTATTGGGTCTAAAGGGTCCGTAGCCGGCCT
GATAAGTCCTTTGGGAAATCTGACAGCTTAACTGTCAGGCTTCTAAGGGATACTGTC
AGGCTTGGGACCGGAAGAGGTAAGGGGTACTCCAGGGGTAGGAGTGAAATCTTGTA
ATCCCTGGGGGACCATCTGTGGCGAAGGCGCCTTACCAGAACGGGTCCGACGGTGA
GGGACGAAAGCTAGGGGAGCAAACCGG

Deltaproteobacteria and Desulfofervidales

ASV_9
ACGGAGGGTGCAAGCGTTATTCGGAATTATTGGGCGTAAAGGGCGCGTAGGCGGTC
TTTTAAGTCAGATGTGAAAGCCCGGGGCTCAACCCCGGAAGTGCATTTGAAACTAA
GGGACTTGAGTATGGGAGAGGGAAGTGGAATTCCTGGTGTAGAGGTGAAATTCGTA
GATATCAGGAGGAACACCGGTGGCGAAGGCGACTTCCTGGACCAATACTGACGTTG
AGGCGCGAAGGCGTGGGGAGCAAACAGG

ASV_12
ACGCAGGTGGCAAGCGTTGCCCGGAATCACTGGGCGTAAAGGGTGCGTAGGCGGCT
GGACAAGTCGCAGGTTAAAGCCCGGGGCTCAACCCCGGAAAGGCCTGCGATACTGT
CTGGCTTGAGGGCCGGAGAGGCTGGCGGAATTCCCGGTGTAGGGGTGAAATCCGTA
GATATCGGGAGGAACACCAGTGGGGAAGCCGGCCAGCTGGACGGTCCCTGACGCTG
AGGCACGAAAGCGTGGGGAGCAAACCGG

ASV_14
ACGGAGGGTGCTAGCGTTGTTCGGAATTACTGGGCGTAAAGCGGGTGTAGGCGGTT
TGTTAAGTCAGATGTGAAAGCCCACGGCTCAACCGTGGAAGTGCATCTGAAACTGG
CAGACTTGAGTACCGGAGAGGGAAGTGGAATTCCTGGTGTAGGGGTGAAATCCGTA
GATATCAGGAGGAACACCGGTGGCGAAGGCGACTTCCTGGACGGATACTGACGCTG
AGATCCGAAAGCGTGGGGAGCAAACAGG

ASV_16
ACGGAGGGTGCAAGCGTTGTTCGGAATTACTGGGCGTAAAGCGCGCGTAGGTGGTC
TGTTATGTCAGATGTGAAAGTCCACGGCTCAACCGTGGAAGTGCATTTGAAACTGGC
AGACTTGAGTACTGGAGGGGGTAGTGGAATTCCCGGTGTAGAGGTGAAATTCGTAG

ATATCGGGAGGAATACCGGTGGCGAAGGCGACTACCTGGCCAGATACTGACACTGA
GGTGCGAAAGCGTGGGGAGCGAACAGG

ASV_22
ACGGAGGGTGCGAGCGTTACTCGGAATTACTGGGCGTAAAGGGCGCGTAGGCGGGA
AGGCAAGTTGAGCGTGTAAGCCTGAGGCTCAACCTCAGAATGGCGCTCAAAACTGC
CTTTCTTGAGTCCCGGAGAGGCCGGCGGAATTCCCGGTGTAGGGGTGAAATCCGTAG
ATATCGGGAGGAACACCGGTGGCGAAGGCGGCCGGCTGGACGGGTACTGACGCTGA
GGCGCGAAAGCGTGGGGAGCAAACAGG

ASV_34
ACGGAGGGTGCGAGCGTTGTTCGGAATTACTGGGCGTAAAGGGGATGTAGGCGGTT
TGTTAAGTCAGATGTGAAAGCCCACGGCTCAACCGTGGAAGTGCATCTGAAACTGG
CAGACTTGAGTACCGGAGAGGGAAGTGGAATTCCTGGTGTAGGGGTGAAATCCATA
GATATCAGGAGGAACACCGGTGGCGAAGGCGACTTCCTGGACGGATACTGACGCTG
AGATCCGAAAGCGTGGGGAGCAAACAGG

ASV_39
ACGGAGGGTGCAAACGTTGTTCGGAATCACTGGGCGTAAAGGGCGCGCAGGCGGTT
TGATTAGTCAGATGTGAAAGCCCACGGCTTAACCGTGGAAGTGCATTTGAAACTGTC
AGACTTGAGTACCAGAGGGGGTAGTGGAATTCCCGGTGTAGAGGTGAAATTCGTAG
ATATCGGGAGGAATACCGGTGGCGAAGGCGACTACCTGGCTGGATACTGACGCTGA
GGCGCGAAAGCGTGGGGAGCAAACAGG

ASV_42
ACGGGGGGCGCAAGCGTTATTCGGAATTATTGGGCGTAAAGGGCGTGTAGGCGGTC
TTATCGGTCAGATGTGAAAGCCCAGGGCTCAACCCTGGACGTGCATTTGAAACAGTA
AGACTTGAGTACTGGAGAGGAAAGCGGAATTCCTGGTGTAGAGGTGAAATTCGTAG
ATATCAGGAGGAACACCGATGGCGAAGGCAGCTTTCTGGACAGAAACTGACGCTGA
TGCGCGAAGGCGTGGGTAGCGAACAGG

ASV_46
ACGGAGGGTGCAAGCGTTACTCGGAATCACTGGGCGTAAAGGGCGCGCAGGCGGGA
AGGCAAGTTGAGCGTGTAAGCCTGAGGCTCAACCTCAGAATGGCGCTCAAAACTGC
CTTTCTTGAGTCCCGGAGAGGCCGGCGGAATTCCCGGTGTAGGGGTGAAATCCGTAG
ATATCGGGAGGAACACCGGTGGCGAAGGCGGCCGGCTGGACGGGTACTGACGCTGA
GGCGCGAAAGCGTGGGGAGCAAACAGG

ASV_49
ACGTAGGGTGCGAGCGTTGTTCGGAATTACTGGGCGTAAAGGGCGTGTAGGCGGTC
TGACAAGTCAAGAGTGAAAGTTATAGGCTCAACCTATAAAATGCTCTTGAAACTATC
AAACTTGGGTCCGGAAGAGGGAAGCGGAATTCCCGGTGTAGGGGTGAAATCCGTAG
ATATCGGGAGGAACACCAGCGGCGAAGGCGGCTTCCTGGGACGGTACCGACGCTGA
GGCGCGAAAGCGTGGGGAGCAAACAGG

ASV_51

ACGGAGGGTGCAAGCGTTATTCGGAATTATTGGGCGTAAAGGGCGCGTAGGCGGTC
TTTTAAGTCAGATGTGAAAGCCCGGGGCTCACCCCCGGAAGTGCATTTGAAACTAAG
GGACTTGAGTATGGGAGAGGGAAGTGGAATTCCTGGTGTAGAGGTGAAATTCGTAG
ATATCAGGAGGAACACCGGTGGCGAAGGCGACTTCCTGGACCAATACTGACGCTGA
GGCGCGAAGGCGTGGGGAGCAAACAGG

ASV_59
ACGGAGGGTGCAAGCGTTGTTCGGAATTATTGGGCGTAAAGAGCATGTAGGCGGTC
TGTTAAGTCTGGTGTGAAAGCCCGGGGCTCAACCCCGGAAGTGCATTGGATACTGGC
AGACTTGAGTATGGGAGAGGAAAGTGGAATTCCGAGTGTAGGAGTGAAATCCGTAG
ATATTCGGAGGAACACCAGTGGCGAAGGCGGCTTTCTGGACCAATACTGACGCTGA
GATGCGAAAGCGTGGGGAGCGAACAGG

ASV_69
ACGGAGGGTGCGAGCGTTGTTCGGAATTACTGGGCGTAAAGCGGGTGTAGGCGGTT
TGTTAAGTCAGATGTGAAAGCCCACGGCTCAACCGTGGAAGTGCATCTGAAACTGG
CAGACTTGAGTACCGGAGAGGGAAGTGGAATTCCTGGTGTAGGGGTGAAATCCGTA
GATATCAGGAGGAACACCGGTGGCGAAGGCGACTTCCTGGACGGATACTGACGCTG
AGATCCGAAAGCGTGGGGAGCAAACAGG

ASV_76
ACGGAGGGTGCAAGCGTTGTTCGGAATTACTGGGCGTAAAGCGCGCGTAGGTGGTC
TGTTATGTCAGATGTGAAAGTCCACGGCTCAACCGTGGAAGTGCATTTGAAACTGGC
AGACTTGAGTACTGGAGGGGGTAGTGGAATTCCCGGTGTAGAGGTGAAATTCGTAG
ATATCGGGAGGAATACCGGTGGCGAAGGCGACTACCTGGCCAGATACTGACACTGA
GGTGCGAAAGCGTGGGGAGCAAACAGG

ASV_108
ACGGAGGGTGCAAGCGTTATTCGGAATTATTGGGCGTAAAGGGCGCGTAGGCGGCC
TTTTAAGTCAGATGTGAAAGCCCGGGGCTTAACCCCGGAAGTGCATTTGAAACTAAA
GGGCTTGAGTATGGGAGAGGGAAGTGGAATTCCTGGTGTAGCGGTGAAATGCGTAG
ATATCAGGAGGAACACCGGTGGCGAAGGCGACTTCCTGGACCAATACTGACGCTAA
GGCGCGAAGGCGTGGGTAGCAAACAGG

ASV_120
ACGGAGGGTGCAAGCGTTATTCGGATTTATTGGGCGTAAAGAGCGTGTAGGCGGTT
GGGATAGTCAGACGTGAAAGCCTTCTGCTCAACAGAAGAAGTACGTCTGAAACTGC
CCAACTTGAGTACGAGAGAGGAAAGTGGAATTCCCAGTGTAGAGGTGAAATTCGTA
GATATTGGGAGGAACACCTGTGGCGAAGGCGACTTTCTGGATCGATACTGACGCTG
AGACGCGAAAGCGTGGGTAGCAAACAGG

ASV_123
ACGGGGGGTGCAAGCGTTATTCGGAATTACTGGGCGTAAAGAGCGCGTAGGCGGTC
TTTTAAGTCAGGTGTGAAAGCCCGGGGCTCAACCCCGGAAGTGCACTTGAAACTAA
GGGACTTGAGTATGGGAGAGGGAAGTGGAATTCCTGGTGTAGCGGTGAAATGCGTA
GATATCAGGAGGAACACCGGTGGCGAAGGCGACTTCCTGGACCAATACTGACGCTG

AGGCGCGAAGGCGTGGGGAGCAAACAGG

ASV_125
ACGGAGGGTGCAAGCGTTGTTCGGATTTACTGGGCGTAAAGAGCGTGTAGGCGGTT
TAGCAAGTCAGATGTGAAAGCCCTGGGCTTAACCCGGGAAGTGCATTTGAAACTGC
TTCACTAGAGTATGGGAGAGGAGATTGGAATTCCTGGTGTAGAGGTGAAATTCGTA
GATATCAGGAGGAACACCCGTGGCGAAGGCGATTCTCTGGACCAATACTGACGCTG
AGACGCGAAAGCGTGGGGAGCAAACAGG

ASV_127
ACGGGGGGCGCAAGCGTTATTCGGAATTATTGGGCGTAAAGGGCGCGTAGGCGGTC
TTGTCGGTCAGATGTGAAAGCCCAGGGCTCAACCCTGGACGTGCATTTGAAACAGC
AAGACTTGAGTACGGGAGAGGAAAGCGGAATTCCTGGTGTAGAGGTGAAATTCGTA
GATATCAGGAGGAACACCGATGGCGAAGGCAGCTTTCTGGACCGATACTGACGCTG
AGGCGCGAAGGCGTGGGTAGCGAACAGG

ASV_131
ACGGGGGGTGCGAGCGTTATTCGGAATTATTGGGCGTAAAGGGCGCGTAGGCGGCC
GCTTAAGTCAGGCGTGAAAGCCCGGGGCTTAACCTCGGAAGTGCGTTTGAAACTGA
GCGGCTTGAGTATGGGAGAGGGAAGCGGAATTCCTGGTGTAGAGGTGAAATTCGTA
GATATCAGGAGGAACACCTGTGGCGAAGGCGGCTTCCTGGACCAATACTGACGCTG
AGGCGCGAAGGCGTGGGGAGCAAACAGG

ASV_145
ACGGAGGGCGCGAGCGTTGTTCGGAATCACTGGGCGTAAAGCGGGTGTAGGCGGCC
AAGTAAGTCGGGTGTGAAAGCCCGAGGCTCAACCTCGGAAGGGCACTCGAAACTGC
TTGGCTTGAGGACGGGAGAGGGAGGTGGAATTCCCGGTGTAGCGGTGAAATGCGTA
GATATCGGGAGGAACACCGGTGGCGAAGGCGGCCTCCTGGCCCGTCCCTGACGCTG
AGACCCGAAAGCGTGGGGAGCAAACAGG

ASV_147
ACGGAGGGTGCAAGCGTTACTCGGAATTACTGGGCGTAAAGGGCGCGTAGGCGGGA
AGGCAAGTTGAGCGTGTAAGCCTGAGGCTCAACTTCAGAATGGCGCTCAAAACTGC
CTTTCTTGAGTCCCGGAGAGGCCGGCGGAATTCCCGGTGTAGGGGTGAAATCCGTAG
ATATCGGGAGGAACACCGGTGGCGAAGGCGGCCGGCTGGACGGGTACTGACGCTGA
GGCGCGAAAGCGTGGGGAGCAAACAGG

ASV_149
ACGGGGGGCGCAAGCGTTATTCGGAATTATTGGGCGTAAAGGGCGCGTAGGCGGTT
TTGTCGGTCAGATGTGAAAGCCCAGGGCTCAACCCTGGACGTGCATTTGAAACAGC
AAGACTTGAGTACGGGAGAGGAAAGCGGAATTCCTGGTGTAGAGGTGAAATTCGTA
GATATCAGGAGGAACACCGATGGCGAAGGCAGCTTTCTGGACCGATACTGACGCTG
AGGCGCGAAGGCGTGGGTAGCGAACAGG

ASV_154
ACGGGGGGTGCAAGCGTTATTCGGAATTATTGGGCGTAAAGGGCGCGTAGGCGGTC

TCTTAAGTCAGATGTGAAAGCCCGGGGCTTAACCCCGGAAGTGCATTTGAAACTAA
GGGACTTGAGTATGGGAGAGGGAAGTGGAATTCCTGGTGTAGAGGTGAAATTCGTA
GATATCAGGAGGAACACCGGTGGCGAAGGCGACTTCCTGGACCAATACTGACGCTG
AGGCGCGAAGGCGTGGGGAGCAAACAGG

ASV_162
ACGGAGGGTGCAAGCGTTGTTCGGAATTACTGGGCGTAAAGAGCGTGTAGGCGGTT
TGGCAAGTCAGATGTGAAAGCCCCGGGCTCAACCCGGGAAGTGCATTTGAAACTGC
TATACTTGAGTATGGGAGAGGAGAGTGGAATTCCCAGTGTAGAGGTGAAATTCGTA
GATATTGGGAGGAACACCCGTGGCGAAGGCGACTCTCTGGACCAATACTGACGCTG
AGACGCGAAAGCGTGGGGAGCAAACAGG

ASV_163
ACGGAGGGTGCAAGCGTTGTTCGGAATTATTGGGCGTAAAGAGCATGTAGGCGGAC
TATTAAGTCTGGTGTGAAAGCCCGGGGCTCAACCCCGGAAGTGCATTGGATACTGGT
AGTCTTGAGTATGGGAGAGGAAAGTGGAATTCCGAGTGTAGGAGTGAAATCCGTAG
ATATTCGGAGGAACACCAGTGGCGAAGGCGGCTTTCTGGACCAATACTGACGCTGA
GATGCGAAAGCGTGGGGAGCGAACAGG

ASV_172
ACGGAGGGTGCAAGCGTTACTCGGAATTACTGGGCGTAAAGGGCGCGCAGGCGGGA
AGGCAAGTTGAGCGTGTAAGCCTGAGGCTCAACCTCAGAATGGCGCTCAAAACTGC
CTTTCTTGAGTCCCGGAGAGGCCGGCGGAATTCCCGGTGTAGGGGTGAAATCCGTAG
ATATCGGGAGGAACACCGGTGGCGAAGGCGGCCGGCTGGACGGGTACTGACGCTGA
GGCGCGAAAGCGTGGGGAGCAAACAGG

ASV_189
ACGGGGGGTGCAAGCGTTATTCGGATTTATTGGGCGTAAAGGGCGCGTAGGCGGTC
TTTTAAGTCAGATGTGAAAGCCCGGGGCTTAACCCCGGAAGTGCATTTGAAACTAAG
GGACTTGAGTATGGGAGAGGGAAGTGGAATTCCTGGTGTAGAGGTGAAATTCGTAG
ATATCAGGAGGAACACCGGTGGCGAAGGCGACTTCCTGGACCAATACTGACGCTGA
GGCGCGAAGGCGTGGGGAGCAAACAGG

ASV_193
ACGGGGGGTGCAAGCGTTGTTCGGAATTACTGGGCGTAAAGGGCGCGTAGGCGGCC
ATTTAAGTCAGATGTGAAAGCCCACGGCTCAACCGTGGAACTGCATTTGAAACTGG
ATGGCTTGAGTACGGGAGAGGGAAGTGGAATTCCAAGTGTAGAGGTGAAATTCGTA
GATATTTGGAGGAACACCAGTGGCGAAGGCGGCTTCCTGGACCGATACTGACGCTG
AGGCGCGAAAGCGTGGGGAGCAAACAGG

ASV_208
ACGGAGGGTGCAAGCGTTGTTCGGAATTATTGGGCGTAAAGAGCGTGTAGGCGGTC
CGGTAAGTCAGATGTGAAAGCCCTGGGCTCAACCCAGGAAGTGCATTTGAAACTAC
CAGACTTGAGTACGGGAGAGGAGGGGGGAATTCCCGGTGTAGAGGTGAAATTCGTA
GATATCGGGAGGAATACCAGTGGCGAAGGCGCCCCTCTGGACCGATACTGACGCTG
AGACGCGAAAGCGTGGGGAGCAAACAGG

ASV_215
ACGGAGGGTGCAAGCGTTATTCGGATTTACTGGGCGTAAAGGGCGCGTAGGCGGTC
TGATAAGTCAGTTGTGAAAGCCCGGGCTCAACCCCGGAAGTGCAATTGAAACTGT
CAGGCTTGAGTATGGTAGAGGGAAGTGGAATTCCTGGTGTAGCGGTGAAATGCGTA
GATATCAGGAGGAACACCGGTGGCGAAGGCGGCTTCCTGGACCAATACTGACGCTG
AGGCGCGAAGGTGTGGGTAGCAAACAGG

ASV_216
ACGGGGGGTGCAAGCGTTATTCGGAATTATTGGGCGTAAAGGGCGCGTAGGCGGTC
TTGTCGGTCAGATGTGAAAGCCCAGGGCTCAACCCTGGACGTGCATTTGAAACAGC
AAGACTTGAGTACGGGAGAGGAAAGCGGAATTCCTGGTGTAGAGGTGAAATTCGTA
GATATCAGGAGGAACACCGATGGCGAAGGCAGCTTTCTGGACCGATACTGACGCTG
AGGCGCGAAGGCGTGGGTAGCGAACAGG

ASV_217
ACGCAGGTGGCGAGCGTTGCCCGGAATCACTGGGCGTAAAGGGTGCGTAGGCGGCT
GGACAAGTCGCAGGTTAAAGCCCGGGCTCAACCCCGGAAAAGCCTGCGATACTGT
CTGGCTTGAGGGCCGTAGAGGCTGGCGGAATTCCCGGTGTAGGGGTGAAATCCGTA
GATATCGGGAGGAACACCAGTGGGGAAGCCGGCCAGCTGGGCGGTCCCTGACGCTG
AGGCACGAAAGCGTGGGGAGCAAACCGG

ASV_219
ACAGAGGGTGCGAACGTTGCTCGGATTTACTGGGCGTAAAGCGCGTGTAGGCGGAC
TCGCAAGTCGGTTGTGAAATCCCTGGGCTTAACCTAGGAACTGCATCCGAAACTGCT
TGTCTTGAGTAATGGAGAGGGTGGCGGAATTCCCGGTGTAGAGGTGAAATTCGTAG
ATATCGGGAGGAACATCAGTGGCGAAGGCGGCCACCTGGACATTTACTGACGCTGA
GACGCGAAAGCGTGGGGAGCAAACAGG

ASV_228
ACGGGGGGCGCAAGCGTTATTCGGAATTATTGGGCGTAAAGGGCGCGTAGGCGGTC
TTGTCGGTCAGATGTGAAAGCCCAGGGCTCAACCCTGGACGTGCATTTGAAACAGC
AAGACTTGAGTACGGGAGAGGAGAGCGGAATTCCTGGTGTAGAGGTGAAATTCGTA
GATATCAGGAGGAACACCGATGGCGAAGGCAGCTCTCTGGACCGATACTGACGCTG
AGGCGCGAAGGCGTGGGTAGCGAACAGG

ASV_251
ACAGAGGGTGCAAACGTTGCTCGGATTTACTGGGCGTAAAGCGCGTGTAGGCGGAC
TCGCAAGTCGGTTGTGAAATCCCTGGGCTTAACCTAGGAACTGCATCCGAAACTGCG
TGTCTTGAGTAATGGAGAGGGTGGCGGAATTCCCGGTGTAGAGGTGAAATTCGTAG
ATATCGGGAGGAACATCAGTGGCGAAGGCGGCCACCTGGACATTTACTGACGCTGA
GACGCGAAAGCGTGGGGAGCAAACAGG

ASV_254
ACGGAGGGTGCGAGCGTTGTTCGGAATTACTGGGCGTAAAGCGGGTGTAGGCGGTT
TGTTAAGTCAGATGTGAAAGCCCACGGCTCAACCGTGGAAGTGCATCTGAAACTGG

CTGACTTGAGTACCGGAGAGGGAAGTGGAATTCCTGGTGTAGGGGTGAAATCCGTA
GATATCAGGAGGAACACCGGTGGCGAAGGCGACTTCCTGGACGGATACTGACGCTG
AGATCCGAAAGCGTGGGGAGCAAACAGG

ASV_255
ACGGAGGGTGCAAGCGTTGTTCGGAATTACTGGGCGTAAAGGGCGCGTAGGCGGCT
GTTTAAGTCAGATGTGAAAGCCCTCGGCTTAACCGAGGAACTGCATTTGAAACTGGA
CGGCTTGAGTACGGGAGAGGGAAGTGGAATTCCTGGTGTAGAGGTGAAATTCGTAG
ATATCAGGAGGAACACCGGTGGCGAAGGCGGCTTCCTGGACCGATACTGACGCTGA
GGCGCGAAAGCGTGGGTAGCAAACAGG

ASV_259
ACGGGGGGTGCAAGCGTTATTCGGAATTATTGGGCGTAAAGGGCGCGTAGGCGGTT
TTGTCGGTCAGATGTGAAAGCCCGGGGCTCAACCCTGGAAGAGCATTTGAAACAGC
AAGACTTGAGTACGGGAGAGGAAAGCGGAATTCCTGGTGTAGAGGTGAAATTCGTA
GATATCAGGAGGAACACCGATGGCGAAGGCAGCTTTCTGGACCGATACTGACGCTG
AGGCGCGAAGGCGTGGGTAGCGAACGGG

ASV_281
ACGGGGGGTGCAAGCGTTGTTCGGAATTATTGGGCGTAAAGAGCGTGTAGGCGGCC
AGGTAAGTCGGATGTGAAAGTCCCGGGCTCAACCCGGGAAGTGCATTTGAAACTGC
TTGGCTAGAGTAAGAGAGAGGAAAGTGGAATTCCTGGTGTAGAGGTGAAATTCGTA
GATATCAGGAGGAACACCGGTGGCGAAGGCGACTTTCTGGCTCTATACTGACGCTG
AGACGCGAAAGCGTGGGTAGCAAACAGG

ASV_289
ACGGAGGGTGCAAGCGTTACTCGGAATTACTGGGCGTAAAGTGCGCGTAGGTGGGA
AGGTAAATTAAGCGTGAAAGCCTAGGGCTCAACCCTAGAATTGCGCTTAAAATTGC
CTTCCTTGAGTCTTGGAGAGGGAGGCGGAACTCCCGGTGTAGGGGTGAAATCCGTA
GATATCGGGAGGAACACCGGTGGCGAAGGCGGCCTCCTGGCCAAGTACTGACGCTG
AGGCGCGAAAGCGTGGGGAGCAAACAGG

ASV_293
ACGGAGGGTGCGAGCGTTACTCGGAATCACTGGGCGTAAAGGGCGCGTAGGCGGGA
AGGCCAGTTGAGCGTGTAAGCCTGAGGCTTAACCTCAGAATGGCGCTCAAAACTGC
CTTTCTTGAGTCCCGGAGAGGCCGGCGGAATTCCCGGTGTAGGGGTGAAATCCGTAG
ATATCGGGAGGAACACCGGTGGCGAAGGCGGCCGGCTGGACGGGTACTGACGCTGA
GGCGCGAAAGCGTGGGGAGCAAACAGG

ASV_301
ACGGAGGGTGCAAGCGTTGTTCGGAATTACTGGGCGTAAAGGGCGTGCAGGCGGGT
TGGTAAGTCAGATGTGAAAGCCCTGGGCTTAACCCAGGAAGTGCATTTGAAACTGC
CATCCTAGAGTATGGGAGAGGAGAGTGGAATTCCCAATGTAGAGGTGAAATTCGTA
GATATTGGGAGGAACACCCGTGGCGAAGGCGACTCTCTGGACCAATACTGACGCTG
AGACGCGAAAGCGTGGGGAGCAAACAGG

ASV_302
ACGGAGGGTGCAAGCGTTACTCGGAATTACTGGGCGTAAAGGGCGCGTAGGCGGGA
AGGCAAGTTGAGCGTGTAAGCCTGAGGCTTAACCCCAGAATGGCGCTCAAAACTGC
CTTTCTTGAGTCCCGGAGAGGCCGGCGGAATTCCCGGTGTAGGGGTGAAATCCGTAG
ATATCGGGAGGAACACCAGTGGCGAAGGCGGCCGGCTGGACGGGTACTGACGCTGA
GGCGCGAAAGCGTGGGGAGCAAACAGG
